# CGGBP1 regulates CTCF occupancy at repeats

**DOI:** 10.1186/s13072-019-0305-6

**Published:** 2019-09-23

**Authors:** Divyesh Patel, Manthan Patel, Subhamoy Datta, Umashankar Singh

**Affiliations:** HoMeCell Lab, Biological Engineering, Indian Institute of Technology Gandhinagar, Gandhinagar, Gujarat 382355 India

## Abstract

**Background:**

CGGBP1 is a repeat-binding protein with diverse functions in the regulation of gene expression, cytosine methylation, repeat silencing and genomic integrity. CGGBP1 has also been identified as a cooperator of histone-modifying enzymes and as a component of CTCF-containing complexes that regulate the enhancer–promoter looping. CGGBP1–CTCF cross talk in chromatin regulation has been hitherto unknown.

**Results:**

Here, we report that the occupancy of CTCF at repeats depends on CGGBP1. Using ChIP-sequencing for CTCF, we describe its occupancy at repetitive DNA. Our results show that endogenous level of CGGBP1 ensures CTCF occupancy preferentially on repeats over canonical CTCF motifs. By combining CTCF ChIP-sequencing results with ChIP sequencing for three different kinds of histone modifications (H3K4me3, H3K9me3 and H3K27me3), we show that the CGGBP1-dependent repeat-rich CTCF-binding sites regulate histone marks in flanking regions.

**Conclusion:**

CGGBP1 affects the pattern of CTCF occupancy. Our results posit CGGBP1 as a regulator of CTCF and its binding sites in interspersed repeats.

## Introduction

Human CGGBP1 is a ubiquitously expressed protein with important functions in stress response, cell growth, proliferation and mitigation of endogenous DNA damage [1–5]. The CGGBP1 gene is conserved only in the amniotes with more than 98% similarity across the homeotherms [1]. Yet, the involvement of CGGBP1 in widely conserved cellular processes, such as cell cycle, maintenance of genomic integrity and cytosine methylation regulation, suggests that CGGBP1 fine-tunes these processes in homeothermic organisms to meet the challenges of their terrestrial habitats. CGGBP1 has no known paralogs in the human genome and is widely expressed in human tissues [6]. RNAi against CGGBP1 causes G1/S or G2/M arrest [3] and heat shock response-like gene expression changes with variable effects in different cell lines [3, 7]. CGGBP1 acts as a *cis*-regulator of transcription for tRNA genes, Alu elements [4], FMR1, CDKN1A, HSF1 [8, 9] and cytosine methylation-regulatory genes including DNMT1 [9]. However, none of these functions explain the widespread effect that CGGBP1 depletion has on the global transcriptome. In cultured normal human fibroblasts, CGGBP1 depletion results in gene expression shutdown in a manner that resembles the effects of serum starvation [4]. The mechanisms through which CGGBP1 regulates the genome and the transcriptome remain enigmatic.

Recent reports have shown that CGGBP1 regulates cytosine methylation genome-wide with the maximum methylation-regulatory effects at Alu and LINE elements in the CpG context [9]. The highly prevalent CHH cytosines, however, show a CGGBP1-dependent methylation pattern at GC-skew regions, insulators and enhancers [10]. Interestingly, CGGBP1 depletion causes an increase in CHH methylation at insulators (characterized as the CTCF-binding sites) and a decrease at enhancers. These findings suggest a cross talk between CGGBP1 and CTCF. There are additional evidences to support the possibility that CGGBP1 regulates the activities of insulators and enhancers. Most prominently, a targeted identification of proteins which structure the enhancer–promoter loops identified CGGBP1 as a partner of CTCF and YY1 [11]. Despite its ubiquitous expression, CGGBP1 has not been studied further by Weintraub and co-workers because, unlike YY1, CGGBP1 has not been identified as a hit in a screen for essential genes [12]. Canonically, CTCF binds with high affinity to well-defined sequence motifs [13–15]. However, it has been reported that CTCF is also associated with additional genomic elements that do not contain these motifs. Interspersed repeats SVA and Alu-SINEs [16] as well as microsatellite repeats serve as binding sites for CTCF [17–19] and CGGBP1. Even CTCF-binding sites that contain the CTCF-binding motifs, and are not repeats per se, have evolved from Alu-SINEs and related repetitive elements [20]. Additionally, CGGBP1 and CTCF both exhibit cytosine methylation-sensitive DNA binding at GC-rich sequences [21]. Further indications of cross talk between CTCF and CGGBP1 are derived from findings that both of these proteins interact with a crucial tumor suppressor factor NPM1 [22, 23]. CGGBP1 is a proven regulator of rRNA genes that contain CGG triplet repeats and localize to the nucleoli [8]. NPM1–CTCF interactions determine the organization of chromatin in the nucleolus. CTCF and NPM1 establish transcriptionally silent chromatin domains in the nucleolar periphery [23]. NPM1–CTCF interaction is required for insulator activity at many sites in the genome. Notably, NPM1 also complexes with CGGBP1 [22]. CGGBP1 drives rRNA synthesis upon growth factor stimulation by silencing Alu repeats [4]. Both CGGBP1 and CTCF are reported to have nuclear expression in interphase cells and midbody expression in mitotic cells [3, 24]. Collectively, these facts further strengthen the possibility of a functional cross talk between CGGBP1 and epigenomic regulator factors such as CTCF.

In this study, we have investigated this functional cross talk between CTCF and CGGBP1. We show that a fraction of endogenously expressed CGGBP1 and CTCF interact with each other. A systematic analysis of published ChIP-seq data for CGGBP1 and CTCF ChIP-seq data (ENCODE) show that the binding sites for the two proteins are in close proximity. Our co-immunoprecipitation (co-IP) assays lend support to these findings. Subsequently, by studying CTCF occupancy genome-wide through ChIP-seq under conditions of normal CGGBP1 expression, CGGBP1 knockdown and overexpression, we show that CTCF binds to both repeats and canonical CTCF motifs. Our analysis reveals that CGGBP1 levels determine the CTCF-binding preference between repeats and canonical CTCF motifs. By combining CTCF ChIP-seq with histone modification ChIP-seq under conditions of normal or depleted levels of CGGBP1, we have done a functional analysis of CGGBP1-regulated CTCF-binding sites. We show that a subset of these sequences are located at sites of sharp transitions in H3K9me3 levels. Our results present evidence for potential chromatin barrier activities of repeats that depend on the CGGBP1-CTCF axis.

## Results

### CGGBP1 and CTCF colocalize and interact with each other

**Fig. 1:**
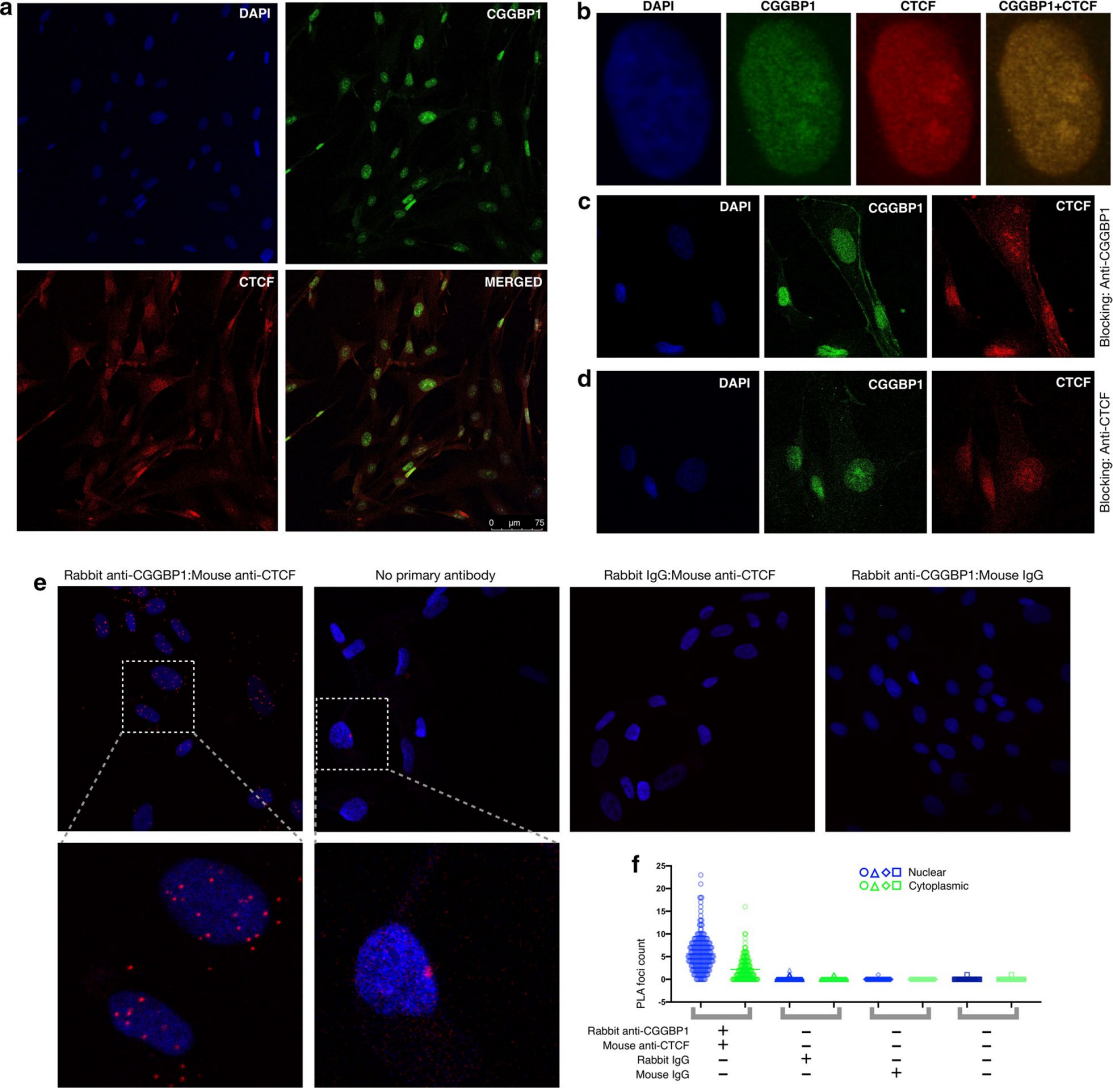
CGGBP1 and CTCF colocalize in the nucleus. **a** Human juvenile fibroblasts were co-immunostained for CGGBP1 (green) and CTCF (red). Nuclei were counterstained with DAPI (blue). **b** Zoomed view of a representative nucleus (630× zoom, focal plane thickness 0.896 micrometers) shows some overlap between signals for DAPI, CGGBP1 and CTCF. **c** Blocking was done with anti-CGGBP1 rabbit polyclonal antibody followed by incubation with detection antibody (anti-CGGBP1 rabbit polyclonal antibody and anti-CTCF mouse monoclonal antibody). **d** Blocking was done with anti-CTCF mouse monoclonal antibody followed by incubation with detection antibody (anti-CGGBP1 rabbit polyclonal antibody and anti-CTCF mouse monoclonal antibody). Micrographs show signal for DAPI (blue), CGGBP1 (green) and CTCF (red). **e** PLA (red foci) shows the proximity between CTCF and CGGBP1 in situ. Nuclei were stained with DAPI (blue). CGGBP1–CTCF proximity was stronger in the nuclei than cytoplasm (inset of rabbit anti-CGGBP1:mouse anti-CTCF sample). No significant PLA signal was observed in IgG and negative controls with no-primary antibody (inset of no-primary antibody sample). **f** Counting of PLA signals shows proximity detected using specific antibodies as significantly higher than the negative controls. Also the PLA signal frequency in the nuclei (blue) was higher than in the cytoplasm (green). All images were captured with confocal planes of 1.601 µm

To study the possibilities of functional cross talk between CGGBP1 and CTCF, we tested the subcellular colocalization of the two proteins. Using immunofluorescence (IF) in human fibroblasts, we observed that endogenous CGGBP1 as well as CTCF predominantly localized to the nuclei (Fig. 1a and b). By analyzing the normalized IF signals for CGGBP1 and CTCF, we observed that the expression of the two proteins was more correlated in the nuclei than in the cytoplasm (Additional file 1: Table S1). In additional IF experiments, we established that CTCF and CGGBP1 colocalize in the midbodies [5, 24] (Additional file 2: Figure S1A) as well as to varying extents in the nuclei (Additional file 2: Figure S1B).

Co-expression and colocalization of two nuclear proteins in IF assays are not unexpected. We performed IF experiments to detect whether the proximity between CTCF and CGGBP1 exhibits steric hindrance on immunodetection by using a previously published protocol [25]. The results showed that blocking with CGGBP1 antibody sterically hindered immunodetection of CTCF (Fig. 1c and Additional file 1: Table S1). However, the blocking by CTCF antibody did not exert a strong steric hindrance on detection of CGGBP1 (Fig. 1d and Additional file 1: Table S1). The size difference between CTCF and CGGBP1 could explain why blocking by CGGBP1 antibody was stronger than that by CTCF antibody. To verify the colocalization of CGGBP1 and CTCF, we performed proximity ligation assays (PLA). By using two different antibody pairs for CTCF and CGGBP1, we established that these two proteins occur in close proximity (intermolecular distance less than 40 nm) (Fig. 1e and Additional file 2: Figure S2). Counting of PLA signal foci reinforced that CGGBP1 and CTCF colocalization was more prevalent in the nuclei (72.18%) than the cytoplasm (27.81%) (Fig. 1f). The patterns of our PLA signals for CGGBP1 and CTCF were comparable with the previously reported PLA results for CTCF and RAD21 [26], HOXA10 [27], CTCFL [28] and PARP1 [29].

**Fig. 2:**
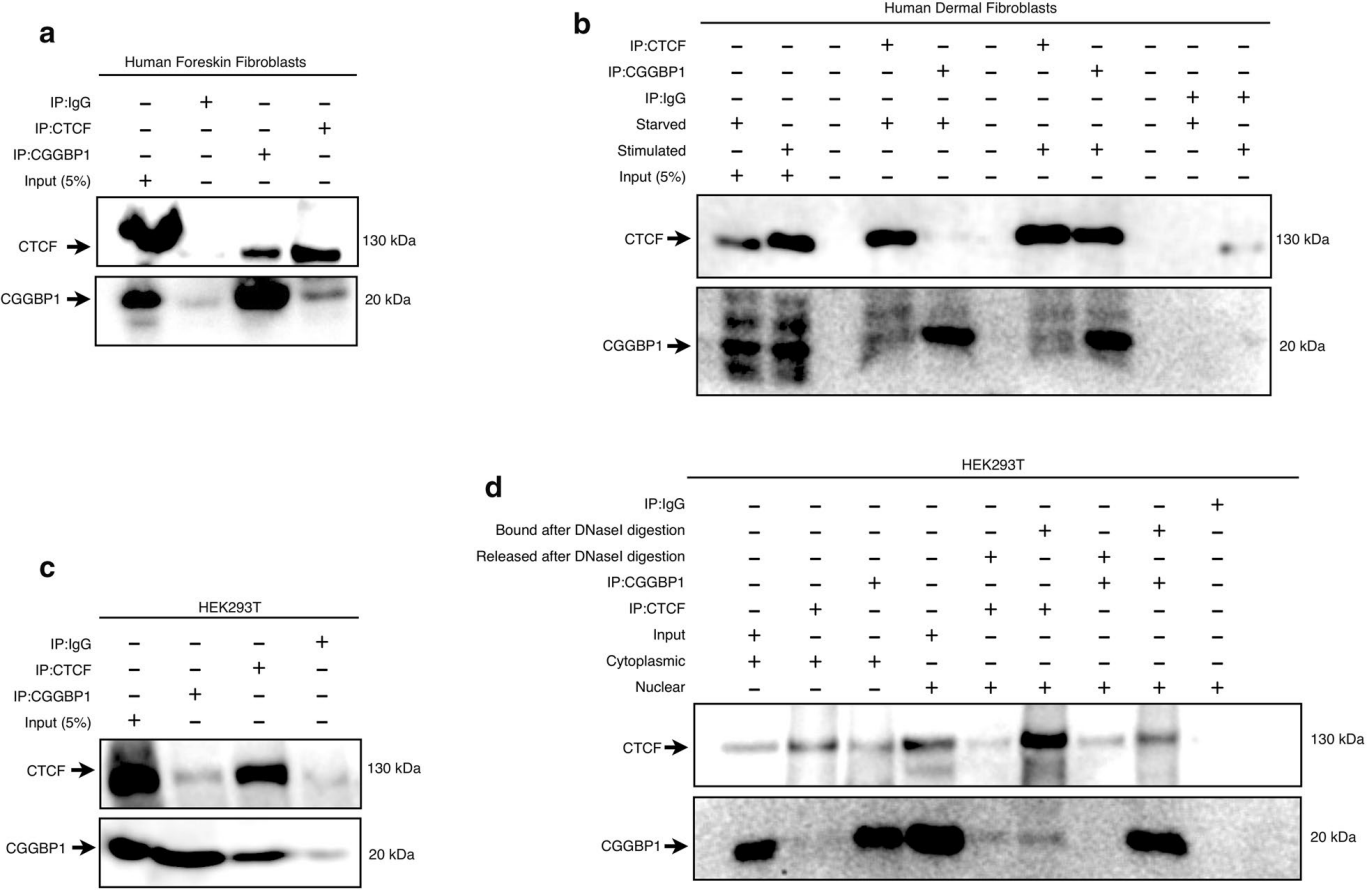
CGGBP1 and CTCF interact variously in different cell types and subcellular fractions. **a** Reciprocal co-IPs confirmed interaction between CGGBP1 and CTCF in human foreskin fibroblast. **b** Reciprocal co-IPs in human dermal fibroblast showed a different pattern of CGGBP1–CTCF interaction. CGGBP1 and CTCF co-IP was positive only when the cells were stimulated after 48 h of starvation. The interaction between CTCF and CGGBP1 was stoichiometrically different in these cells as the pull-down of CGGBP1 with CTCF antibody was much weaker than that of CTCF using CGGBP1 antibody. **c** CGGBP1–CTCF interaction was confirmed in HEK-293T cells by reciprocal co-IPs. **d** CGGBP1 and CTCF co-IP assays were performed in cytoplasmic and nuclear fractions of HEK-293T cells separately. CTCF–CGGBP1 pull-downs showed presence of a major protein–protein complex and a minor protein-DNA–protein complex. The purity of nuclear and cytoplasmic fractions was ascertained by using histone H3 (H3K4me3 and H3K27me3) and GAPDH, respectively as markers (Additional file 2: Figure S3)

To further characterize the interactions between endogenously expressed CGGBP1 and CTCF, we performed reciprocal co-immunoprecipitations (co-IPs) on whole cell lysates. CGGBP1 expression and function have been well studied in human foreskin fibroblasts [1]. Using CTCF antibodies, we could immunoprecipitate endogenous CGGBP1 in human fibroblasts (Fig. 2a). Reciprocally, using CGGBP1 antibodies, we could immunoprecipitate endogenous CTCF (Fig. 2a). The IF and PLA results suggested that the functional interactions between CTCF and CGGBP1 were expected more in the nuclear fraction than in the cytoplasmic fraction. CGGBP1 also shows enhanced nuclear presence in growth-stimulated cells as compared to serum-starved cells [4]. We thus performed co-IPs from human fibroblast lysates under conditions of serum starvation and stimulation. In the lysates from starved cells, the reciprocal pull-down of CTCF and CGGBP1 was very weak. In lysates from stimulated cells, however, using CGGBP1 antibody we could pull down a major fraction of CTCF (Fig. 2b). These findings suggested that the interactions between CTCF and CGGBP1 depend on whether the cells are growing or quiescent. This pattern of the co-IPs, wherein the pull-down of CTCF by anti-CGGBP1 was stronger than its reciprocal pull-down, was in agreement with the antibody blocking assays (Additional file 1: Table S1). To study the functional relevance of the proximity and the possible interactions between CTCF and CGGBP1, we employed knockdown of CGGBP1 using shRNA. For this, we chose to perform the next set of experiments in (HEK-293T) human embryonic kidney cells, as these cells satisfy two conditions: (i) they express CTCF and CGGBP1 at detectable levels, and (ii) CGGBP1 knockdown does not induce quiescence in these cells (unpublished observations). First, we performed co-IPs for endogenously expressed CTCF and CGGBP1 in HEK-293T cells and performed reciprocal pull-downs of CTCF and CGGBP1. We observed that similar to the findings obtained from the fibroblasts, the pull-down of CGGBP1 with anti-CTCF was weaker than the pull-down of CTCF with anti-CGGBP1 (Fig. 2c). We also subjected the co-IP pull-down from the HEK-293T nuclear fraction (Additional file 2: Figure S3) to DNase digestion and found that a fraction of the CGGBP1–CTCF complex depends on a DNA bridge (Fig. 2d). As a result, we found that there are two kinds of nuclear CGGBP1–CTCF complexes: one in which association of CTCF with CGGBP1 was indirect and depended on a DNA bridge and another in which it was a direct interaction independent of any DNA bridge. Collectively, these findings showed that in the nuclei, CGGBP1 and CTCF have close proximity with direct as well as indirect interactions between them. The presence of a DNA-mediated complex of CTCF and CGGBP1 suggested that these proteins could occupy nearby sites on DNA. CGGBP1 has been shown to bind in close proximity of CTCF [11], indicating a cross talk between CGGBP1 and CTCF.

Because CGGBP1 is a repeat-binding protein with widespread occupancy throughout the genome, there is a possibility of proximity between the binding sites of CGGBP1 and many other DNA-binding proteins in addition to CTCF. We analyzed the ENCODE transcription factor (TF) ChIP-seq datasets [4, 30] to determine whether the proximity between CTCF and CGGBP1 binding sites is stronger as compared to those between CGGBP1 and other TFs. A frequency distribution of distances between repeat-free ChIP-seq peaks of CGGBP1 and 20 different TFs (for which ChIP-seq data are available from primary cells) clearly showed that CTCF stands out as one of the top three TFs exhibiting most proximal binding to CGGBP1 (Additional file 2: Figure S4). The three TFs with the highest frequency of binding sites in proximity of the CGGBP1-binding sites included CTCF and CEBPB (Additional file 2: Figure S4A and B). Proximity between CEBPB- and CTCF-binding sites has been independently shown by previous reports [31–34]. Also, we have shown earlier that CGGBP1-binding sites in Alu repeats show similarity to the binding sites of CEBPB homolog CEBPZ [4]. The proximity between CTCF- and CGGBP1-binding sites was further supported by the findings that centrally enriched CTCF motifs (CTCFBSDB score > 3) were present in 56% of CGGBP1-binding sites [35]. We considered these findings as strong indications of a functional cross talk between CGGBP1 and CTCF and hypothesized that the genomic occupancies of CTCF and CGGBP1 could be regulated by each other. These mechanisms would rely on proximity between CGGBP1- and CTCF-binding sites on DNA, but would not necessarily depend on physical interactions between them. In this study, we have tested if CTCF occupancy depends on CGGBP1.

### CTCF occupancy preference for repeats over motifs depends on CGGBP1

**Fig. 3:**
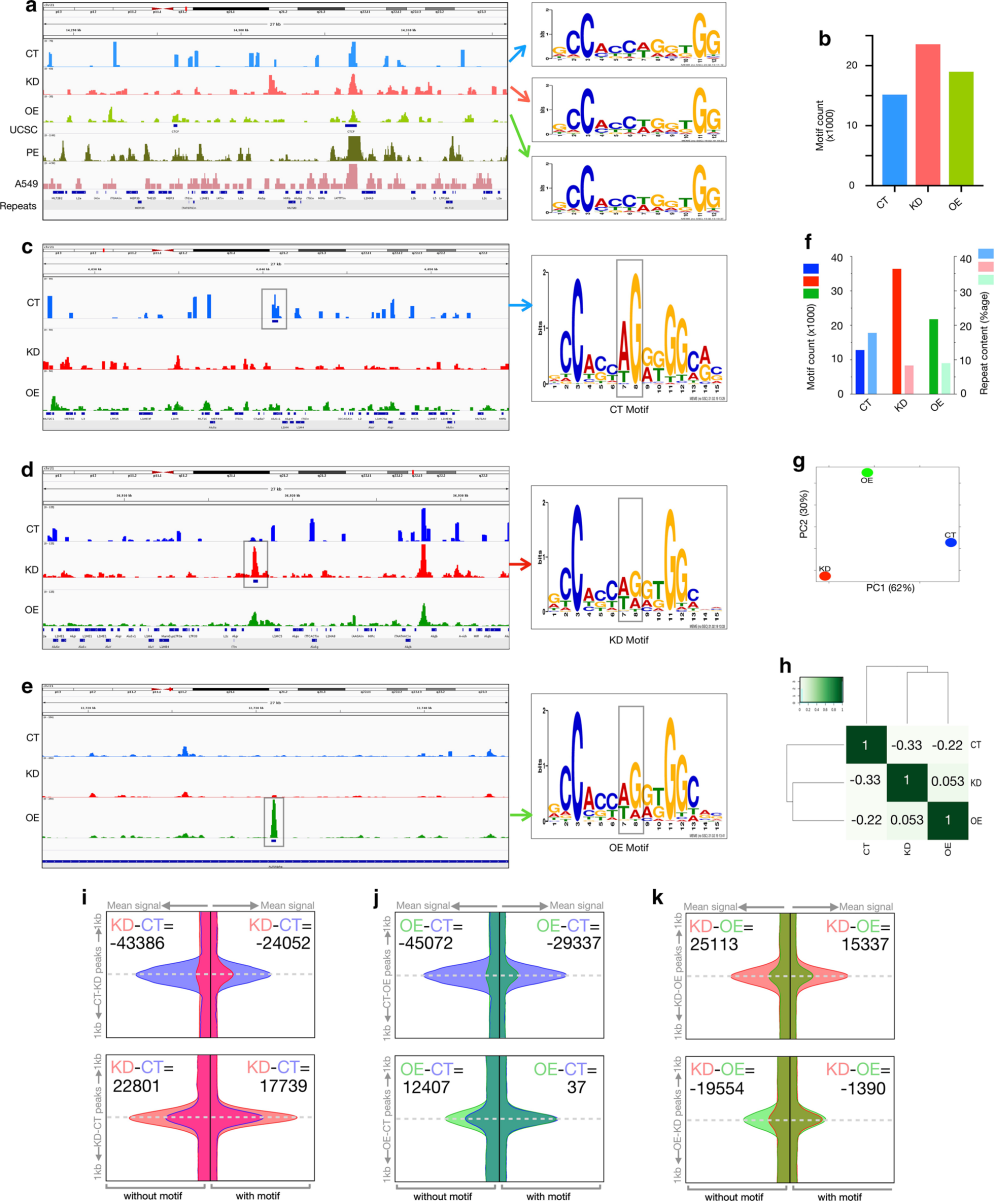
CTCF occupancy at CTCF motifs and repeats depends on CGGBP1. **a** Genome browser views showing repeat-masked (RM) CTCF reads distribution in CT, KD and OE samples in a region on chromosome 21. For comparison, UCSC CTCF-binding sites, CTCF reads from prostate epithelial cells (ENCFF098DGZ) and A549 (ENCFF9810JS) are shown. Widespread differences between cell types and similarities between CT, KD and OE can be observed. The motifs discovered in CT, KD and OE are shown on the right. The scales of the *Y*-axes are different between the samples. **b** CTCF occupancy at the indicated motifs (**a**) is low under normal levels of CGGBP1 with KD causing a strong increase. **c**–**e** Genome browser views showing CTCF reads distribution at repeat-unmasked CTCF peaks exclusive for CT (**c**) (*Y*-axis data range 0–50, 27 kb region on chr21 approximately start from 36908 kb), KD (**d**) (*Y*-axis data range 0–125, 27 kb region on chr21 approximately start from 6028 kb) and OE (**e**) (*Y*-axis data range 0–250, 27 kb region on chr21 approximately from 12718 kb). CTCF peaks and subtle differences in CTCF motifs enriched in each dataset are shaded in gray. **f** CTCF motif counts and repeat content in repeat-unmasked CTCF peaks were plotted for CT, KD and OE samples, respectively. Between CT and KD, a striking shift in CTCF occupancy from repeats to motifs is seen. **g** PCA analysis between the ChIP samples to find the patterns of differences in CT, KD and OE datasets due to CGGBP1 levels showed that CT was nearly equidistant and different from KD (majorly PC1) and OE (majorly PC2) with no similarity between KD and OE. **h** Clustering and correlation heatmap for CT, KD and OE samples show that CT is weakly inversely correlated with KD and OE with no correlation between KD and OE. **i**–**k** CTCF reads signal was plotted for CT (blue), KD (red) and OE (green) in 1 kb flanks of peaks center. Differential peaks as described below were split into without motifs (left flanks) or with motifs (right flanks) and plotted separately. The peak identities are mentioned on the left *Y*-axis of each block. The sample from which signal is derived is mentioned in the inset for area under the curve difference calculation. Peak identities are as follows: CT-positive KD-negative peaks (CT–KD peaks), KD-positive CT-negative peaks (KD–CT peaks), CT-positive OE-negative peaks (CT–OE peaks), OE-positive CT-negative peaks (OE–CT peaks), OE-positive KD-negative peaks (OE–KD peaks), KD-positive OE-negative peaks (KD–OE peaks). The labels on the upper left and upper right of each block indicates the area under the curve (AUC) difference calculated by comparing the read signals plotted for KD and CT (KD–CT) for Fig. 3i, OE and CT (OE–CT) for Fig. 3j and KD and OE (KD–OE) for Fig. 3k. AUC differences show that repeat-containing motif-free peaks are specifically enriched in CT the most, and the differences between CT, KD and OE are lowest at motif-containing peaks. The horizontal axes showing the mean signals have the following range of magnitudes: 0–100 units for “with motifs” and 0–60 units for “without motifs”

To understand how CGGBP1 affects genome-wide occupancy of CTCF, we performed CTCF ChIP-seq in HEK-293T cells with different levels of CGGBP1. HEK-293T cells were transduced with the following lentiviruses: non-targeting shRNA (CT), CGGBP1-silencing shRNA (KD) and CGGBP1-overexpressing (OE) cDNA construct (Additional file 2: Figure S5). We mapped the quality-filtered sequencing reads (Phred quality value ≥ 20, [36]) to repeat-masked hg38 (Additional file 1: Table S2) and called peaks. The CT, KD and OE peaks were consistent with previously described CTCF-binding sites in various cell types including HEK-293 (Fig. 3a and Additional file 2: Figure S6). These results showed that the overall CTCF occupancy at robust repeat-free CTCF-binding sites was not affected by CGGBP1 levels.

A total of 26,635, 24,418 and 21,071 peaks were called in CT, KD and OE, respectively (Additional file 1: Table S3). As expected, the peaks were rich in CTCF motifs (Fig. 3b). Motif identification using MEME on CT, KD and OE peak sequences returned only one motif (e value limit of 3.8e-147) that corresponded to the HoCoMoCo CTCF motif. Interestingly, the number of CTCF motifs per peak was lowest in CT. The occurrence of this motif was highest in KD, followed by the OE peaks (Additional file 1: Table S3). These findings established the validity of our ChIP-sequencing assays and showed that the binding of CTCF to its canonical motifs is affected by the levels of CGGBP1.

Since these peaks were called using reads aligned only to the non-repeat parts of hg38, any occurrence of repetitive sequences was unexpected. However, we observed that even the repeat-masked CT, KD and OE peaks contained short sequences which showed unexpected similarity to L1 repeats (Additional file 2: Figure S7 and Additional file 1: Table S4). These results supported the observations made on repeat-free CGGBP1 and CTCF peaks. The occurrence of these L1-matching sequences per peak was highest in CT (4.3) compared to KD and OE (2.63 and 2.98, respectively). The presence of L1-matching sequences in CTCF ChIP-seq peaks strongly indicated that bona fide repeats pulled down with CTCF could have gone undetected due to repeat-masked alignments of sequence reads.

Next, we aligned the CTCF ChIP-seq reads from CT, KD and OE to repeat-unmasked hg38 and called peaks to analyze the CTCF occupancy patterns (Fig. 3c–e) with respect to repeats. A comparison of mappability differences between CTCF-bound sequences in hg38 with or without repeat masking is provided in the Additional file 1: Table S5. As shown in Additional file 2: Figure S8, alignment to the repeats was performed without any loss of alignment scores. A comparison of signals within the repeats with those in immediate flanks of repeats suggests a slight loss of signal at repeats due to elimination of reads that may not have been uniquely aligned (Additional file 2: Figure S9). The chances of misalignment to repeats were strongly constrained by applying two necessary conditions in bowtie2: (i) a zero gap and a mismatch-free alignment of 22 bases long seed region, and (ii) retention of only the highest scoring alignment as the true alignment. We also ensured that the mapping of reads to repeats was not an alignment artifact by ensuring that the repeat content did not increase post-alignment. In random samples of sequences from pre-alignment and post-alignment datasets, we found that the interspersed repeat contents (SINEs and LINEs) were comparable (Additional file 2: Figure S10). Alignment did not increase the difference in repeat content between CT and KD reads. However, the CT and KD sequence reads which aligned to repeats were dispersed differently. This resulted in differential identification of repeat-containing peaks between CT and KD. The repeat-containing peaks derived from unmasked reads were strongly decreased in KD compared to CT (Additional file 2: Figure S10). As an additional measure, we picked reads mapping entirely within LINEs from ten different locations in the genome, extracted their sequence from the raw sequence data and subjected them to the nucleotide BLAST against hg38. The alignment revealed by BLAST for all of these sequences exactly matched the bowtie2 alignment for all the reads (Additional file 1: Table S6). These analyses established that our alignment procedure was well controlled and reliable.

Interestingly, the peaks that we identified by mapping to unmasked hg38 also showed a very specific association with sequence reads of some ENCODE CTCF ChIP-seq datasets (Additional file 2: Figure S11). These results showed that the CT, KD and OE peaks identified on unmasked hg38 were genuine binding sites for CTCF, which include repeats. The CT peaks derived from alignment to unmasked hg38 contained the maximum interspersed repeats and the least number of CTCF motifs as compared to those of KD and OE (Additional file 1: Tables S7 and S8). Even after including the repeats in alignment and peak calling, the canonical CTCF-binding motifs remained the most frequently occurring motif (Fig. 3c–e, Additional file 1: Table S8). The CTCF motif was present in more than 25% sequences in all the samples, whereas the next most frequently occurring motif was present in approximately 10% or fewer sequences (Additional file 1: Table S8). The motifs identified in CT, KD and OE datasets were very similar to each other (albeit with different levels of occurrence with KD having the highest motif content) with a subtle prominence of G at the eighth position of the motif in CT (regions in the motifs highlighted in Fig. 3c–e). The knockdown as well as overexpression of CGGBP1 reduced the repeat occupancy and enhanced the motif occupancy of CTCF (Fig. 3f). The reads appeared relatively dispersed in KD and OE and clustered in CT. A comparison of the dispersion of reads in units of 0.5 kb showed that the number of read-free regions was higher in CT than in KD or OE. In both KD and OE, the frequency of zero read density was reduced, and the distribution curves shifted centrally toward moderate read density (Additional file 2: Figure S12A). These results indicated that changes in the levels of CGGBP1 altered CTCF occupancy in KD and OE such that the frequency of regions with moderate enrichment was increased.

We could conclude so far that CGGBP1 depletion and overexpression both caused a redistribution of CTCF occupancy. To further understand how the samples CT, KD and OE differed from each other, we measured the differences between the samples using principal component analysis (PCA). We analyzed the variation between the CTCF peaks by using the input as a control. We could establish that two principal components (Additional file 2: Figure S12B) together accounted for a total of 82% of the variation between the samples. The major component (PC1) separated KD and OE in two different directions from the input (located at zero distance from itself). The second component (PC2) clustered the KD and OE closer to each other, recapitulating the read density similarity between them (Additional file 2: Figure S12B). A second PCA between the ChIP samples (without the input) showed that CT, KD and OE were equidistant from each other such that KD and OE differed from CT in two different directions, implying that although the read densities were similar between OE and KD, the patterns of CTCF occupancy differed between them (Fig. 3g). Hierarchical clustering using DiffBind in R [37] placed the uncorrelated samples KD and OE in one unrelated cluster separated from the inversely correlated sample CT (Fig. 3h). Thus, overexpression and knockdown of CGGBP1 showed two different and opposing effects on overall CTCF occupancy.

To further understand the nature of the differences in CTCF occupancy between CT, KD and OE, we compared read distributions in 1 kb flanks of peaks exclusive to each sample using one sample as a negative control at a time. In CT-positive KD-negative peaks (CT–KD peaks), the background occurrence of KD reads was higher in motif-containing peaks (ΔAUC KD–CT = − 24,052 for motifs and − 43,386 for repeats) (Fig. 3i), whereas the specific presence in CT and absence in KD were tightly associated with repeat-containing reads. In KD-positive CT-negative peaks (KD–CT peaks), the background CT reads were present in peaks with and without motifs both (a much reduced ΔAUC KD–CT = 17,739 for motifs and 22,801 for repeats) (Fig. 3i). These findings meant that CGGBP1 favors the occupancy of CTCF at repeat-rich motif-free peaks and the knockdown of CGGBP1 led to a reduction in CTCF occupancy at repeats. Interestingly, compared to the CT-positive OE-negative peaks (CT–OE peaks), the OE-positive CT-negative peaks (OE–CT peaks) showed a different kind of change in read distribution. OE–CT peaks had an increase in OE-specific reads only at peaks without motifs with negligible difference at motif-containing peaks (Fig. 3j). A comparison of ΔAUC values of KD–CT peaks and OE–CT peaks showed that CGGBP1 depletion in KD favors CTCF occupancy at CTCF motifs, whereas CGGBP1 overexpression in OE drives CTCF occupancy at regions which are repeat rich and motif poor. Finally, a reciprocal comparison between OE and KD exclusive peaks confirmed this again (Fig. 3k). While KD exclusive peaks showed higher CTCF occupancy at regions with and without motifs both, the OE exclusive peaks showed enhanced CTCF occupancy at regions without motifs only (a much reduced ΔAUC KD–OE = − 1390 for motifs and − 19,554 for repeats). An interpretation of these results in the light of repeat contents of the motif-containing and motif-free exclusive peaks (Additional file 1: Tables S9 and S10) established that (i) CTCF binds to repeat-rich motif-poor as well as motif-rich repeat-poor regions, (ii) knockdown of CGGBP1 shifts the binding from repeats to the motifs, and (iii) overexpression of CGGBP1 exerts an opposite effect and shifts the binding to repeat-rich regions.

Consistent with the above findings, measuring of repeat content in CT, KD and OE peaks (Additional file 1: Table S7) showed that CTCF-bound sites were excessively motif rich in KD and OE and motif poor, but L1 rich in CT (Additional file 1: Table S8). Interpreted alongside the data shown in Additional file 2: Figure S10, these results show that although comparable levels of repeats are pulled down in CT and KD samples, the repeat-containing reads are scattered such that they yield fewer peaks in KD as compared to CT. We concluded that although repeat-masked CT, KD and OE peaks all have CTCF motif as the consensus binding site, there is genuine CTCF binding at repeats and that this binding is different between CT and KD. Thus, it seems that CTCF normally occupies repeats and motifs both, but when CGGBP1 levels are disrupted, the occupancy is adversely affected at repeats. This shifts the balance of CTCF occupancy toward motif richness in KD. In addition to the single CTCF motifs, the 2× CTCF motifs [28] were also found in KD (1727 occurrences) at a higher frequency than in CT (159 occurrences).

Identification of CGGBP1-binding sites at repeat-masked regions had shown that CGGBP1 binds to multiple small L1-matching sequences which could not be identified as repeats by RepeatMasker (Additional file 2: Figure S7B). We argued that if CGGBP1- and CTCF-binding sites occur in proximity with each other, then these L1-matching sequences will continue to occur in the CT, KD and OE peaks. Indeed, the short L1-matching sequences (Additional file 2: Figure S4) were found with comparable frequencies in CT, KD and OE peaks derived from alignment to unmasked hg38 (Additional file 1: Table S11). The occurrence of these short L1-matching motifs was specific, because it was diminished just by a shuffling of letter positions with unchanged PWM weights (Additional file 1: Table S4). To demonstrate the patterns of CTCF occupancy at repeat-driven peaks and motif-driven peaks, we plotted the ChIP signals in the immediate flanks of CTCF motif-positive LINE-negative peaks and CTCF motif-negative LINE-positive peaks (Additional file 2: Figure S13). All these signals from the latter showed a distribution of reads such that the tails of the peaks traversed through the regions of transition between repeats and non-repeat flanks. These results reinforced that the repeat-containing or repeat-free peaks both have similar expected patterns of read pileup.

Given these findings, we concluded that CGGBP1 regulates the genomic occupancy pattern of CTCF through the proximity of L1-matching CGGBP1-binding sites to CTCF-binding sites. Next, we wanted to identify the functional effects of the changes in CTCF occupancy upon changes in CGGBP1 levels.

### CGGBP1 level affects CTCF occupancy at known insulators

To determine the effects of altered CTCF occupancy caused by changes in CGGBP1 level, we first analyzed the disturbances in CTCF occupancy at genomic locations annotated as regulatory elements (UCSC Regulation datasets). CTCF binding at the regulatory elements influences genome organization and function with direct effects on epigenetic states of the chromatin. Read density measurements at regulatory regions showed strong enhancements of CTCF occupancy at replication origins (Additional file 2: Figure S14A) and enhancers (Additional file 2: Figure S14B) by CGGBP1 overexpression.

**Fig. 4:**
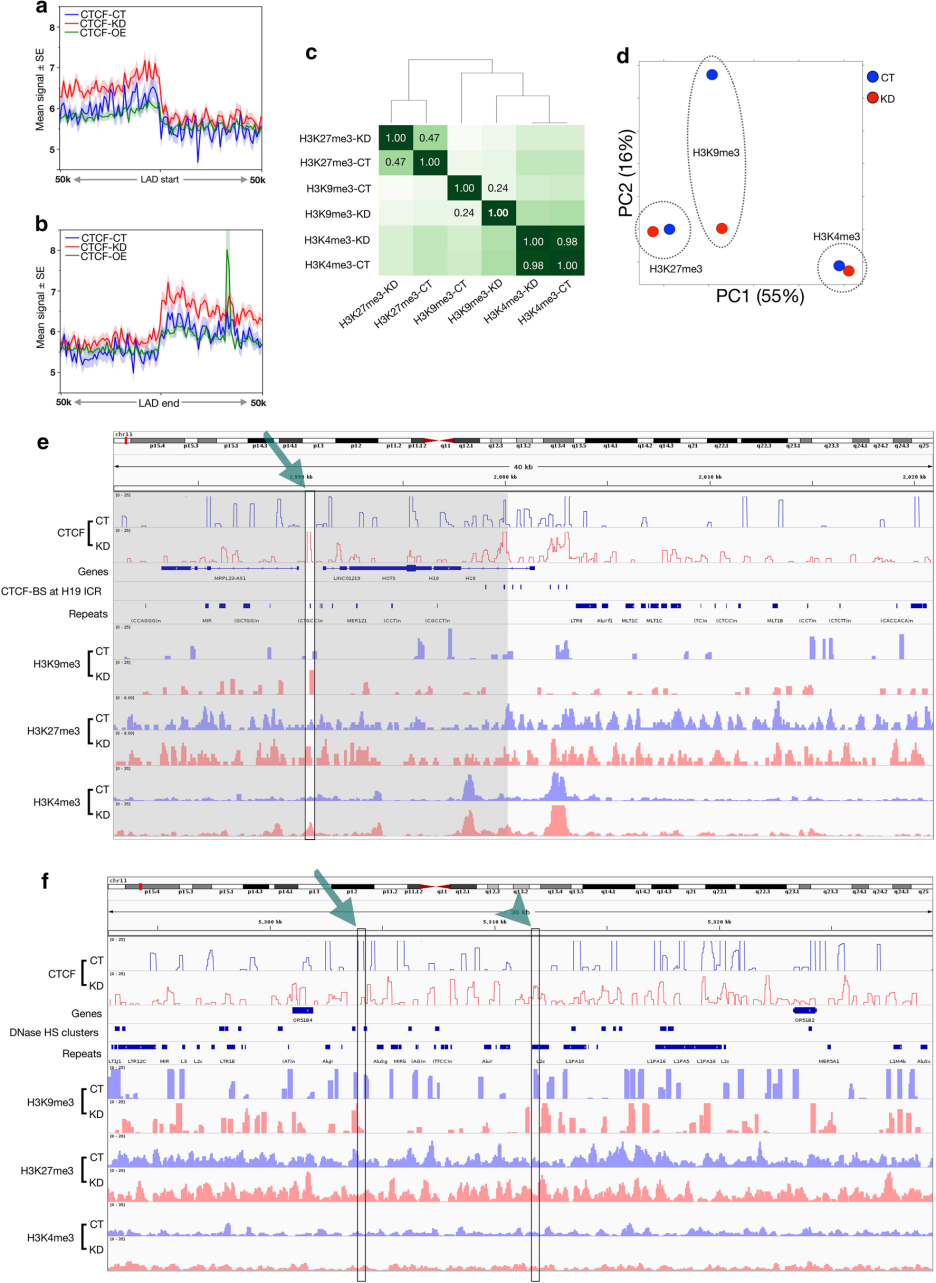
CGGBP1 affects CTCF occupancy and histone modifications at known CTCF-binding sites. **a**, **b** CTCF read distribution was plotted for CT, KD and OE at the LAD boundaries in the 50 kb flanks with bins of 1 kb. The average read density with standard error of the mean was plotted for LAD start sites (**a**) and LAD end sites (**b**). CTCF binding is reported to occur in 10 kb flanks of the LAD start and end sites. **c** Correlation heatmap was plotted for H3K4me3, H3K9me3 and H3K27me3 ChIP-seq peaks in CT and KD. The correlation values (*r*) corresponding to CT and KD are mentioned for each histone modification in the heatmap. Unlike H3K4me3 and H3K27me3, the CT and KD samples for H3K9me3 were significantly different from each other. **d** PCA analysis using input as control shows the distance between H3K9me3 CT and KD as the most affected in KD. CGGBP1 depletion minimizes the distinction of H3K9me3 from H3K4me3 and H3K27me3 along PC2 only while along PC1, the two repressing marks remain closer to each other than the activating mark. **e** Disturbances in the H3K9me3 is observed in KD compared to CT. Genome browser views (a 40 kb region on chr11 starting from approximately 1983 kb) showing reads of CTCF (data range on *Y*-axis 0–25), H3K9me3 (data range on *Y*-axis 0–25), H3K27me3 (data range on *Y*-axis 0–8) and H3K4me3 (data range on *Y*-axis 0–35) for CT and KD along with the CTCF-binding sites and repeats at the H19-ICR locus. The gain of CTCF binding upon CGGBP1 knockdown is highlighted by green arrow and changes in the histone modification profile in 10 kb flanks are highlighted by gray boxes. **f** Genome browser view (36 kb region on chr11 starting from approximately 5294 kb) of the beta-globin locus control region undergoing change in chromatin barrier function upon CGGBP1 depletion. The plot contains tracks of CTCF reads (data range on *Y*-axis 0–25), H3K9me3 (data range on *Y*-axis 0–25), H3K27me3 (data range on *Y*-axis 0–20) and H3K4me3 (data range on *Y*-axis 0–35) reads, hg38 genes, DNase hypersensitivity clusters and repeats. The gain and loss of CTCF binding upon CGGBP1 loss of function are highlighted with an arrowhead and an arrow, respectively

Topologically associating domains (TADs) have been described for HEK-293 cells [38]. We analyzed the CTCF occupancy at these TADs in CT and KD. CGGBP1 depletion caused a loss of CTCF occupancy at 1128 TADs that were rich in CTCF-binding sites in CT (2929 peaks, Additional file 1: Table S12). On the other hand, in KD there was a gain of CTCF occupancy at 390 TADs (129 peaks, Additional file 1: Table S12). These findings suggested that new TADs may be formed in KD due to CTCF-binding site rearrangements. The lamina-associated domains (LADs), identified in Tig3ET cells [39], are conserved between different cell types. The LADs showed a consistent reduction in CTCF binding at the LAD start and end sites in KD as compared to CT and OE (Fig. 4a and b). The LADs are rich in L1 repeats [40], and thus a loss of binding in KD was expected.

Although the OE system is invaluable for testing the dependence of CTCF occupancy on CGGBP1 levels, we argued that it is more artifactual than KD for studying the functional outcome of the disruption in CGGBP1-dependent CTCF–DNA binding. The functional analyses henceforth were restricted to CT and KD only. We analyzed the effects of KD on CTCF occupancy at regulatory elements by comparing it with those of CT. These analyses were complemented with genome-wide measurements of three different histone modifications in CT and KD samples: H3K4me3, H3K9me3 and H3K27me3 (Additional file 1: Table S13). Unsupervised clustering and PCA of the three histone modification patterns in CT and KD showed that CGGBP1 knockdown disrupted H3K9me3 the most, with only minor effects on H3K4me3 and H3K27me3 (Fig. 4c and d).

Of all the regulatory regions that we analyzed, we observed changes in CTCF occupancy accompanied by histone modification changes at LADs- and CTCF-binding sites (motif-dependent sites located in repeat-free regions, called UCSC CTCF sites). CTCF loss of occupancy at LADs was accompanied by a mild, but consistent loss of the repeat-silencing mark H3K9me3 (Additional file 2: Figure S15A and B). At UCSC CTCF sites, the gain of CTCF binding in KD was accompanied by strong increases in transcription activating histone mark H3K4me3 with only mild changes in H3K9me3 and H3K27me3 marks (Additional file 2: Figure S16A). However, the UCSC CTCF sites that were repeat poor and CTCF motif rich had the maximum differences between CT and KD for H3K4me3 occupancy (Additional file 2: Figure S16A and B). H3K9me3 is a histone mark that occurs at repetitive sequences. Therefore, we investigated if the differences in CTCF and H3K9me3 occupancy between CT and KD occur predominantly at repeats and repeat-derived regulatory regions.

The insulators in the beta-globin locus and H19-ICR are known CTCF-binding sites. We first analyzed the patterns of CTCF occupancy in CT and KD at these two candidate regions.

At the three CTCF-binding sites located upstream of H19 gene, the most distal site upstream of the transcription start site (TSS) showed the strongest presence of CTCF. The three upstream binding sites, including the intervening regions between them, showed a conspicuous increase in CTCF occupancy in KD. This change in the CTCF occupancy pattern was also concomitant with a change in chromatin marks from bivalent (co-occurrence of H3K9me3 and H3K4me3) in CT to active (only H3K4me3) in KD due to a loss of H3K9me3 (Fig. 4e). Similar changes were observed at the downstream CTCF-binding sites where CGGBP1 knockdown led to an increase in CTCF occupancy. Further downstream from the H19 gene, there were distinct sites of gain and loss of CTCF occupancy in KD. A comparison of histone marks in 10 kb flanks showed that the asymmetry of H3K9me3 maintained on two sides of a binding site in CT was lost upon disruption of CTCF binding in KD. One such region where a gain of CTCF occupancy in KD (arrow) resulted in different levels of H3K9me3 in the flanks is highlighted in Fig. 4e under shadows. In the beta-globin LCR, CGGBP1 knockdown caused a disruption of CTCF occupancy such that the gain (arrowhead) as well as the loss (arrow) of occupancy in KD was observed at various DNase hypersensitivity sites. Interestingly the H3K9me3 signal in 10 kb flanks of these sites was symmetric in CT and became asymmetric in KD. Although there were changes in H3K27me3 and H3K4me3 signals as well, the asymmetric changes in the flanks of CGGBP1-dependent CTCF-binding sites was observed only for H3K9me3 (Fig. 4f). Cumulative signals of histone modification ChIP-seq from these regions are mentioned in Additional file 1: Table S14.

Of the two candidate regions analyzed here, H19-ICR is relatively poor in repeats than the beta-globin LCR and H19-ICR region also showed a weaker disturbance in H3K9me3 marks than beta-globin LCR region. These findings suggested that the change in the patterns of CTCF occupancy caused by CGGBP1 knockdown can affect chromatin barrier functions. It is also indicated that this CGGBP1-regulated chromatin barrier function through CTCF is predominant at repeats and thus affects the repeat-marking histone modification H3K9me3.

### CGGBP1 regulates chromatin barrier activity at motif-independent CTCF-binding sites in repeats

**Fig. 5:**
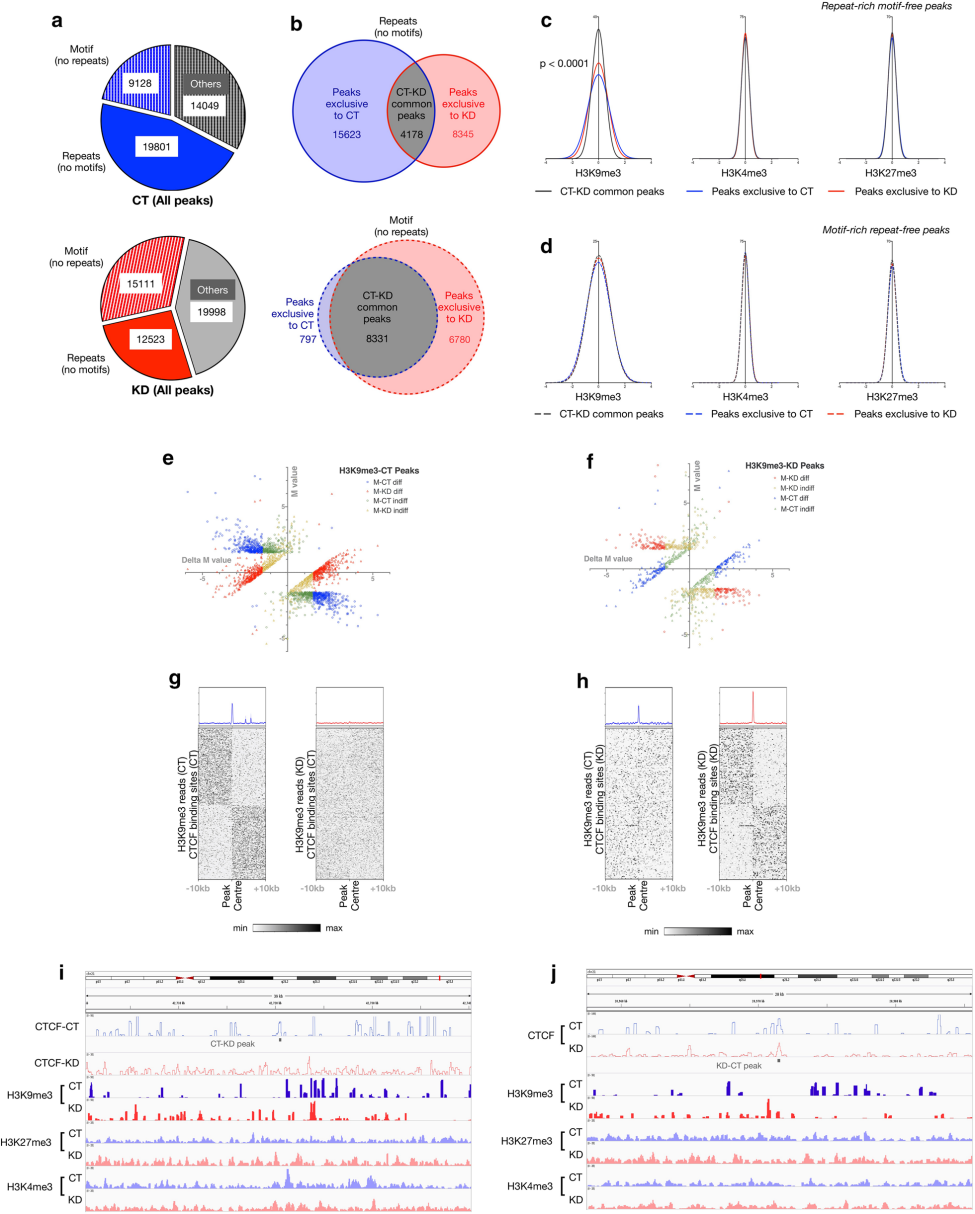
Changes in H3K9me3 occupancy upon CGGBP1 knockdown are tightly linked to motif-free repeat-rich CTCF-binding sites. **a** All CT (blue, top panel) and KD (red, bottom panel) peaks were classified into motif (no repeats) or repeats (no motifs) using FIMO and RepeatMasker tools. Peaks that could not be unambiguously classified were labeled as "Others" (gray) in both the groups. **b** Peaks exclusive to CT (blue) or KD (red) were determined in subsets of CT and KD peaks shown in **a**. Venn diagrams depicting shared or exclusive peaks between 9128 striped blue and 15111 striped red (shown in **a**) are shown on top in **b**. Venn diagrams depicting shared or exclusive peaks between 19,801 solid blue and 12,523 solid red (shown in **a**) are shown on bottom in **b**. These subsets of peaks help comparing CT and KD for features that vary between them depending on repeat or motif contents exclusively. **c**, **d** Frequency distribution of *M* values representing differences in H3K9me3, H3K27me3 and H3K4me3 read counts (upstream–downstream) 10 kb flanks for the six sets of peaks depicted in **b** in repeat-rich (no motif) peaks (**c**) and motif-rich (no repeat) peaks (**d**). Only H3K9me3 shows weak differences of CT and KD exclusives from common peaks for motif-rich (no repeat) peaks (**d**). H3K9me3 distribution differs strongly between CT and KD and deviates from common peaks at repeat-rich (no motif) peaks (**c**) only. **e**, **f** The *M* values depicted in H3K9me3 datasets (**d**) were separately analyzed for CT and KD to filter out peaks 10 kb flanks of which satisfy two conditions: (i) M value upstream–downstream difference > 1.5 and (ii) the Δ*M* [(*M* of KD) − (*M* of CT)] > 1.5 (**e**; red and blue spots) or Δ*M* [(*M* of CT) − (*M* of KD)] > 1.5 (**f**; red and blue spots). **g**, **h** Heatmaps of H3K9me3 signals from CT and KD datasets in the peaks corresponding to red-blue peaks in E and F show that there is a clear difference in H3K9me3 signal transitioning exactly at the peak centre where CTCF binding is observed and affected by CGGBP1 in KD. **g** and **h** show peaks exhibiting loss and gain of barrier activity, respectively. **i**, **j** Genome browser views showing loss of CTCF binding at a representative CT–KD peak that also exhibits loss of H3K9me3 barrier function upon CGGBP1 knockdown (**i**) [(39 kb region on chr21 starting from approximately 42700 kb), *Y*-axis represents data ranges 0–50 for CTCF CT, 0–35 CTCF KD, 0–50 for H3K9me3, 0–25 for H3K27me3 and H3K4me3]. A representative KD–CT exclusive peak that shows gain of CTCF binding upon CGGBP1 knockdown also displayed gain of H3K9me3 barrier function (**j**) [(28 kb region on chr21 starting from approximately 20908 kb], *Y*-axis represents data ranges 0–100 for CTCF, 0–50 for H3K9me3, 0–25 for H3K27me3 and H3K4me3)

Next, we analyzed the gain or loss of chromatin barrier functions at CGGBP1-dependent CTCF-binding sites. We hypothesized that repeat-rich regions that have CGGBP1-dependent CTCF-binding sites function as CGGBP1-dependent chromatin barrier sites as well. To test this possibility, we first identified CTCF-binding sites in CT and KD that we could unambiguously classify as motif-free repeats or repeat-free motifs (Fig. 5a). Any CGGBP1-dependent change in CTCF occupancy and hence on chromatin barrier function would be mostly limited to repeat-containing CTCF-binding sites that differ between CT and KD. By comparing CT/KD common peaks against the exclusive peaks of CT and KD, we sought to identify if indeed CGGBP1 selectively disrupts barrier functions of those CTCF-binding sites that are motif-free repeats (Fig. 5b).

For CT/KD common peaks and exclusive peaks of CT and KD, the differences in H3K4me3, H3K9me3 and H3K27me3 read counts in 10 kb upstream and downstream flanks were compared. Motif-free repeat-containing CT/KD common peaks were sites with a lesser barrier function as a distribution of the difference between upstream and downstream H3K9me3 signals at such peaks showed a low deviation from the normal of zero difference (Fig. 5b). The motif-free repeat-containing exclusive peaks of CT and KD on the other hand showed a broader distribution with significantly higher deviation from the normal (Fig. 5b). The distribution of H3K9me3 upstream–downstream difference for the CT–KD motif-free repeat-containing peaks were the most different from those of the CT/KD common peaks and were also significantly different from those of the KD–CT motif-free repeat-containing peaks. Strikingly, in the motif-containing repeat-free category, the H3K9me3 upstream–downstream difference distribution patterns of CT–KD and KD–CT peaks were not different from the CT/KD common ones (Fig. 5d). Furthermore, H3K4me3 and H3K27me3 upstream–downstream difference showed no difference between CT–KD, KD–CT and CT/KD common peaks (Fig. 5c and d). These findings demonstrated that H3K9me3 occupancy in the flanks of motif-free CTCF-binding sites in repeats is regulated by CGGBP1. Gain as well as loss of barrier function is observed at such regions upon CGGBP1 knockdown as CT–KD as well as KD–CT peaks showed significantly different upstream–downstream H3K9me3 signals compared to the CT/KD common peaks (Fig. 5c and d). Changes in barrier activity due to altered CTCF binding is the most likely reason for disruption in the H3K9me3 patterns.

To identify such regions, we selectively fished out the regions with strong gain or loss of CTCF binding (CT or KD exclusive peaks) that also exhibit a strong change in H3K9me3 occupancy in 10 kb flanks. Depending on the differences in signals, we applied arbitrary thresholds (Additional file 2: Figure S17) to select a subset of exclusive peaks that showed strong differences in upstream and downstream signals. Thus, upon CGGBP1 knockdown, the largest change in histone modification landscape was found to occur for H3K9me3 in the flanks of sites with CGGBP1-dependent CTCF occupancy. These potential chromatin barrier sequences were of two kinds: those which lost the normal CTCF occupancy in KD (Fig. 5e, g and i) and those which gained CTCF occupancy in KD (Fig. 5f, h and j). In comparison, CT or KD exclusive peaks with changes in H3K4me3 and H3K27me3 occupancy above threshold in 10 kb flanks were much fewer in number as well as with lower upstream–downstream differences (Additional file 2: Figure S18). In total, we identified 663 sites with a loss of barrier function and 216 sites with a gain of barrier function upon CGGBP1 depletion and a concomitant loss of CTCF occupancy.

These potential chromatin barrier elements with a loss of CTCF occupancy in KD (Fig. 5e and g) were the most repeat rich (35% L1 content), whereas those with a gain of CTCF occupancy in KD (Fig. 5f and h) were repeat poor and highly CTCF motif rich (Additional file 1: Table S15).

To extend the functional analysis from histone modifications to RNA levels, we first analyzed the co-variation of RNA levels in the same regions (shown in Fig. 5g and h) where H3K9me3 signals were found to be asymmetric across CGGBP1-dependent CTCF-binding sites. The location of H3K9me3-regulatory CGGBP1-dependent CTCF-binding sites was predominantly in gene-poor regions (Additional file 2: Figure S19). This precluded any direct assays of transcript levels of known genes as functional readouts of varying H3K9me3 levels. We argued that the asymmetry of H3K9me3 signals would be best reflected as asymmetry in the levels of transcripts upstream and downstream of the CGGBP1-dependent CTCF-binding sites in high-throughput RNA-seq data. Transcriptomic data from HEK-293 cells [38] (which resemble our CT sample the most) were analyzed to see if transcript levels also show asymmetry in the 10 kb flanks of the 663 regions that exhibit barrier activity in CT (regions shown in Fig. 5g and i). It was confirmed that the cumulative RNA-seq signals upstream and downstream of such barrier regions were strongly asymmetric and significantly different (Additional file 1: Table S16). Moreover, as expected, such a difference in RNA-seq signal was not observed for 216 regions which were found to function as chromatin barriers in the absence of CGGBP1 (Additional file 1: Table S16).

Thus, we concluded that CTCF-binding sites located in repeats or in proximity of repeats function as barriers between opposing patterns of H3K9me3. CTCF binding to such sites as well as the barrier function of these sites depend on CGGBP1. At the same time, there are sites to which de novo binding of CTCF takes place in the absence of CGGBP1. These anomalous CTCF-binding sites exhibit barrier activity in the absence of CGGBP1. Changes in CGGBP1 levels seem to alter the chromatin barrier function of repeat-derived candidate insulator sequences through regulation of CTCF occupancy.

## Discussion

Human CTCF is a multifunctional protein pivotal to the functional organization of chromatin [41]. Some well-established functions of CTCF are regulation of insulators and boundary elements, and regulation of topologically associating domains and higher-order chromatin structure [41]. CTCF, in complex with proteins including cohesin ring members, orchestrates chromatin structure and changes in CTCF binding have been shown to affect histone modifications and depend on cytosine methylation [20, 21, 41]. Given the important functions executed by CTCF, it makes evolutionary sense that there are regulatory cross talks between CTCF and other proteins. Of the nearly 70 protein interactants of CTCF (NCBI Gene), the mechanisms of interactions and functional cross talks between only some have been worked out. Some notable examples are SMCs [42], YY1 [11], BRD proteins [11, 43] and NPM1 [23]. Identification of new interactants and regulators of CTCF will lead to a deeper understanding of chromatin regulation and function. The work presented here shows that human CGGBP1 is a regulator of CTCF occupancy. We do not present any evidence for insulator activity for CTCF at these regions. However, the co-analysis of CTCF occupancy at repeats alongside the histone modification changes in CT and KD suggests that the CTCF–CGGBP1 axis affects a potential chromatin barrier activity at repeats.

The colocalization of CTCF with CGGBP1 in the nucleus is unsurprising, as both proteins have high concentration in the nucleus. However, colocalization at midbodies, the only extranuclear site of strong CGGBP1 and CTCF expression, suggests that functional cross talk between these two proteins might play a role in cytokinesis, abscission checkpoint and regulation of ploidy. Repetitive sequences prone to faulty homologous recombination can lead to chromosomal fusions [44–46]. Interestingly, the function of repeat-binding proteins such as CGGBP1, which delay cytokinetic abscission to resolve internuclear chromatin bridges, may cooperate with other midbody proteins for genomic integrity. The fact that CTCF is present at midbodies leads to an interesting possibility that similar to CGGBP1, CTCF may also contribute to genomic integrity through mechanisms similar to those of CGGBP1 [47]. In line with this possibility, we have observed a regulation of binding of CTCF at repeats by CGGBP1.

We have shown the interactions between CTCF and CGGBP1 through co-immunoprecipitation and PLA (supported by antibody-blocking IFs) on endogenously expressed proteins at native levels. This assumes significance because co-immunoprecipitation studies using overexpression systems are prone to detecting interactions that may not hold true at the much lower physiological concentrations of the two proteins [48]. PLA results, which showed that most of the proximity between CTCF and CGGBP1 exists in the nuclei, set the ground for the working hypothesis of the present work that a functional interplay between CGGBP1 and CTCF is required for proper DNA-binding and DNA-binding-dependent functions of the two proteins. There are supporting evidence for this possibility in the literature. BRD2 and CTCFL modulate boundary element function of CTCF by binding in the vicinity of CTCF even if physical association between these proteins may not exist [27, 28, 43]. The most direct evidence is the YY1–CTCF complex study, in which CGGBP1 emerged as a co-player with CTCF and YY1 for insulator and enhancer activity regulation [11]. The DNA binding of CGGBP1 in the vicinity of CTCF and YY1 leads to a possibility that in addition to a direct protein–protein complex, some interaction between these proteins could be mediated through a DNA bridge. Indeed, our co-immunoprecipitation assays in nuclear fractions with DNase digestion prove that a fraction of the CTCF–CGGBP1 co-immunoprecipitates is bridged by DNA. Since the co-IPs were performed in cleared lysates with high molecular weight DNA-protein complexes pelleted out and discarded, it implies that the DNA-bridged complexes of CTCF and CGGBP1 are not artifactual interactions mediated by long DNA molecules. However, the DNA-bridged interactions only account for approximately 34% of the total complex. Given that CGGBP1 binds to repetitive DNA, one interpretation of our co-IPs findings is that the larger protein CTCF occupies a set of specific sites on the repetitive DNA, whereas the small protein CGGBP1 binds to the same sequences through a physical complex with CTCF as also in the immediate vicinity of the CTCF-bound DNA. Thus, CGGBP1 could restrict CTCF occupancy to specific subsequences within the repeats by marking the borders of CTCF-binding sites at repeats. Our ChIP-seq results favor this possibility. An alternative possibility, indicated by the co-IPs and antibody blocking IFs, is that multiple CGGBP1 molecules (approximately 20 kDa) chaperone a much larger CTCF molecule (approximately 140 kDa) and thus regulate the CTCF–DNA interactions. Recently, a much smaller isoform of CTCF (approximately 50 KDa) has been described [49] and CGGBP1 may regulate the occupancy of the two forms of CTCF differently. In HEK-293T, the cells which we chose for our experiments, the expression of the shorter isoform of CTCF was very weak and the ChIP-seq data can be regarded as genome-wide occupancy of the longer form of CTCF mostly.

We chose to study CTCF–CGGBP1 cross talk in HEK-293T cells also because these cells have a strong enough expression of both the proteins to reason that a knockdown of CGGBP1 will have significant effects. In addition to having strong native expression of CGGBP1, HEK-293T cells showed an ability to tolerate a large change in the levels of CGGBP1; from a near-complete knockdown to approximately 7.7-fold overexpression. We have not observed such a tolerance for CGGBP1 levels in any other cell type earlier. The CTCF ChIP-seq data from HEK-293 (ENCODE dataset ENCFF183AAP, flagged for low read count) is thus a useful resource for comparison with our results.

The CTCF ChIP-seq in HEK-293T that we have performed shows a robust correlation with the CTCF ChIP-seq data available from other cell lines, thereby establishing the specificity of CTCF pull-down in our assays. This comparison with validated CTCF ChIP-seq datasets is necessary because we have mapped CTCF-bound DNA sequence reads to human genome with repeats as well as without repeats. By retaining only the best and uniquely mappable reads, we have ensured that any cross-matching of reads to repeat sequences was minimized. Both the repeat-masked and repeat-unmasked aligned read sets gave rise to distinct sets of peaks. The repeat-masked peaks were rich in the canonical CTCF motif [50], whereas the repeat-unmasked peaks were repeat rich and relatively motif poor. The CTCF-binding peaks from the published CTCF ChIP-seq datasets showed concentration of reads at our CTCF peaks obtained from repeat-unmasked (L1 rich and motif poor) as well as repeat-masked alignments (motif rich and L1 poor). This indicated that the CTCF peaks called in previously published studies [12] have not included the CTCF-binding sites at repeats and focused on peaks that contain CTCF-binding motifs. Interestingly, the CTCF motifs we observed in repeat-masked datasets were also seen in repeat-unmasked datasets albeit at a lower frequency. This result suggests that the binding of CTCF to repeats does not occur on consensus sequences which correspond to any repeat-specific sequence motifs. The preference for CTCF occupancy at repeats was dependent on CGGBP1, suggesting a cooperative association between CTCF and CGGBP1 for binding to the repeats or on CTCF motifs occurring within or in immediate proximity to the repeats. This observation would also justify the findings that CTCF-binding sites have evolved out of repeats, especially retrotransposons [15]. Our findings that short Alu- and L1-matching subsequences that were prevalent in CGGBP1-binding sites were also seen at considerable frequency in CTCF-binding sites lend further support to this argument.

One of the most important aspects of our findings is that the CTCF-binding sites in CT showed very strong pileups of reads as compared to KD, with relatively sharper transitions into read-free regions. In KD, the binding pattern of CTCF became more centrally enriched. The gradient ends of CTCF-binding sites in KD as compared to relatively blunter ends in CT suggest that CGGBP1 restricts and stabilizes CTCF occupancy at target sequences. This is reflected in the higher pileup of reads in CT compared to KD, without an increase in peak length. The presence of CTCF-binding sites inside and in the vicinity of repeats makes this more plausible, because binding of CGGBP1 to repetitive sequences flanking the CTCF target sites and motifs can constrain the binding of CTCF. A comparison of CTCF-binding sites [51–53] and CGGBP1-binding sites in fact shows proximity as well as overlaps at L1 and Alu repeats. In addition, proximity between CGGBP1 and CTCF binding has been shown at the CTCF-bound enhancer–promoter loops [11]. CGGBP1 thus seems to exert a qualitative effect on CTCF–DNA binding. CGGBP1 also regulates CTCF occupancy on LADs, TSSs and the sets of exclusive peaks that we have identified. CGGBP1 forms a complex with NPM1 [23] and the effects on LADs could be an outcome of CTCF–NPM1–CGGBP1 cross talk at nuclear lamina-associating sequences, often rich in L1 retrotransposons [40]. Interestingly, the overexpression of CGGBP1 changed CTCF occupancy pattern in two different ways. At most of the regions, it appeared similar to KD with a loss of sharper transitions from CTCF-bound to CTCF-unbound regions. At sites where CGGBP1 regulates cytosine methylation patterns such as replication origins and enhancers [10], the overexpression of CGGBP1 caused a drastic increase in CTCF occupancy. At the same regions, an opposite effect of loss of CTCF occupancy was observed in the absence of CGGBP1 in a range of 1 kb. Thus, KD and OE exert opposite effects on CTCF occupancy at loci where CGGBP1 regulates cytosine methylation. However, the similarities between CTCF occupancy pattern in two contrasting states of CGGBP1 knockdown and overexpression could be due to many reasons. CGGBP1 is required for CTCF occupancy at repeats and yet CGGBP1 itself is a repeat-binding protein. Excess amounts of CGGBP1 could potentially interfere with CTCF occupancy at repeats. CGGBP1 is a growth signal sensor protein that binds to the DNA depending on post-translational modifications [4]. The phosphorylation by receptor tyrosine kinases at Y20 and ATR at S164 seems to be essential for its nuclear localization and DNA binding, respectively. In the absence of signals that generate optimal post-translational modifications on CGGBP1, we have seen that its overexpression exerts a dominant negative effect [2, 4]. This is why the overexpression of WT CGGBP1 with insufficient post-translational modifications seems to mimic the effects of its knockdown partially. In addition, CGGBP1 itself is a repeat-binding protein and in OE the excess of CGGBP1 can compete for repeat occupancy with CTCF and effectively negate the occupancy of CTCF at repeats even if the repeat occupancy of CTCF depends on CGGBP1. Since overexpression systems are prone to artifacts due to toxicity of overexpression, we have restricted the use of the OE sample only to establish that genome-wide occupancy of CTCF depends on levels of CGGBP1. To study the functional outcome of CTCF dependence on CGGBP1, we restricted our investigation only to the CT and KD samples. These two samples allowed us to study the effects of CGGBP1 on CTCF without altering the stoichiometry of CGGBP1 limitlessly in an overexpression system. Thus, in the light of our findings that insulator sites in H19 and beta-globin loci underwent a change in CTCF occupancy upon CGGBP1 knockdown, we studied similar changes genome-wide upon CGGBP1 knockdown only.

The histone modifications H3K9me3, H3K4me3 and H3K27me3 are involved in transcriptional suppression of repetitive DNA, transcriptional activation (poised state of transcription with co-occurrence of H3K9me3, H3K4me3) and lineage-specific silencing of genes in the course of differentiation, respectively. CGGBP1 is a repeat-binding protein and changes in repeat-silencing modification H3K9me3 as an effect of CGGBP1 knockdown is not surprising. Importantly, this change was observed to occur very strongly at 879 locations in the genome which are CGGBP1-regulated CTCF-binding sites. These sites were identified in this study by applying stringent thresholds of H3K9me3 signal differences in the CTCF-binding site flanks in CT and KD. At these binding sites, CTCF maintains a certain pattern of H3K9me3 in the flanking regions that depends on CGGBP1. Considering these CTCF-binding sites as boundaries between contrasting histone modification patterns, CGGBP1 emerges as a regulator of chromatin barrier function of CTCF. By comparing CT with KD, we have identified new locations with CGGBP1-dependent gain and loss of barrier functions. This identification of the potential barrier elements is robust, as it is based on a three-step filtration: binding of CTCF on these sites, CGGBP1-dependent changes in CTCF occupancy, and changes in H3K9me3 but not necessarily in other modifications asymmetrically in the flanks of these sites. The repeat and motif contents in these sets of sequences show that upon CGGBP1 depletion, the binding of CTCF and associated chromatin barrier activity are more prominently detected at repeat-poor and motif-rich regions due to a repeat-to-motif shift of CTCF occupancy. The detection of the barrier activity of repeat-rich regions depends on an approach that includes repeats as bona fide binding sites for insulator proteins, such as CTCF. CGGBP1 forms complex with histone methyltransferase SUV39H2 [54] and a loss of regulation of SUV39H2 by CGGBP1 in KD could enable a selective change in H3K9me3 only and not other histone marks. Our analyses reveal that there are repeat elements that act as barrier elements through a CGGBP1–CTCF cross talk. Genome browsing of ENCODE datasets does show abundant CTCF binding at repeats. However, the regulation of this was unclear so far and our findings for the first time provide some explanations. Thus, the barrier function of most of the CTCF-bound repeats strongly depends on CGGBP1, unlike that of the repeat-free sequences. Regulation of CTCF binding by CGGBP1 could be important for repeat heterochromatinization and silencing. A disruption of repeat heterochromatinization could alter gene expression patterns. The CGGBP1-dependent CTCF-binding barrier elements do not appear to be short-range *cis* regulators of gene expression, as they are tens of kilobases away from nearest permissive TSSs. We attempted to assess the differential gene expression changes caused by CGGBP1 depletion in the flanks of these potential barrier elements by a targeted quantitative real-time polymerase chain reaction assay of candidate TSSs. However, due to the rare distribution of TSSs around these sequences and unpredictable effects of the neighboring sequences on the transcription of these TSSs, this approach was not conclusive (not shown). Still, a remarkable transcript-level recapitulation of the H3K9me3 asymmetry in the flanks of these barrier sequences was found through a non-candidate approach in which we used a published RNA-seq dataset to discover transcript-level asymmetries similar to the H3K9me3 asymmetries across the barrier elements. Our findings implicate the CGGBP1-dependent CTCF binding at L1 repeats in transcription regulation via a chromatin barrier function.

## Conclusion

We have discovered that CGGBP1 is a regulator of CTCF–DNA-binding pattern with a direct effect on a potential chromatin barrier like functioning of repeat-rich and motif-rich regions. Our results demonstrate that a functional outcome of the CTCF-binding preference on repeats or motifs determines the function of genomic sites as barriers between contrasting levels of H3K9me3. This pivotal function of CTCF at repeats depends on CGGBP1. CGGBP1 has evolved later than CTCF. Thus, CGGBP1 is not required for DNA binding of CTCF, but only acts as a fine adjuster of CTCF-binding pattern and through it the chromatin structure and function. The CGGBP1–CTCF cross talk is thus an essential part of functioning of CTCF.

## Materials and methods

### Cell culture and lentivirus transduction

Human dermal fibroblast (Sigma, passage 15–24), human juvenile foreskin fibroblast (Himedia, passage 5–30) and HEK-293T cells were grown in DMEM (AL007A) supplemented with 10% FBS. Control or CGGBP1-shRNA (targeting 4 different regions in CGGBP1 ORF) or CGGBP1-overexpression lentivirus constructs were obtained from Origene. The third-generation lenti-packaging plasmids: pRSV-Rev (12,253), pMDLg/pRRE (12,251) and pMD2.G (12,259) were obtained from Addgene. For lentiviral production, the packaging plasmids and lentiviral constructs were mixed in equimolar ratios and used for transfection. Transfection was performed using FuGene (Promega). A 1:10,000 dilution of 10 mg/ml polybrene stock (Sigma) was used for transducing HEK-293T cells. For stable transduction, control and CGGBP1 shRNA-transduced cells were selected with 10 μg/ml puromycin (Himedia).

### Immunofluorescence and antibody blocking assays

CGGBP1 and CTCF immunofluorescences were carried out in human juvenile foreskin fibroblast using standard protocol. Briefly, cells were fixed for 10 min in 3.7% formaldehyde solution (diluted in 1× PBS) followed by permeabilization with 1% Triton X-100 in PBS for 10 min. After permeabilization, cells were incubated with 10% FBS and 0.05% Triton X-100 in PBS for an hour. Subsequently, cells were incubated with primary antibodies (anti-CGGBP1 rabbit polyclonal and anti-CTCF mouse monoclonal or anti-CGGBP1 mouse monoclonal and anti-CTCF rabbit polyclonal) for 2 h at room temperature followed by incubation with secondary antibodies (anti-mouse Alexa fluor 594 and anti-rabbit Alexa fluor 488) for 2 h at room temperature. Samples were counterstained with Fluoroshield Mounting Medium (Ab104135) containing 4′,6-diamidino-2-phenylindole.

Antibody blocking assay was performed with a modification of the protocol described by Melnik et al. [25]. Cells were incubated with blocking antibody (anti-CGGBP1 rabbit polyclonal or anti-CTCF mouse monoclonal) for 1 h at room temperature, followed by co-incubation with detection antibodies. Cells were incubated with secondary antibodies for 2 h at room temperature. Samples were counterstained with Fluoroshield Mounting Medium (Ab104135) containing 4′,6-diamidino-2-phenylindole. Samples were examined using a Leica confocal microscope (magnification with 10× eyepiece and 40× objective). All the images were captured with confocal plane z-stack of 1.601 µ.

### Proximity ligation assays (PLA)

Duolink PLA kit was used to detect protein–protein interactions (Sigma Aldrich DUO92101). PLA was performed according to THE manufacturer’s protocol with additional negative controls. In brief, human juvenile fibroblast were fixed with 4% formaldehyde solution at 37 °C for 10 min, followed by permeabilization with 1% Triton X-100 in PBS for 10 min at room temperature. Cells were washed with PBS three times followed by incubation in blocking buffer at 37 °C for 1 h. Samples were subsequently incubated with primary antibody pairs (two pairs of specific antibodies, one pair each of a specific and a non-specific antibody) or only blocking buffer at room temperature for 2 h. Samples were incubated with oligo probe-conjugated secondary antibodies and ligation and amplification were carried out to produce rolling circle PCR product. The amplified products were detected by hybridization with Texas Red-labeled oligonucleotides and the samples were counterstained with Fluoroshield Mounting Medium (Ab104135) containing 4′,6-diamidino-2-phenylindole. Samples were examined using a Leica confocal microscope (10× eyepiece and 40× objective).

### Co-immunoprecipitation and immunoblotting assay

Cell lysates were cleared by centrifugation and subjected to pre-clearance by incubating with a non-specific antibody/IgG and Protein G Sepharose beads (GE 17-0618-01). Pre-cleared cell lysates were separately incubated with CGGBP1 or CTCF antibodies overnight, followed by incubation with Protein G Sepharose beads for 2 h and 4 °C. Protein-bound Sepharose beads were washed four times with PBS. Beads-bound fractions were subjected to elution by boiling in SDS-Laemmli buffer followed by SDS PAGE and immunoblotting with the indicated antibodies. Raw data are presented in Additional file 3: Appendix I.

### Co-immunoprecipitation followed by DNaseI digestion assay

The cytoplasmic and nuclear fractions were separated from HEK-293T cells by REAP protocol [55]. The cytoplasmic fractions were pre-cleared by incubating with Protein G Sepharose beads, followed by immunoprecipitation with CGGBP1 and CTCF separately. Nuclear extracts were pre-cleared by incubating with Protein G Sepharose beads. Pre-cleared nuclear extracts were incubated with primary antibodies overnight, followed by incubation with Protein G Sepharose beads for 2 h at 4 °C. For DNaseI digestion, the immunoprecipitated protein-bound Sepharose beads were washed four times with PBS and suspended in the DNaseI digestion buffer containing DNaseI (6 Units of M0303S, NEB). The samples were incubated for 10 min at room temperature and centrifuged at low speed to separate two fractions: beads–antibody-bound proteins (pellet) and proteins released by DNaseI digestion (supernatant). The separated fractions were subjected to SDS PAGE followed by western blotting. Raw data are presented in Additional file 3: Appendix I.

### CTCF ChIP sequencing

CTCF ChIP was performed by using MAGnifyTM Chromatin Immunoprecipitation System kit (Invitrogen 49-2024) with minor modifications to the protocol. HEK-293T cells were transduced with lentiviruses expressing non-targeting shRNA, CGGBP1-targeting shRNA or CGGBP1-FLAG. The shRNA-transduced cells were selected with puromycin (10 µg/ml) for 7 days. Approximately, 200 millions cells were cross-linked by 4% formaldehyde solution at 37 °C for 10 min followed by quenching with 125 mM glycine. Cells were washed with PBS twice and harvested using scrapers on ice. Cross-linked cells were resuspended in protease inhibitor containing SDS lysis buffer and sonicated using a Diagenode bioruptor for 30 cycles at 30 s on, followed by 30 s off. Sonication was standardized to yield fragments with mean length 150 ± 50 bp. Sonicated lysates were cleared by centrifugation at 16,000 rcf for 5 min at 4 °C. A 33 ul aliquot of each lysate was reserved as input. CTCF ChIP was performed by using 150 µl of sonicated lysates. Sonicated lysates were incubated overnight at 4 °C with antibody-conjugated beads. Beads were washed thrice with IP wash buffer 1, followed by washing with IP wash buffer two times. Cross-links were reversed in reverse cross-linking buffer for 15 min at 55 °C, followed by Proteinase K digestion at 65 °C for 15 min. Reverse cross-linked DNA was purified by DNA purification magnetic beads and used for library preparation and sequencing and described further below.

### Histone ChIP-sequencing

All the steps of ChIP for H3K4me3, H3K9me3 and H3K27me3 up to sonication of lysates of fixed cells were performed exactly as described above for CTCF ChIP. Sonicated lysates were pre-cleared by incubating with non-specific antibody/IgG and protein G Sepharose beads. The pre-cleared lysates were incubated overnight at 4 °C with antibody-conjugated beads. Beads were washed with the following buffers: low-salt IP wash buffer (0.1% SDS,1% Triton X-100, 2 mM EDTA, 20 mM Tris–HCl and 150 mM NaCl), high-salt IP wash buffer (0.1% SDS,1% Triton X-100, 2 mM EDTA, 20 mM Tris–HCl and 500 mM NaCl), LiCl IP wash buffer (0.25 M LiCl, 1% IGEPAL, 1% Na deoxycholate, 1 mM EDTA and 10 mM Tris–HCl). Beads were finally washed with TE buffer twice (10 mM Tris–HCl and 1 mM EDTA). Beads-bound DNA was eluted in elution buffer (1% SDS and 0.1 M NaHCO_3_). Cross-links were reversed by heating with 20 µl of 5 M NaCl at 65 °C for 4 h, followed by Proteinase K digestion. DNA was subsequently purified from each sample by using the DNA purification kit (Promega A1460) and used for library preparation and sequencing.

### Ion Torrent S5 library preparation and sequencing

The Ion XpressTM Plus Fragment Library Kit (Cat. no. 4471269) was used for library preparation from the above-mentioned ChIP DNA samples. All the steps were performed as per manufacturer’s instructions for sequencing on Ion Torrent S5 platform without any barcoding. Briefly, the DNA were subjected to end repair. The purified end-repaired DNA was ligated to Ion Torrent platform-compatible adapters. Nick repair was carried out to ensure the linking of the barcode adapters and DNA inserts on both strands. The library was then amplified by PCR (number of cycles restricted to less than 18). AMPure XP beads were used to purify the PCR products (two rounds). The size-selected fragments were used for downstream clonal amplification on ion sphere particles in an emulsion PCR. The prepared samples were sequenced on the Ion Proton S5 sequencer.

### Quality control and mapping of the ChIP-sequencing reads

Unpaired sequencing reads were trimmed and subjected to proprietary quality filtration for the Ion Torrent platform (minimum quality threshold 20). Reads with sequence length ranging from 80 to 300 base were used for further analysis. Reads were mapped using bowtie2 against the repeat-masked (hg38.fa.masked) or repeat-unmasked (hg38.fa) human genome as described. Reads were mapped end-to-end with default options. A comparison of alignments to masked and unmasked hg38 is also presented in Additional file 3: Appendix II.

### Peak calling

Peaks were called by using MACS2 on repeat-masked reads at default settings for statistical significance (*p* value < 0.001). For repeat-unmasked reads, peaks were called by using MACS2 with –*min*-*length 200* option with statistical significance (*p* value < 0.005). Representative peaks for CTCF and H3K9me3, and read distributions in them are presented in Additional file 3: Appendix III. Multiple genome views of repeat-masked alignments-derived peaks for our CTCF ChIP-seq and its comparison with some ENCODE datasets are presented in Additional file 3: Appendix IV.

### Analyses of genomic coordinates and sequences of regions

Sequences of bed coordinates were extracted from hg38 (repeat-masked or unmasked as described) using bedtools getfasta tool. To isolate exclusive and overlapping CTCF peaks for datasets, bedtools intersect tool was used. To identify the number of overlapping ChIP-seq reads in any bed coordinate, bedtools coverage tool was used with the fraction of overlap option. The closest distance between two different sets of bed coordinates were obtained using bedtools closest tool. A perl script Fasta-splitter was used to randomly sample sequence from fasta files.

### Repeat content analyses

The repeat-masked and unmasked hg38 genome were used from UCSC genome browser. Sequences were repeat-masked using a locally installed version of RepeatMasker. The repeat search engine used was RMBlast (NCBI) and the repeat database used was obtained from Repbase.

### Motif finding

De novo motif search was done using locally installed versions of MEME (version 5.0.3) suite tools meme and dreme. Suite tool fimo was used to find predicted motifs in sequence datasets. The motif search was performed using default options with -k value range from 12 to 15. Suite tool Tomtom was used to search for motif-corresponding transcription factors at HoCoMoCo database for transcription factor motifs. Motif-shuffle-columns was used to generate position-shuffled motifs from the short L1-matching sequences. The search for the 2x CTCF motifs was performed in repeat-free motif-containing peaks using the CTCF motif and peak sequences in FIMO without any inter-motif distance threshold. The motifs counts reported are combined counts from the two strands, including overlapping occurrences. The motifs discovered in the repeat-masked peaks corresponded mostly to CTCF-binding sites. The motifs found in peaks which were negative for the canonical CTCF-binding motifs and were rich in LINE-1 repeats are shown in Additional file 3: Appendix V.

### Plotting of signals in genomic coordinates

CTCF and histone modification ChIP-seq signals were plotted along genomic coordinates using deepTools. Bam to BigWig conversions were done using bamcoverage tool. Matrices were generated by using computeMatrix in deepTools. The plots on these matrices were generated by using plotHeatmap (for heatmap) and plotProfile (for summary plot) functions.

### Statistics and graphs

Statistical tests were performed using Prism 8 (GraphPad) on numerical data generated from the aforementioned tools including OpenOffice Spreadsheet. R-scripts were used to plot the distribution of CTCF reads in 0.5 kb bins from 1 million bins randomly selected from the whole genome (ggplot2), PCA analysis and hierarchical clustering (DiffBind) and correlation analysis of CTCF ChIP-seq datasets. Visualization of genomic features was carried out by the GUI version of locally installed Integrated Genome Viewer, where *Y*-axis ranges are derived by using the “Autoscale” function in IGV. All microscopy images were analyzed by ImageJ. ImageJ plugin Coloc 2 was used to Manders colocalization analysis. A Fisher's exact test of the AUC distributions is shown in Additional file 3: Appendix VI.

### Antibodies

Antibodies used in this study are as follows: CGGBP1 western blot (Proteintech 10716-1-AP), CGGBP1 IP (Proteintech 10716-1-AP; Santacruz SC-376482), CTCF western blot (SC-271514), CTCF IP (Santa Cruz SC-28198 and SC-271514), CTCF ChIP (Santa Cruz SC-28198 and SC-271514), H3K4me3 ChIP (ABCAM ab8580), H3K9me3 ChIP (SantaCruz SC-130356) and H3K27me3 ChIP (ABCAM ab6002), IgG ChIP preincubation (FBS from Himedia), GAPDH (NB300-328SS), Mouse and Rabbit IgG in PLA (Invitrogen).

### Publicly available data usage

The following publicly available datasets were used in this study: ChIP-seq datasets from ENCODE for proximity analysis (ENCFF567GON, ENCFF757KYL, ENCFF510QXG, ENCFF712LFQ, ENCFF687IUD, ENCFF420KMT, ENCFF217ZMF, ENCFF180BYN, ENCFF987YIJ, ENCFF002CVC, ENCFF351VGZ, ENCFF002CVE, ENCFF082IQD, ENCFF474PPT, ENCFF002CVF, ENCFF560WFS, ENCFF895JAW, ENCFF139EBY, ENCFF380ZXB and ENCFF938BOJ). Datasets used for ChIP-seq comparisons are stated in the results section. UCSC CTCF Binding Sites (https://genome-test.gi.ucsc.edu/cgi-bin/hgTables?command=start), UCSC Regulatory Elements (available through UCSC table Browser). Human genome hg38 was downloaded from UCSC. UCSC LiftOver tool was used to convert genomic coordinates between different versions of the human genome. CGGBP1 ChIP-seq datasets were obtained from NCBI GEO Datasets (GSE53571).

### Sequence data availability

The sequence data described in this work are available through the NCBI Gene Expression Omnibus Datasets accession number GSE129548.

## Supplementary information

**Additional file 1:** The supplementary tables with captions are presented in Additional file 1.

**Additional file 2: Figure S1.** (A) Human juvenile fibroblasts co-immunostained for CGGBP1 (Green) and CTCF (Red). Nuclei were counterstained with DAPI (blue). Mean fluorescence intensities for CGGBP1 (green) and CTCF (red) were normalized along the line-marked segment and plotted using ImageJ. Normalized signals along the line segment drawn through a midbody shows colocalization of CGGBP1 and CTCF. (B) Human juvenile fibroblasts co-immunostained for CGGBP1 (red) and CTCF (Green). Nuclei were counterstained with DAPI (blue). All images were captured with confocal plane of 1.601 µm. **Figure S2.** PLA (red foci) confirms CTCF-CGGBP1 interaction in situ. Nuclei were stained with DAPI (blue). CGGBP1-CTCF interaction was stronger in the nuclei than in cytoplasm (inset of mouse anti-CGGBP1:rabbit anti-CTCF sample). No significant interaction was observed in IgG and no-primary antibody negative controls (inset of no-primary antibody sample). All images were captured with confocal plane of 1.601 µm. **Figure S3.** Cytoplasmic and nuclear fractions were separated from HEK293T cells by REAP protocol. The upper panel shows immunoblot results for cytoplasmic marker GAPDH. The middle and the lower panels show immunoblot results for a nuclear protein Histone H3 using two different antibodies (H3K4me3 and H3K27me3). Equal volumes of cytoplasmic and nuclear fraction lysates were run in the lanes. **Figure S4.** The closest distance between starved RM CGGBP1 peak midpoint and transcription factor peak midpoint was determined by using bedtools closest. Frequency distribution of closest distances was plotted in bin of 0.5 kb for starved RM CGGBP1 peaks (A) and stimulated RM CGGBP1 peaks (B). **Figure S5.** HEK293T cells were transduced with control shRNA lentivirus, CGGBP1-shRNA lentivirus and CGGBP1-overexpression lentivirus, respectively. The upper panel shows immunoblot results for CGGBP1 and lower panel shows same for GAPDH loading control. **Figure S6.** The distribution of CTCF reads for repeat-masked peaks was plotted for CT, KD and OE samples. CT reads at repeat-masked CTCF CT peaks was plotted for 1 kb flanks in bin size of 10 (A). The distribution of published CTCF reads in prostate epithelial cells (ENCFF098DGZ) (B), A549 (ENCFF9810JS) (C) and HEK293 (ENCFF183AAP) (D) was also plotted at CTCF CT peaks. (E to H) The distribution of CTCF KD reads at repeat-masked CTCF KD peaks was plotted for 1 kb flanks in bin size of 10 (E). The distribution of published CTCF reads in prostate epithelial cells (ENCFF098DGZ) (F), A549 (ENCFF9810JS) (G) and HEK293 (ENCFF183AAP) (H) was also plotted at CTCF KD peaks. (I to L) The distribution of CTCF OE reads at repeat-masked CTCF OE peaks was plotted for 1 kb flanks in bin size of 10 (I). The distribution of published CTCF reads in prostate epithelial cells (ENCFF098DGZ) (J), A549 (ENCFF9810JS) (K) and HEK293 (ENCFF183AAP) (L) was also plotted at CTCF OE peaks. **Figure S7.** From RM CGGBP1 peaks with tag count more than 10, peaks having summits in central one-third region of peak length were filtered out for analysis. Sequences of these summit regions of the selected peaks (start = (peak start + 0.4 × peak length) and end = (peak start + 0.667 × peak length)) were fetched from repeat-masked hg38 and subjected to *de novo* motif search using DREME (*minK 8*). Despite repeat-masking these RM CGGBP1 peaks were centrally enriched with motifs with sequences that correspond to subsequences of Alu-SINEs (A) and L1-LINEs (B). The occurrences of these repeat-derived motifs were observed for starved (locations in blue) as well as stimulated (locations in red) peaks. Some motifs occur more than once on the transposon consensus sequences. **Figure S8.** Distribution of alignment scores of reads was plotted for mapping on hg38 masked and unmasked genome. End-to-end alignment score is represented on *X*-axis and percentage of aligned reads on the *Y*-axis. **Figure S9.** CTCF reads (CT, KD and OE) distribution on UCSC LINEs was plotted. LINEs coordinates were scaled to 0.3 kb and signal was plotted for 1 kb flanks by using *kmeans* clustering option. **Figure S10.** Repeat content analysis of reads prior to mapping, post-mapping and CTCF peaks. No significant difference between CT and KD was observed for presence of LINEs or SINEs in reads subjected to repeat content analysis prior and post-mapping. However, a significant difference is observed in LINE content between CT and KD on the peaks. **Figure S11.** The distribution of CTCF reads for repeat-unmasked peaks was plotted for CT, KD and OE samples. CT reads at repeat-unmasked CTCF CT peaks was plotted for 1 kb flanks in bin size of 10 (A). The distribution of published CTCF reads in prostate epithelial cells (ENCFF098DGZ) (B), A549 (ENCFF9810JS) (C) and HEK293 (ENCFF183AAP) (D) was also plotted at CTCF CT peaks. (E to H) The distribution of CTCF KD reads at unmasked CTCF KD peaks was plotted for 1 kb flanks in bin size of 10 (E). The distribution of published CTCF reads in prostate epithelial cells (ENCFF098DGZ) (F), A549 (ENCFF9810JS) (G) and HEK293 (ENCFF183AAP) (H) was also plotted at CTCF KD peaks. (I to L) The distribution of CTCF OE reads at unmasked CTCF OE peaks was plotted for 1 kb flanks in bin size of 10 (I). The distribution of published CTCF reads in prostate epithelial cells (ENCFF098DGZ) (J), A549 (ENCFF9810JS) (K) and HEK293 (ENCFF183AAP) (L) was also plotted at CTCF OE peaks. **Figure S12.** (A) The distribution of repeat-masked (top) and repeat-unmasked (bottom) CTCF reads in randomly picked 0.5 kb long 1 million genomic regions for CT, KD and OE samples. CT shows a bipolar distribution pattern with preponderance of read-free and highly read-rich region with a paucity of regions with moderate read density strongly when repeats are unmasked (bottom) as compared to repeat-masked (top). On the contrary, including the repeats shifts the KD and OE read distribution patterns toward the center with a majority of moderate read density regions. (B) Principal Component Analysis (PCA) to find the patterns of differences between CT, KD, OE and input (upper panel) shows that all the three ChIP samples differ from input in different ways. The specificity of CTCF ChIP causes a difference from input that is majorly PC1 for CT, majorly PC2 for OE and a mix of PC1 and PC2 for KD. **Figure S13.** Pie chart represents CTCF peaks segregation according to presence or absence of CTCF motif or LINEs in peaks. Pie-charts in top for CT (blue), middle for KD (red) and bottom for OE (green) peaks. The panel on the right represents the CTCF read distribution along with standard deviation plotted in immediate flanks of the Motif-negative LINE-positive CTCF peaks (top) and Motif-positive LINE-negative CTCF peaks (bottom) for CT (blue), KD (red) and OE (green). **Figure S14.** Distribution of CTCF reads at replication origin was plotted for 5 kb flanks in bin size of 10 (A). Distribution of CTCF reads at enhancers (UCSC Regulation datasets) was plotted for 5 kb flanks in bin size of 10 (B). **Figure S15.** Distribution of histone modification reads for CT and KD samples was plotted in 1mb upstream and downstream of LAD boundary. Histone modification reads counts in bin size of 1 kb was plotted for CT (blue) and KD (red) peaks at LAD start sites (A) and LAD end sites (B). **Figure S16.** Distribution of CTCF and histone modification reads at UCSC CTCF binding sites was plotted for 5 kb flanks in bin size of 10 (A). Variation of CTCF and histone reads in bin size 10 at UCSC CTCF-binding sites was shown as difference from mean for CT and KD sample (B). **Figure S17.** Difference in histone modification read occupancy in upstream and downstream 10 kb flanks of the all exclusive peaks were compared between CT and KD. Reads coverage count for 10 kb upstream and downstream was converted to logarithmic scale (log base = 2). Log2 fold change (M = Upstream–Downstream) was plotted against average read count for CT (A) and KD (B). **Figure S18.** Δ*M* value for CT–KD exclusive peaks were calculated for the H3K4me3 reads. Those peaks with significantly different (Δ*M* value < − 2 to >+2) H3K4me3 profile in CT and KD are represented as blue for CT (M-CT diff) and red for KD (M-KD diff), whereas those peaks which H3K4me3 profile did not alter significantly in KD (Δ*M* value ranges from − 2 to + 2) are highlighted as green for CT (M-CT indiff) and yellow for KD (M-KD indiff) (A). Similarly, Δ*M* value was calculated for KD–CT exclusive peaks and those peaks which showed substantial changes (Δ*M* value < − 2 and >+ 2) in H3K4me3 profile in KD are represented as red for KD (M-KD diff) and blue for CT (M-CT diff). Peaks which H3K4me3 profile did not change substantially by CGGBP1 depletion are shown as yellow for KD (M-KD indiff) and green for CT (M-CT indiff) (B). (C and D) Similarly, Δ*M* value for CT–KD exclusive peaks were calculated for the H3K27me3 reads. Those peaks with significantly different (Δ*M* value < − 1 to >+ 1) H3K27me3 profile inCT and KD are represented as blue for CT (M-CT diff) and red for KD (M-KD diff), whereas those peaks which H3K27me3 profile did not alter significantly in KD (Δ*M* value ranges from − 1 to + 1) are highlighted as green for CT (M-CT indiff) and yellow for KD (M-KD indiff) (C). Similarly, Δ*M* value was calculated for KD–CT exclusive peaks and those peaks which showed substantial changes (Δ*M* value < − 1 and >+ 1) in H3K27me3 profile in KD are represented as red for KD (M-KD diff) and blue for CT (M-CT diff). Peaks which H3K27me3 profile did not change substantially by CGGBP1 depletion are shown as yellow for KD (M-KD indiff) and green for CT (M-CT indiff) (D). **Figure S19.** The closest distance between permissive TSSs and CGGBP1-dependent CTCF-binding sites was obtained by bedtools closest. Distribution of distance between CGGBP1-dependent CTCF-binding sites and permissive was plotted.

**Additional file 3:** Appendices I to VI in the Additional file 3 show the raw data and supporting information for the various experiments and analysis steps.

## Data Availability

Sequence data have been deposited at NCBI-GEO (GSE129548).
