# Supplementary material for: CGGBP1 regulates CTCF occupancy at repeats
Source: Epigenetics Chromatin. 2019 Sep 23;12:57. doi: 10.1186/s13072-019-0305-6 (PMC6757366; doi:10.1186/s13072-019-0305-6)
Supplement: Supplementary file 1 — Additional file 1: The supplementary tables with captions are presented in Additional file 1. [file 13072_2019_305_MOESM1_ESM.pdf]

Sample	Coincubation	1 = CTCF<br>2 = CGGBP1	1 = CGGBP1<br>2 = CTCF
**Nuclear**	Ch1 = CTCF<br>Ch2 = CGGBP1	Ch1 = CTCF<br>Ch2 = CGGBP1	Ch1 = CTCF<br>Ch2 = CGGBP1
% zero-zero pixels:	63.37	81.62	79.77
% saturated ch1 pixels	0.01	0.08	0.01
% saturated ch2 pixels	0.04	0.03	0.03
Threshold regression:	Costes	Costes	Costes
Pearson's R value (no threshold):	0.37	0.48	0.26
Pearson's R value (above threshold):	0.12	0.25	-0.17
Spearman's rank correlation value:	0.26220762	0.26932225	0.16809934
Manders' tM1 (Above autothreshold of Ch2):	0.472	0.523	0.227
Manders' tM2 (Above autothreshold of Ch1):	0.568	0.404	0.284
Kendall's Tau-b rank correlation value:	0.2429	0.2579	0.1607
**Cytoplasmic + Nuclear**	Ch1 = CTCF<br>Ch2 = CGGBP1	Ch1 = CTCF<br>Ch2 = CGGBP1	Ch1 = CTCF<br>Ch2 = CGGBP1
% zero-zero pixels:	63.37	81.62	79.77
% saturated ch1 pixels	0.01	0.08	0.01
% saturated ch2 pixels	0.04	0.03	0.03
Threshold regression:	Costes	Costes	Costes
Pearson's R value (no threshold):	0.37	0.48	0.26
Pearson's R value (above threshold):	0.12	0.25	-0.17
Spearman's rank correlation value:	0.26220762	0.26932225	0.16809934
Manders' tM1 (Above autothreshold of Ch2):	0.472	0.523	0.227
Manders' tM2 (Above autothreshold of Ch1):	0.568	0.404	0.284
Kendall's Tau-b rank correlation value:	0.2429	0.2579	0.1607

Table S1: Table shows the statistics of Mander’s co-localization analysis by using ImageJ plugin Coloc 2.

Sample Name	Total Reads	Mapped Reads	Unmapped Reads	Percentage of mapped reads	Percentage of unmapped reads
**RM CTCF CT**	99086307	33436004	65650303	33.74	66.25
**RM CTCF KD**	76777977	31636108	45141869	41.20	58.79
**RM CTCF OE**	74091715	28146793	45944922	37.98	62.01

Table S2: Table shows number and percentage of mapped and unmapped CTCF reads to repeat-masked human genome (hg38).

Sample Name	Number of RM CTCF peaks	Number of CTCF motifs in CTCF peak sequences	Average number of motifs/peak
**RM CTCF CT**	26635	15187	0.57
**RM CTCF KD**	24418	23563	0.96
**RM CTCF OE**	21071	18983	0.90

Table S3: Repeat-masked (RM) CTCF reads were used to call peaks. The table shows number of repeat-masked CTCF peaks and CTCF motifs count in peak sequences.

Sample Name	Number of RM CTCF peaks	Motif count (L1-matching)	Motif count (L1-matching and position shuffled)
**RM CTCF CT**	26635	18986	4483
**RM CTCF KD**	24418	36253	13740
**RM CTCF OE**	21071	34391	11143
**RM starved CGGBP1 narrow peaks**	1135	24772	452
**RM stimulated CGGBP1 narrow peaks**	1438	40693	680

Table S4: Table shows number of repeat-masked CTCF peaks, repeat-masked CGGBP1 narrow peaks. Number of L1-matching motifs and position shuffled L1-matching motifs in peak sequences are mentioned.

Sample Name	Total Reads	Mapped Reads	Unmapped Reads	Percentage of mapped reads	Percentage of unmapped reads
**CTCF CT**	99086307	79778533	19307774	80.51	19.49
**CTCF KD**	76777977	74389175	2388802	96.89	3.11
**CTCF OE**	74091715	67600036	6491679	91.24	8.76
**RM CTCF CT**	99086307	33436004	65650303	33.74	66.25
**RM CTCF KD**	76777977	31636108	45141869	41.20	58.79
**RM CTCF OE**	74091715	28146793	45944922	37.98	62.01

Table S5: Table shows number and percentage of mapped and unmapped CTCF reads to repeat-unmasked human genome (hg38)

Pre-alignment read ID	Bowtie2 alignment coordinates	NCBI Blast mapped coordinates
CU6T3:01332:11636	chr1:177553160-177553334	chr1:177553161-177553334
CU6T3:01334:11602	chr12:79281006-79281097	chr12:79281007-79281097
CU6T3:01335:11596	chr2:145125281-145125426	chr2:145125282-145125426
CU6T3:01341:11641	chr11:14199565-14199679	chr11:14199566-14199679
CU6T3:01346:11619	chr4:73171803-73171940	chr4:73171804-73171940
CU6T3:01346:11632	chr3:174797791-174797961	chr3:174797792-174797961
CU6T3:01348:11597	chr14:19066723-19066845	chr14:19066724-19066845
CU6T3:01353:11607	chrX:28129661-28129812	chrX:28129662-28129812
CU6T3:01354:11623	chr8:88860668-88860766	chr8:88860669-88860766
CU6T3:01357:11637	chr21:6515452-6515577	chr21:6515454-6515577

Table S6: Table shows reads entirely aligning to LINE1, bowtie2 alignment coordinates and NCBI Blast mapped coordinates.

	CTCF CT	CTCF KD	CTCF OE
**SINEs**	6.1	3.53	3.11
ALUs	4.85	2.19	1.64
MIRs	1.23	1.3	1.43
**LINEs**	19.14	9.65	10.46
LINE1	17.79	8.32	8.99
LINE2	1.17	1.15	1.27
L3/CR1	0.13	0.14	0.14
**LTR elements**	4.65	4.99	5.1
ERVL	1.04	1.4	1.41
ERVL-MaLRs	1.33	1.1	1.36
ERV_classI	1.46	1.48	1.42
ERV_classII	0.68	0.82	0.73
**DNA elements**	2.28	2.4	2.35
hAT-Charlie	1.14	1.33	1.24
TcMar-Tigger	0.61	0.55	0.61
**Unclassified**	0.38	0.1	0.09
**Total interspersed repeats**	32.54	20.66	21.11
**Small RNA**	0.16	0.22	0.24
**Satellites**	11.48	11.49	14.4
**Simple repeats**	0	0	0
**Low complexity**	0	0	0

Table S7: Repeat content in CTCF peaks for CT, KD and OE samples.

CT motif occurrences Total Number of Peaks = 42978		KD motif occurrences Total Number of Peaks = 47632		OE motif occurrences Total Number of Peaks = 47216	
**Motif**	Ocuurances	Motif	Ocuurances	Motif	Ocuurances
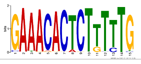	3662	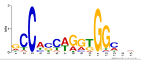	36405	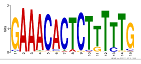	5521
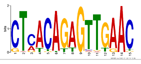	4384	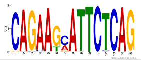	3972	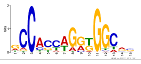	21767
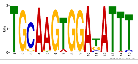	3680	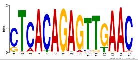	4169	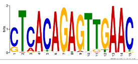	4913
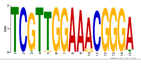	2866	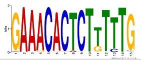	3516	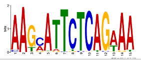	5500
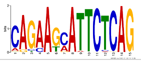	4287	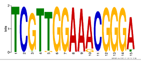	3094	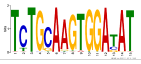	5019
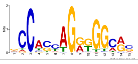	12807	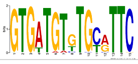	3238	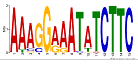	3787
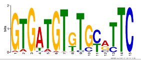	4399	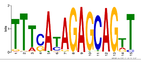	3896	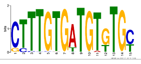	4440
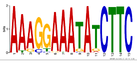	2643	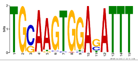	2313	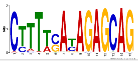	4858
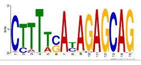	3827	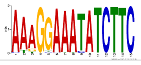	2739	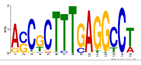	3380
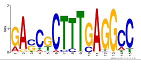	3046	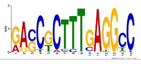	2785	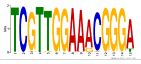	2703

Table S8: Table shows top 10 motif occurrences discovered in CTCF peaks for CT, KD and OE.

	CT-KD peaks with motifs	CT-OE peaks with motifs	KD-CT peaks with motifs	KD-OE peaks with motifs	OE-CT peaks with motifs	OE-KD peaks with motifs
**SINEs**	8.76	6.52	3.36	3.65	2.77	3.28
ALUs	7.12	5.28	1.83	2.16	1.34	1.29
MIRs	1.59	1.21	1.47	1.43	1.38	1.93
**LINEs**	8.08	7.02	5.37	5.96	5.29	8.23
LINE1	6.54	5.61	3.87	4.43	3.81	6.07
LINE2	1.32	1.26	1.33	1.35	1.27	1.69
L3/CR1	0.22	0.14	0.14	0.16	0.16	0.32
**LTR elements**	7.93	7.93	7.24	8.66	6.57	8.53
ERVL	1.6	1.78	1.72	1.96	1.81	2.25
ERVL-MaLRs	2.51	2.02	1.81	2.14	1.67	2.61
ERV_classI	2.61	2.31	2.18	2.73	1.82	2.62
ERV_classII	1.14	1.6	1.26	1.51	0.97	0.76
**DNA elements**	2.47	2.37	2.64	2.54	3.02	3.11
hAT-Charlie	1.68	1.53	1.47	1.34	1.86	1.99
TcMar-Tigger	0.38	0.37	0.55	0.59	0.51	0.42
**Unclassified**	0.07	0.07	0.08	0.11	0.06	0.07
**Total interspersed repeats**	27.3	23.91	18.7	20.92	17.72	23.22
**Small RNA**	0.11	0.1	0.04	0.04	0.05	0.04
**Satellites**	12.3	10.97	2.95	5.64	2.28	6.98
**Simple repeats**	0	0	0	0	0	0
**Low complexity**	0	0	0	0	0	0

Table S9: Repeat content in exclusive CTCF peaks with CTCF motif for CT, KD and OE.

	CT-KD peaks no motifs	CT-OE peaks no motifs	KD-CT peaks no motifs	KD-OE peaks no motifs	OE-CT peaks no motifs	OE-KD peaks no motifs
**SINEs**	9.24	9.18	4.55	4.97	4.05	4.25
ALUs	7.74	7.71	3.05	3.57	2.14	2.27
MIRs	1.46	1.45	1.46	1.38	1.89	1.96
**LINEs**	30.95	30.67	14.77	19.75	15.63	19.5
LINE1	29.2	28.95	13.04	17.98	13.56	17.33
LINE2	1.5	1.49	1.44	1.47	1.78	1.85
L3/CR1	0.17	0.17	0.22	0.23	0.2	0.22
**LTR elements**	5.27	5.25	5.15	5.1	6.12	6.11
ERVL	0.83	0.84	1.53	1.27	1.48	1.28
ERVL-MaLRs	2.04	2.01	1.42	1.52	2.2	2.39
ERV_classI	1.83	1.83	1.67	1.73	1.83	1.85
ERV_classII	0.44	0.43	0.4	0.48	0.45	0.45
**DNA elements**	2.75	2.73	3.05	2.92	2.74	2.8
hAT-Charlie	1.2	1.18	1.6	1.43	1.18	1.17
TcMar-Tigger	0.93	0.9	0.88	0.93	1.04	1.15
**Unclassified**	0.67	0.69	0.1	0.15	0.1	0.12
**Total interspersed repeats**	48.87	48.52	27.62	32.9	28.64	32.78
**Small RNA**	0.05	0.03	0.21	0.07	0.2	0.09
**Satellites**	7.89	7.84	13.46	12.65	18.92	18.61
**Simple repeats**	0	0	0	0	0	0
**Low complexity**	0	0	0	0	0	0

Table S10: Repeat content in exclusive CTCF peaks without CTCF motif for CT, KD and OE.

Sample Name	Number of CTCF peaks	Number of L1-matching motifs in peaks	Motif positive peaks	Percentage peaks with L1-matching motifs	Average number of motifs/peak
**CTCF CT**	42978	96178	28798	67.01	3.34
**CTCF KD**	47632	93417	31525	66.19	2.96
**CTCF OE**	47216	103395	30450	64.49	3.40

Table S11: Table shows the number and the percentage of peaks with L1-matching motifs for CTCF peaks in CT, KD and OE.

	CT	KD
STEP 1: No. of TADs showing > 2 folds change in CTCF signal	CTCF signal on 1128 TADs in CT is reduced by CGGBP1 depletion in KD	CTCF signal on 390 TADs in CT is enhanced by CGGBP1 depletion in KD
STEP 2: No. of CTCF peaks found in the TADs that were identified in step 1.	2929	129
STEP 3: Paired T-test p-values for RNA-seq read signal 10kb upstream and downstream of the peaks identified in step 2.	RNA-seq (including no signal regions; n = 2926): 0.0437<br>RNA-seq (excluding no signal regions; n = 1748): 0.0437	RNA-seq (including no signal regions; n =129): 0.6856<br>RNA-seq (excluding no signal regions; n = 97): 0.6856

Table S12: Stepwise details of TADs and RNA-seq analysis from (Zuin et al. 2014) in CT and KD datasets. Table shows number of TADs showing differential CTCF occupancy upon CGGBP1 depletion, number of CTCF peaks called in such TADs and p-value of paired t-test to calculate covariance between cumulative signal for RNA level in 10kb upstream and downstream of CTCF binding sites at TADs.

Sample Name	Total Reads	Mapped Reads	Unmapped Reads	Percentage of mapped reads	Percentage of unmapped reads
**H3K4me3 CT**	110040103	100149169	9890934	91.01151877	8.988481227
**H3K4me3 KD**	93091534	90279250	2812284	96.97901208	3.020987924
**H3K9me3 CT**	82117627	69952262	12165365	85.18544015	14.81455985
**H3K9me3 KD**	80525298	61677565	18847733	76.59402266	23.40597734
**H3K27me3 CT**	95003513	83135564	11867962	87.50787037	12.49212963
**H3K27me3 KD**	66148370	63752690	2395680	96.37832346	3.621676543

Table S13: Table shows number and percentage of mapped and unmapped histone modification ChIP-seq reads to repeat-unmasked human genome (hg38).

Locus	Coordinates of CTCF-binding sites	Cumulative H3K4me3 signal in 10kb region				Cumulative H3K9me3 signal in 10kb region			
		Control		CGGBP1 knockdown		Control		CGGBP1 knockdown	
		Up	Down	Up	Down	Up	Down	Up	Down
H19-ICR	chr11:1990351-1990600	14893	32316	22608	39350	7943	14072	13300	8100
H19-ICR	chr11:1995351-1995751	20048	52534	29500	73650	3285	18565	16550	6846
H19-ICR	chr11:1999500-2000300	30286	39296	37500	58350	11424	10738	11100	6250
Beta globin LCR	chr11:5319500-5319650	38957	37773	31600	33250	49766	73252	58450	30750
Beta globin LCR	chr11:5331600-5331800	34051	32443	35900	30400	58984	44863	36000	29700
Beta globin LCR	chr11:5291300-5291600	43060	33416	37200	41350	32605	53051	38850	48900

Table S14: Table shows cumulative signal of histone modification in 10kb upstream and downstream of CTCF-binding sites present in H19 ICR and beta globin LCR.

	Loss of CTCF binding	Gain of CTCF binding
**SINEs**	9.54	2.34
ALUs	8.02	1.54
MIRs	1.52	0.81
**LINEs**	35.3	10.47
LINE1	34.38	9.36
LINE2	0.78	1.11
L3/CR1	0.09	0
**LTR elements**	5.9	6.81
ERVL	0.81	0.95
ERVL-MaLRs	1.8	1.7
ERV_classI	2.39	2.52
ERV_classII	0.78	1.31
**DNA elements**	2.9	1.77
hAT-Charlie	1.48	1.29
TcMar-Tigger	0.86	0.31
**Unclassified**	0.44	0
**Total interspersed repeats**	54.08	21.4
**Small RNA**	0.04	0
**Satellites**	1.35	4.75
**Simple repeats**	0	0
**Low complexity**	0	0

Table S15: Repeat content in exclusive CTCF peaks with differential H3K9me3 signal in flanks undergoing gain or loss of barrier element function upon CGGBP1 knockdown.

		Loss of CTCF binding and barrier activity		Gain of CTCF binding and barrier activity	
H3K9me3	Number of regions	N = 663		N = 216	
	Paired T-test p-values for signal in 10kb flanks	CT	KD	CT	KD
		<0.0001	<0.0001	<0.0001	<0.0001
RNA-seq [GSM1081538 and GSM1081539]	Paired T-test p-values for signal in 10kb flanks	0.0451	Data not available	0.2731	Data not available
H3K27me3	Number of regions	N = 27		N = 26	
	Paired T-test p-values for signal in 10kb flanks	CT	KD	CT	KD
		0.0027	0.9001	0.2521	0.0456
H3K4me3	Number of regions	N = 0		N = 0	
	Paired T-test p-values for signal in 10kb flanks	CT	KD	CT	KD
		-	-	-	-

Table S16: Table shows the number of CGGBP1-dependent CTCF binding sites showing variations in histone modification ChIP-seq signals and RNA-seq signal in 10kb flanks. The p-values of paired T-tests show a strong lack of co-variance of cumulative signals for H3K9me3 and RNA-seq and establish a chromatin barrier-like function of these 879 regions. For the regions with H3K27me3 variation, p values indicate a significant but milder barrier activity. For H3K4me3, no such regions were identified. CGGBP1-dependent CTCF binding sites thus seem to exert their barrier activity primarily through silencing histone mark H3K9me3 and to some extent H3K27me3.
