# Supplementary material for: CGGBP1 regulates CTCF occupancy at repeats
Source: Epigenetics Chromatin. 2019 Sep 23;12:57. doi: 10.1186/s13072-019-0305-6 (PMC6757366; doi:10.1186/s13072-019-0305-6)
Supplement: Supplementary file 2 — Additional file 2: Figure S1. (A) Human juvenile fibroblasts co-immunostained for CGGBP1 (Green) and CTCF (Red). Nuclei were counterstained with DAPI (blue). Mean fluorescence intensities for CGGBP1 (green) and CTCF (red) were normalized along the line-marked segment and plotted using ImageJ. Normalized signals along the line segment drawn through a midbody shows colocalization of CGGBP1 and CTCF. (B) Human juvenile fibroblasts co-immunostained for CGGBP1 (red) and CTCF (Green). Nuclei were counterstained with DAPI (blue). All images were captured with confocal plane of 1.601 µm. Figure S2. PLA (red foci) confirms CTCF-CGGBP1 interaction in situ. Nuclei were stained with DAPI (blue). CGGBP1-CTCF interaction was stronger in the nuclei than in cytoplasm (inset of mouse anti-CGGBP1:rabbit anti-CTCF sample). No significant interaction was observed in IgG and no-primary antibody negative controls (inset of no-primary antibody sample). All images were captured with confocal plane of 1.601 µm. Figure S3. Cytoplasmic and nuclear fractions were separated from HEK293T cells by REAP protocol. The upper panel shows immunoblot results for cytoplasmic marker GAPDH. The middle and the lower panels show immunoblot results for a nuclear protein Histone H3 using two different antibodies (H3K4me3 and H3K27me3). Equal volumes of cytoplasmic and nuclear fraction lysates were run in the lanes. Figure S4. The closest distance between starved RM CGGBP1 peak midpoint and transcription factor peak midpoint was determined by using bedtools closest. Frequency distribution of closest distances was plotted in bin of 0.5 kb for starved RM CGGBP1 peaks (A) and stimulated RM CGGBP1 peaks (B). Figure S5. HEK293T cells were transduced with control shRNA lentivirus, CGGBP1-shRNA lentivirus and CGGBP1-overexpression lentivirus, respectively. The upper panel shows immunoblot results for CGGBP1 and lower panel shows same for GAPDH loading control. Figure S6. The distribution of CTCF reads for repeat-mask [file 13072_2019_305_MOESM2_ESM.pdf]

Figure S1

A

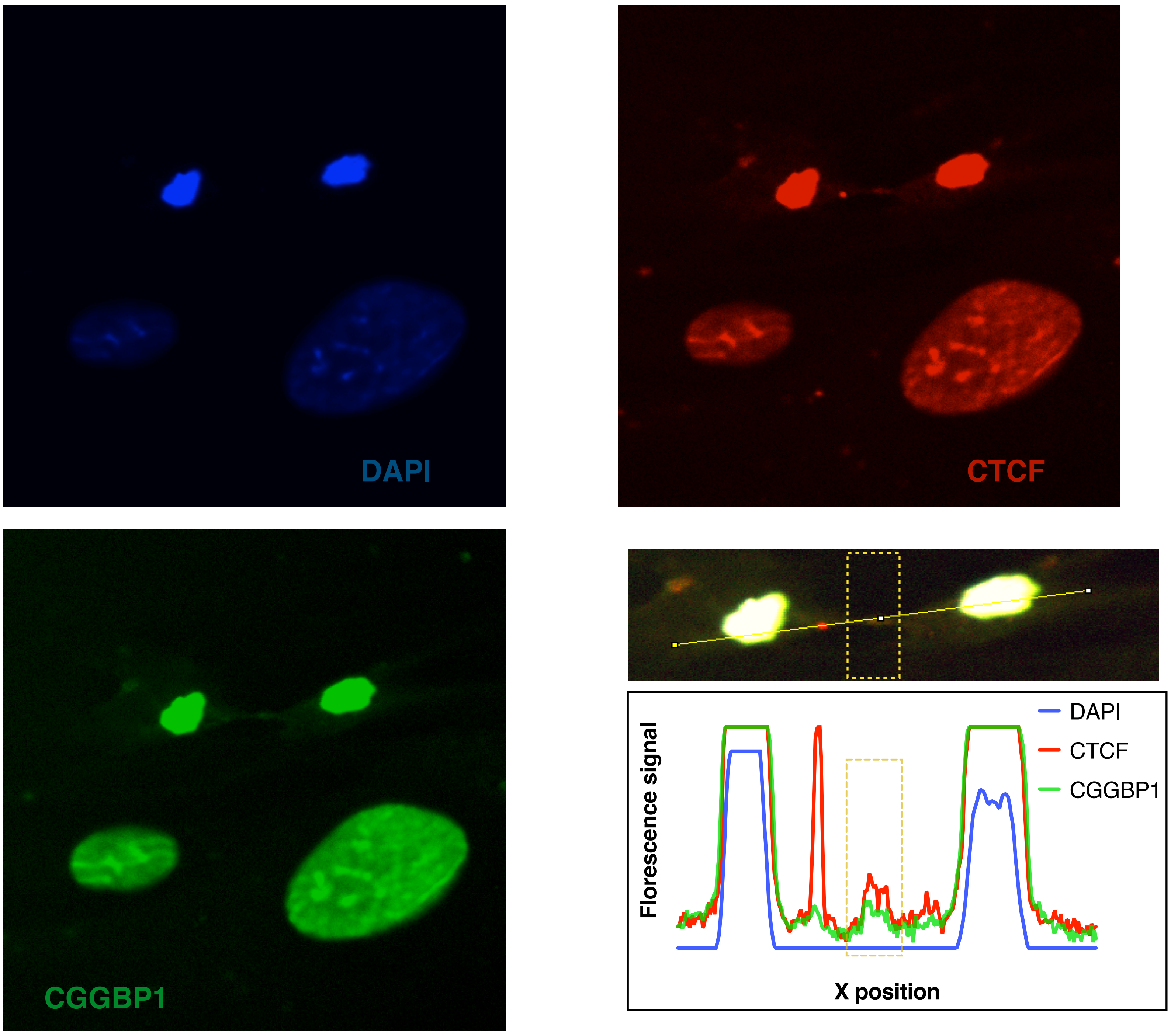
DAPI
CTCF
CGGBP1
DAPI
CTCF
CGGBP1
Florescence signal
X position

B

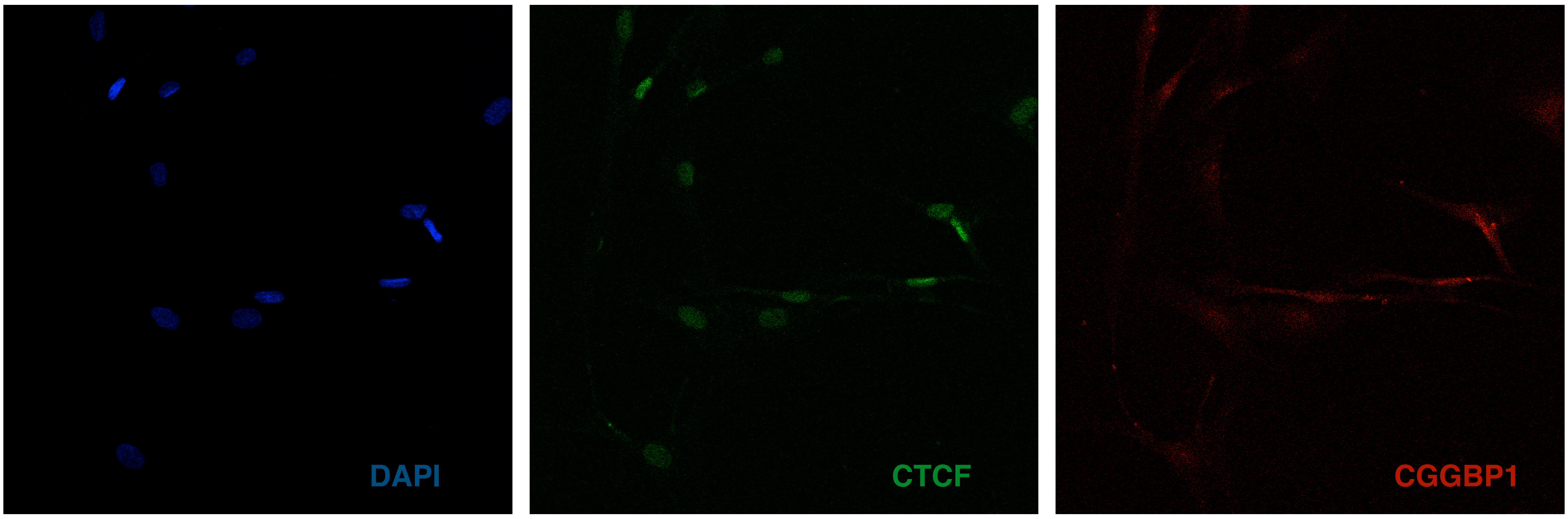
DAPI
CTCF
CGGBP1

Figure S2

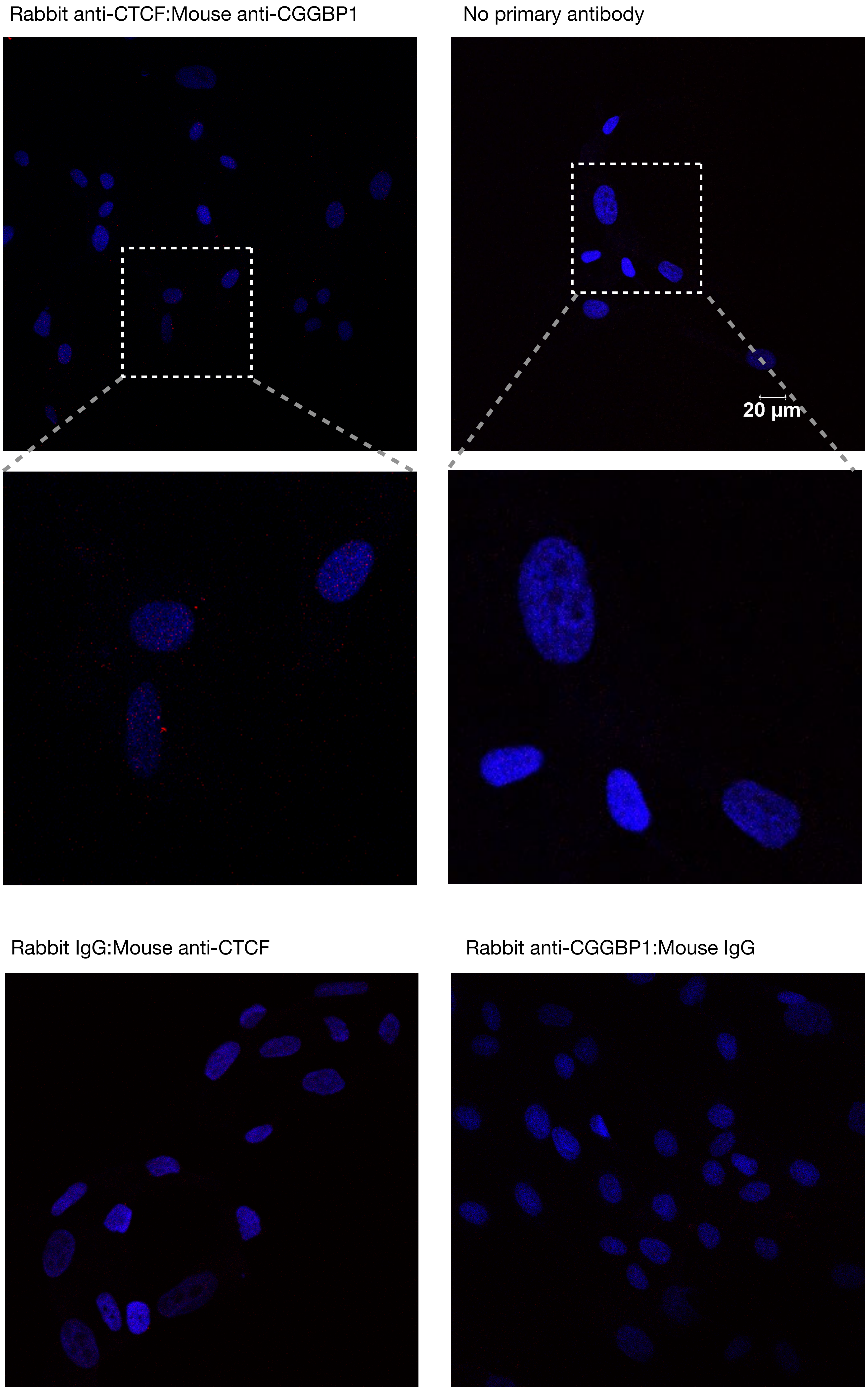
Rabbit anti-CTCF:Mouse anti-CGGBP1
No primary antibody
20 µm
Rabbit IgG:Mouse anti-CTCF
Rabbit anti-CGGBP1:Mouse IgG

Figure S3

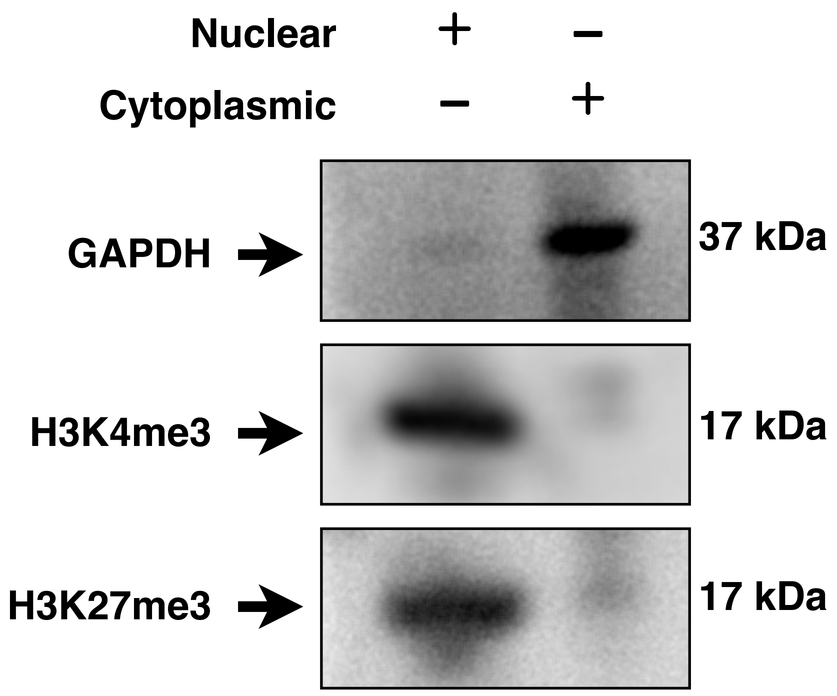

Nuclear + –
Cytoplasmic – +
GAPDH
37 kDa
H3K4me3
17 kDa
H3K27me3
17 kDa

Figure S4

A

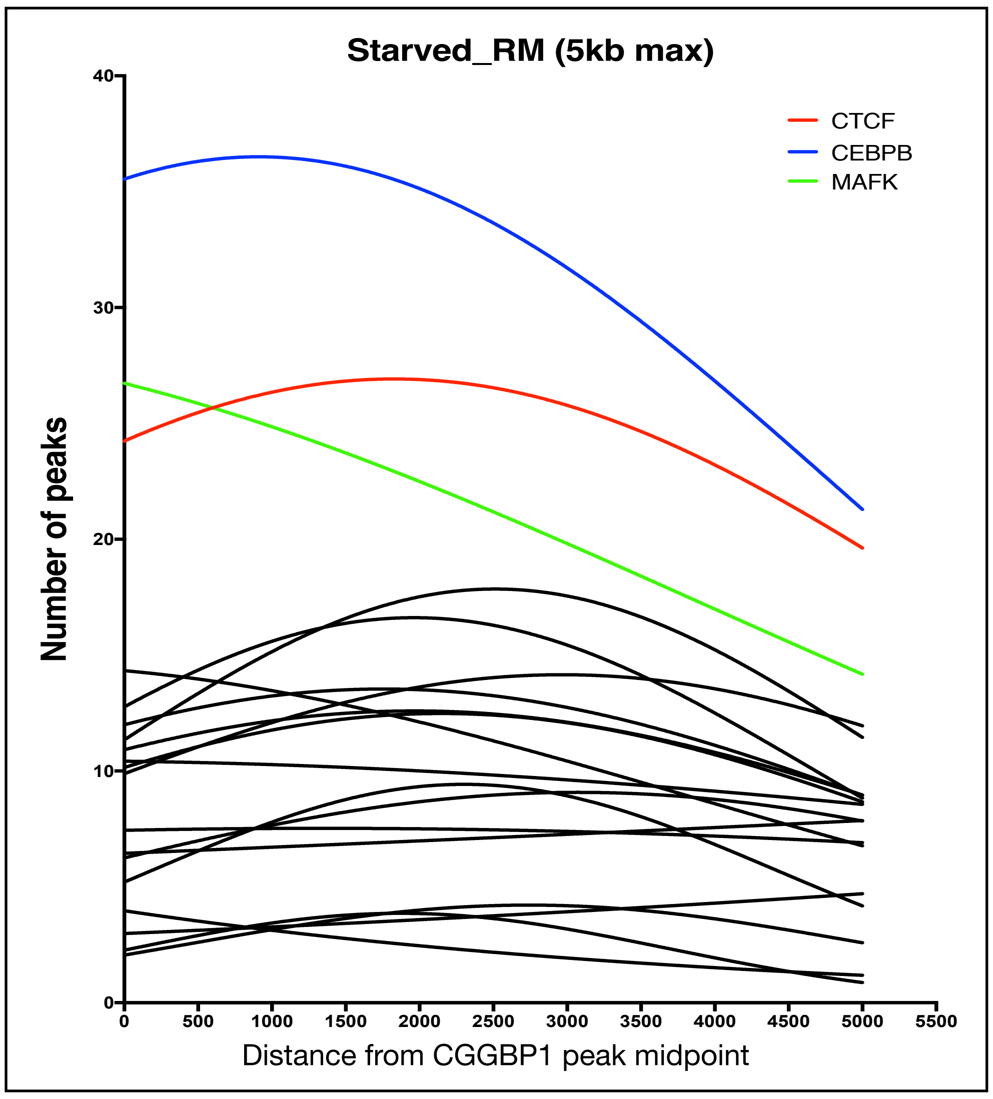

Starved_RM (5kb max)
CTCF
CEBPB
MAFK
Number of peaks
0
10
20
30
40
0
500
1000
1500
2000
2500
3000
3500
4000
4500
5000
5500
Distance from CGGBP1 peak midpoint

B

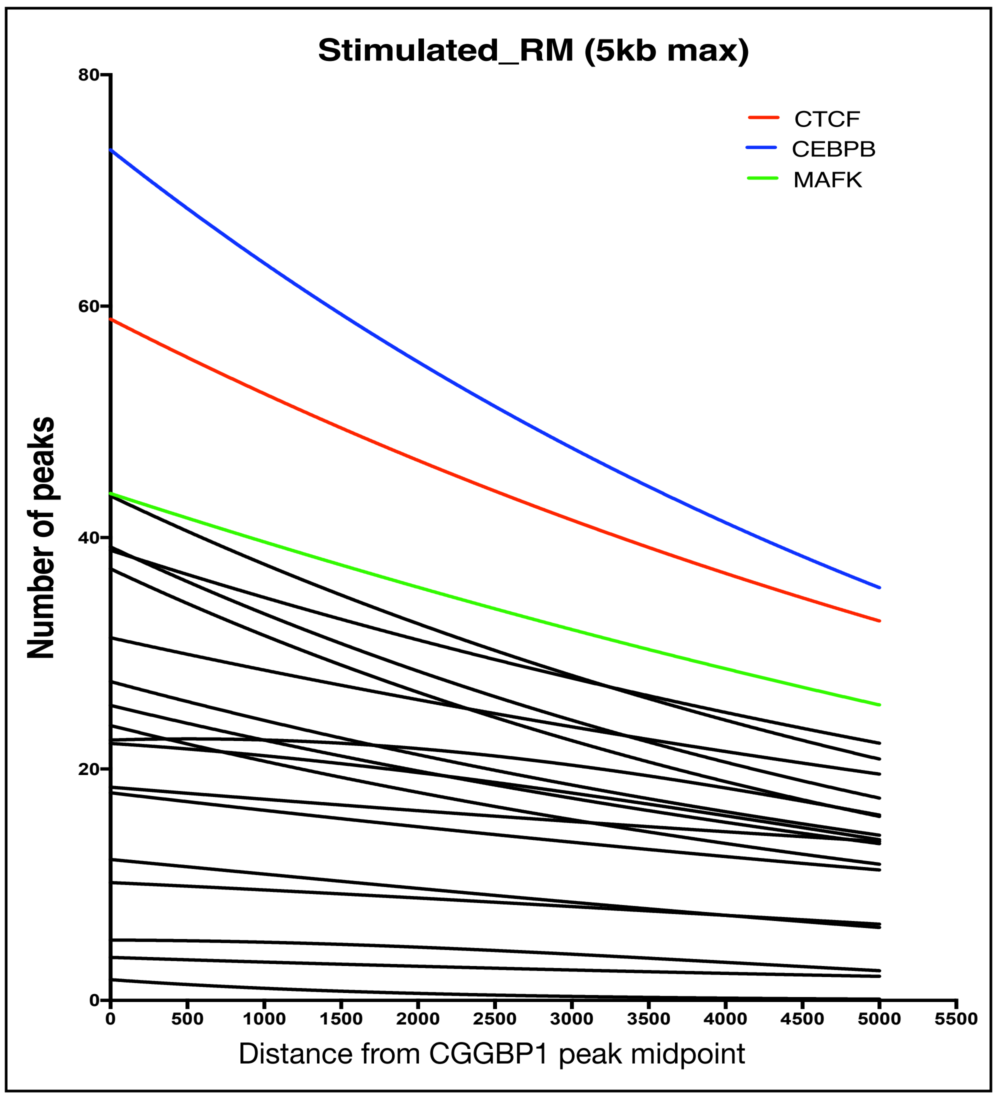

Stimulated_RM (5kb max)
CTCF
CEBPB
MAFK
Number of peaks
0
20
40
60
80
0
500
1000
1500
2000
2500
3000
3500
4000
4500
5000
5500
Distance from CGGBP1 peak midpoint

Figure S5

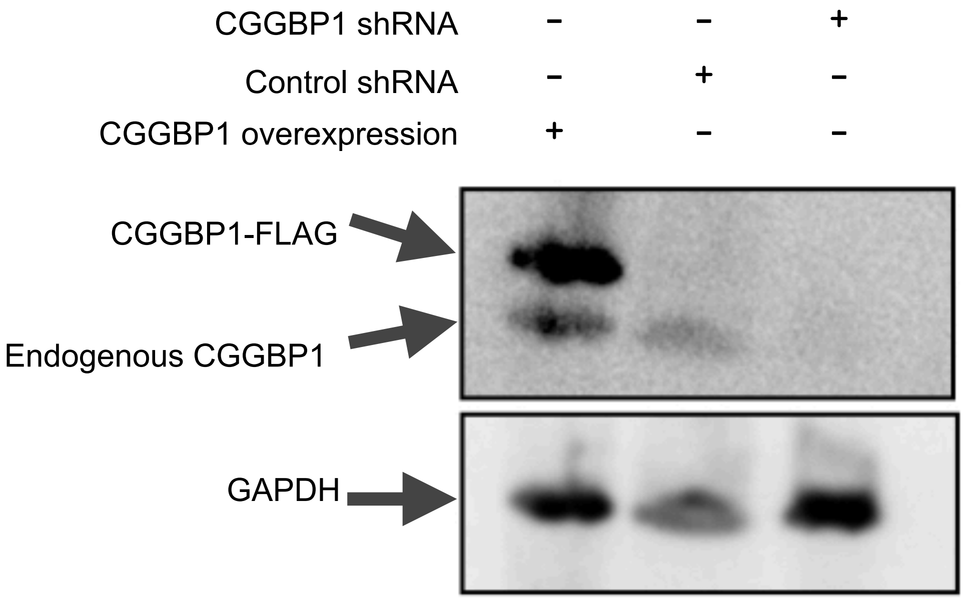
CGGBP1 shRNA – – +
Control shRNA – + –
CGGBP1 overexpression + – –
CGGBP1-FLAG
Endogenous CGGBP1
GAPDH

Figure S6

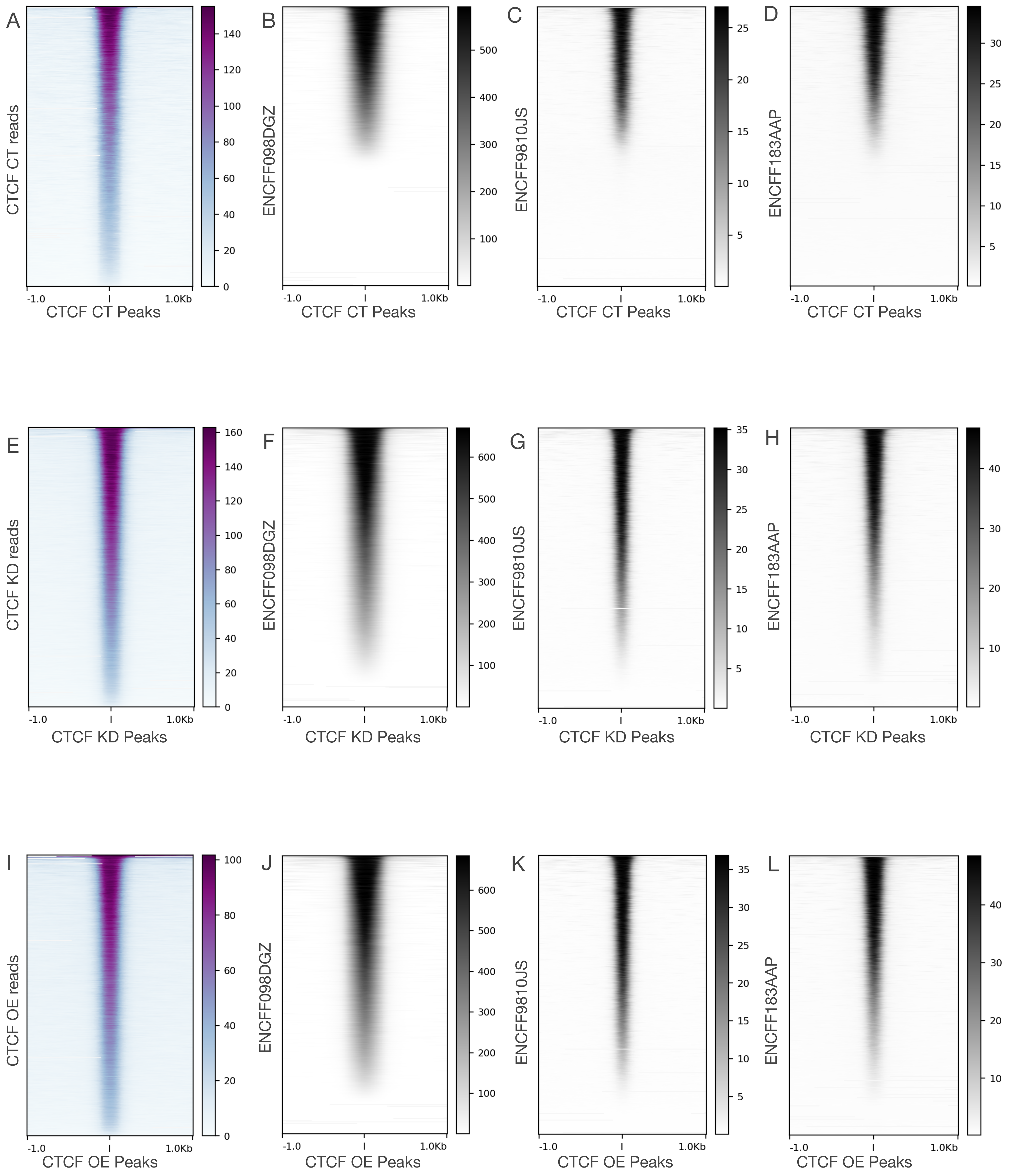

A
B
C
D
CTCF CT reads
ENCFF098DGZ
ENCFF9810JS
ENCFF183AAP
CTCF CT Peaks
E
F
G
H
CTCF KD reads
CTCF KD Peaks
I
J
K
L
CTCF OE reads
CTCF OE Peaks
-1.0
1.0Kb

Figure S7

A

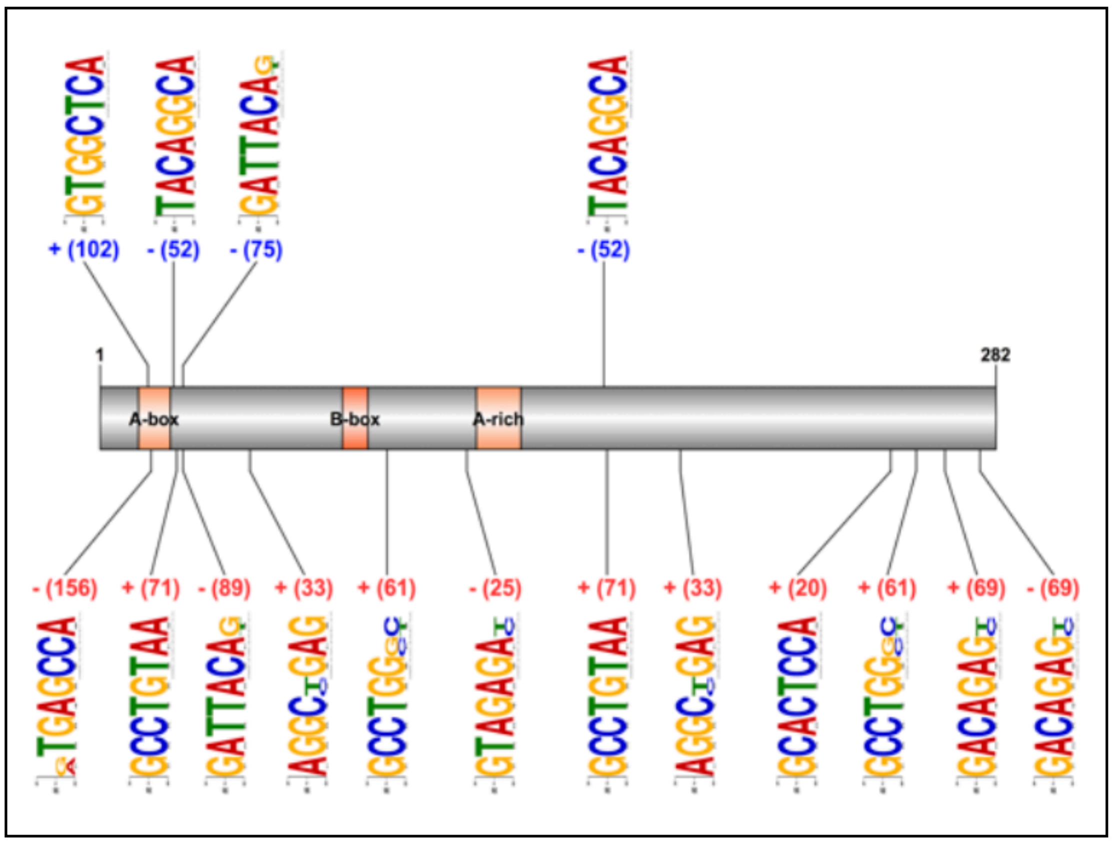

Number of Alu subsequences (SS) = 8

% of RM stimulated CGGBP1 peaks with Alu SS = 20.02

% of RM CT peaks with Alu SS = 6.83

% of RM KD peaks with Alu SS = 23.20

% of RM OE peaks with Alu SS = 22.03

B

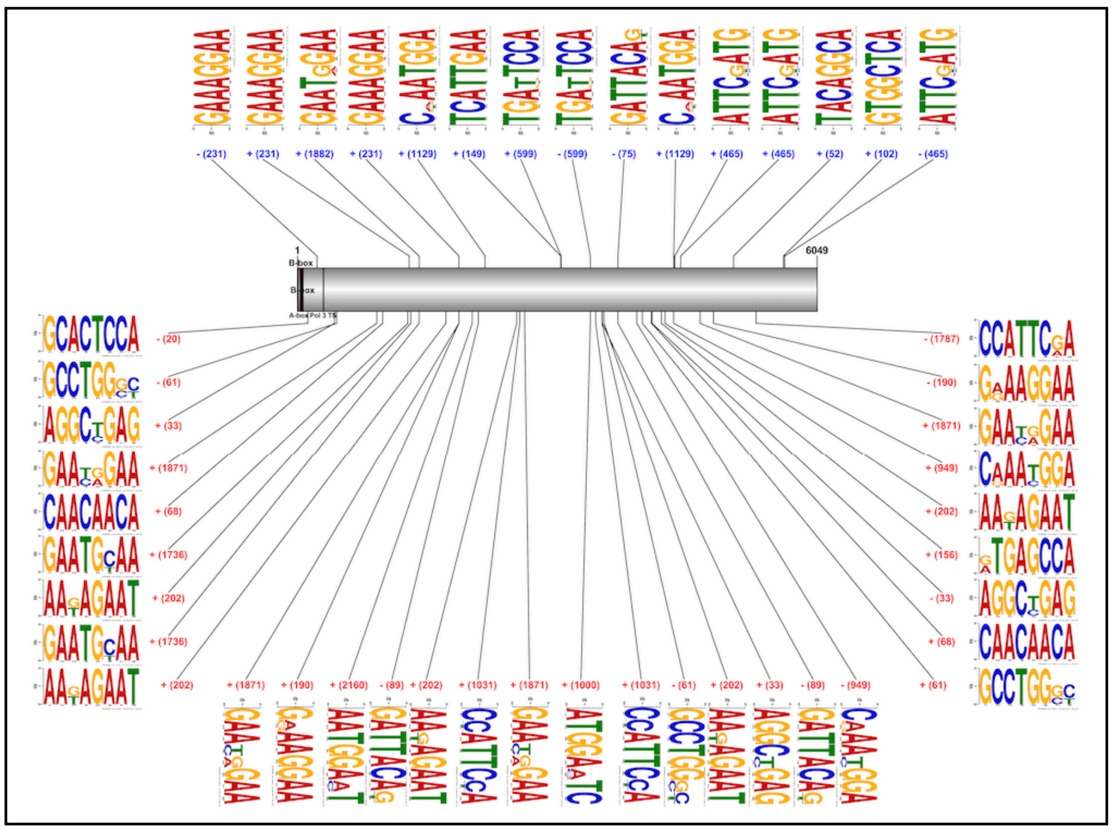

Number of L1 subsequences (SS) = 15

% of RM stimulated CGGBP1 peaks with L1 SS = 44.38

% of RM CT peaks with L1 SS = 15.48

% of RM KD peaks with L1 SS = 52.67

% of RM OE peaks with L1 SS = 51.03

Figure S8

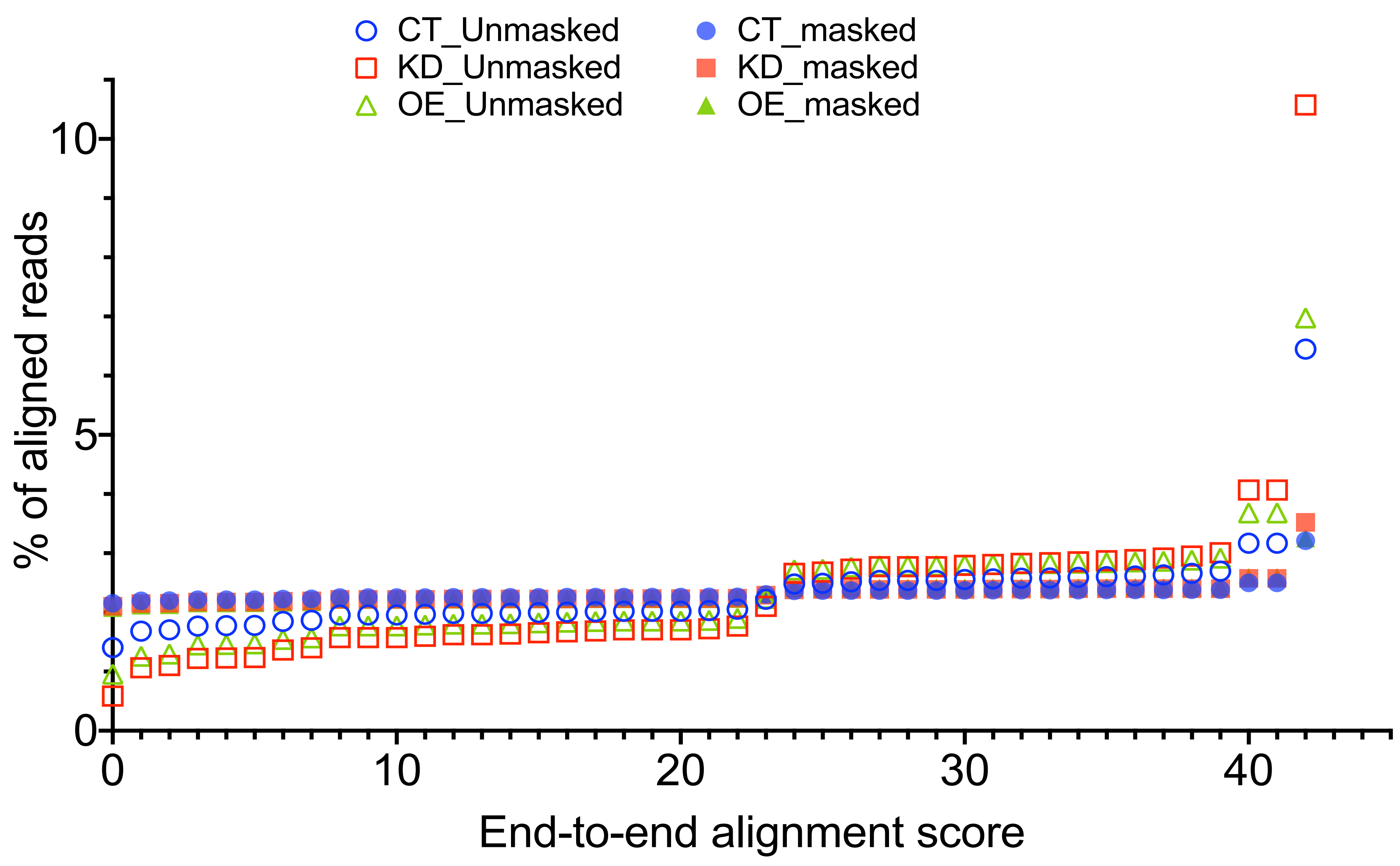
CT_Unmasked
KD_Unmasked
OE_Unmasked
CT_masked
KD_masked
OE_masked
% of aligned reads
0
5
10
0
10
20
30
40
End-to-end alignment score

Figure S9

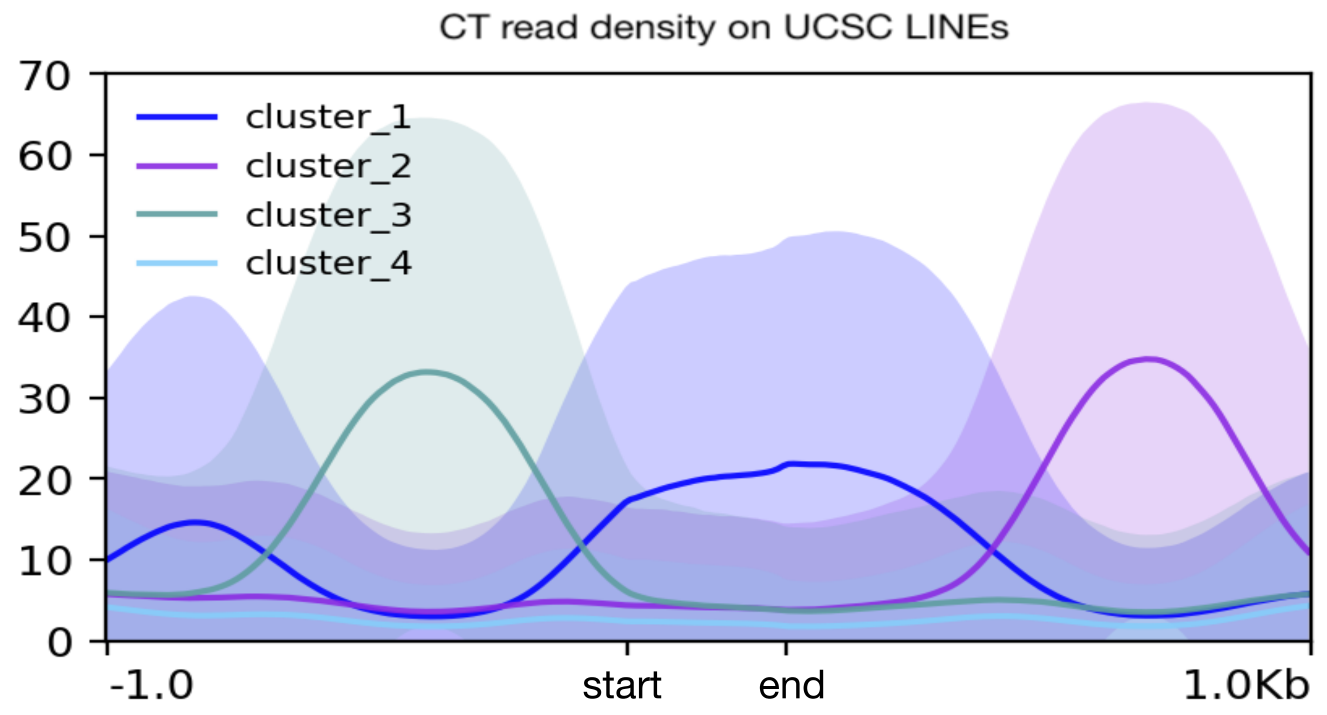

CT read density on UCSC LINEs
70
60
50
40
30
20
10
0
cluster_1
cluster_2
cluster_3
cluster_4
-1.0
start
end
1.0Kb

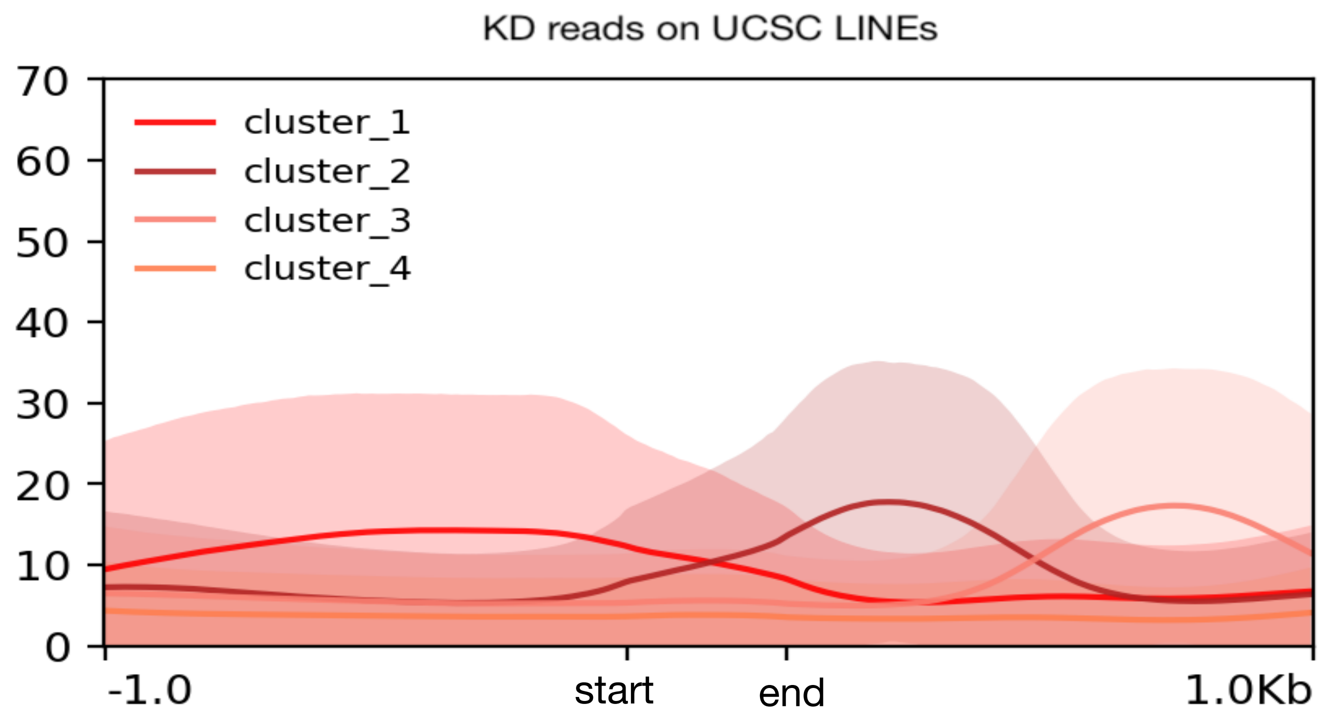

KD reads on UCSC LINEs
70
60
50
40
30
20
10
0
cluster_1
cluster_2
cluster_3
cluster_4
-1.0
start
end
1.0Kb

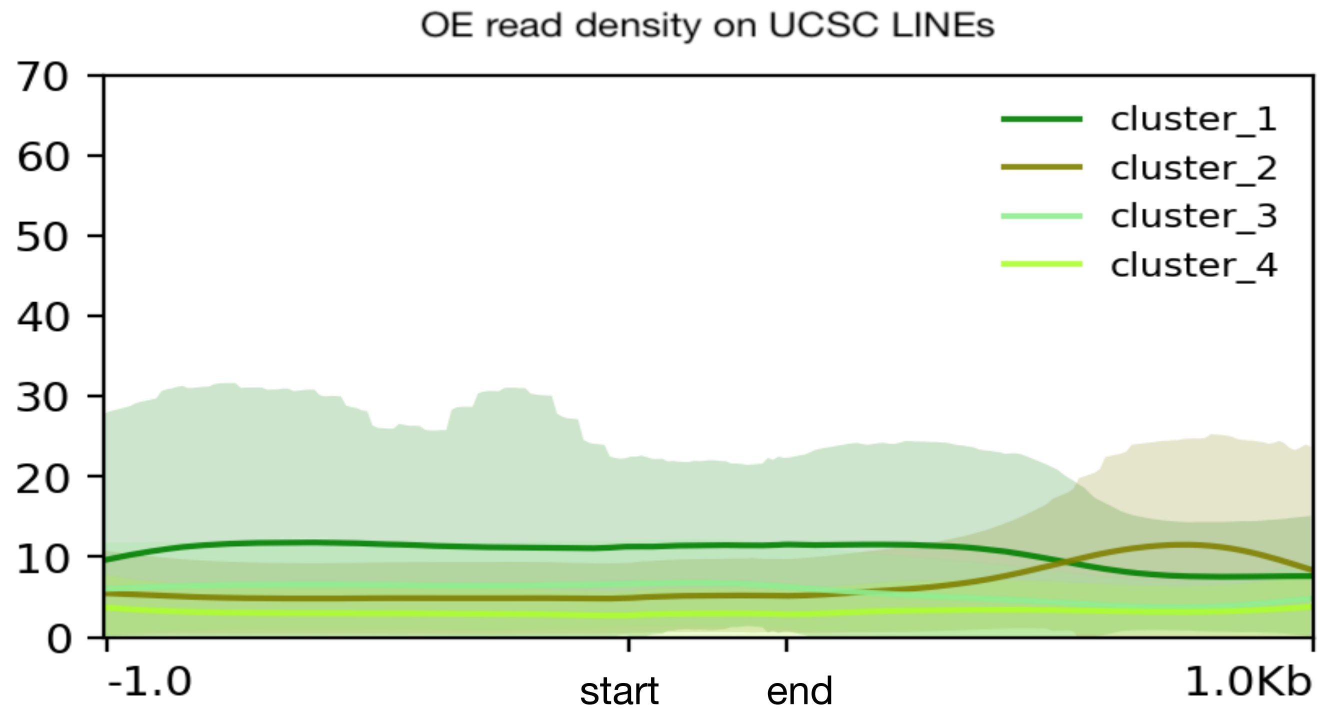

OE read density on UCSC LINEs
70
60
50
40
30
20
10
0
cluster_1
cluster_2
cluster_3
cluster_4
-1.0
start
end
1.0Kb

Figure S10

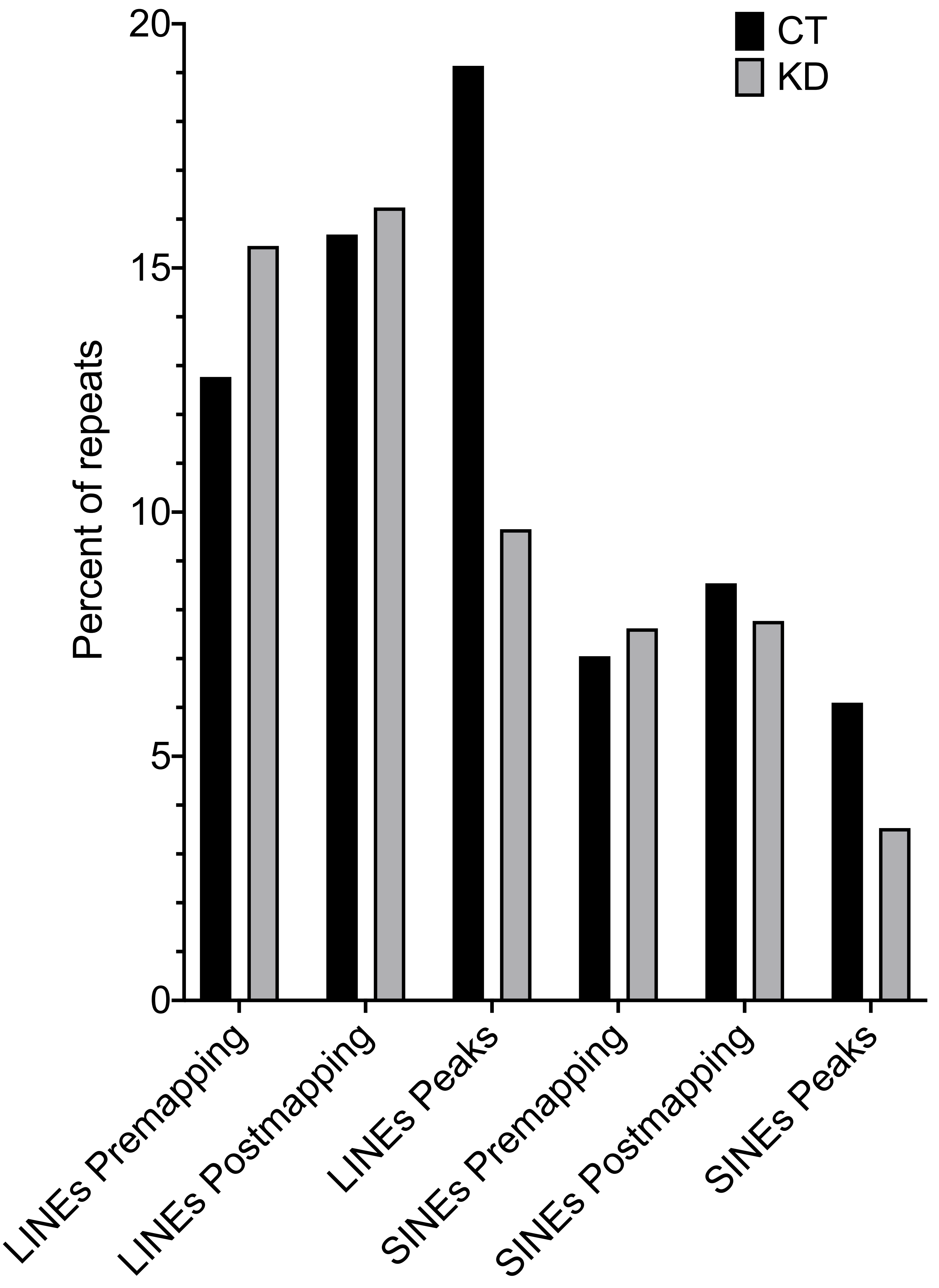

CT
KD
Percent of repeats
20
15
10
5
0
LINEs Premapping
LINEs Postmapping
LINEs Peaks
SINEs Premapping
SINEs Postmapping
SINEs Peaks

Figure S11

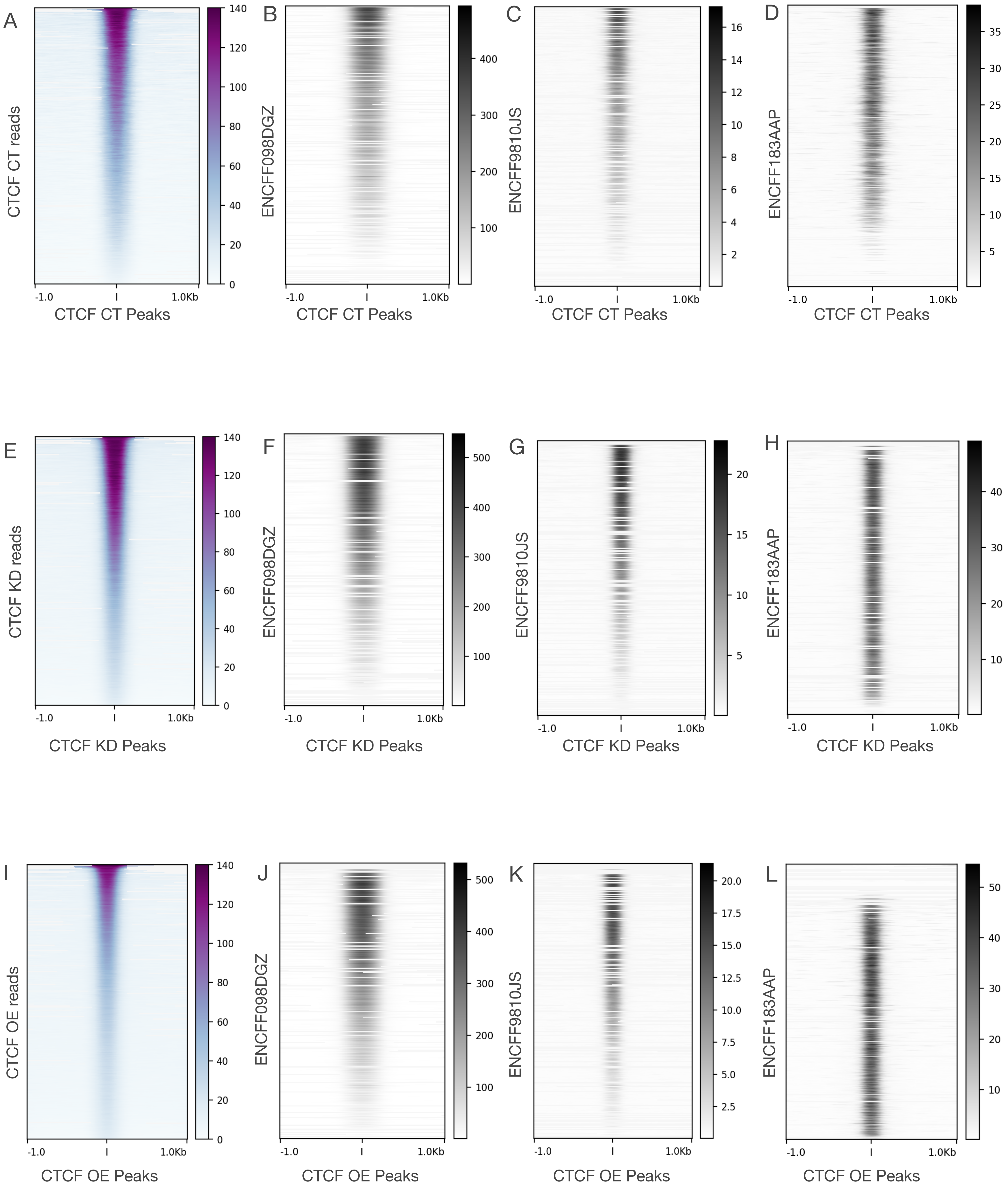

A
CTCF CT reads
CTCF CT Peaks
B
ENCFF098DGZ
CTCF CT Peaks
C
ENCFF9810JS
CTCF CT Peaks
D
ENCFF183AAP
CTCF CT Peaks
E
CTCF KD reads
CTCF KD Peaks
F
ENCFF098DGZ
CTCF KD Peaks
G
ENCFF9810JS
CTCF KD Peaks
H
ENCFF183AAP
CTCF KD Peaks
I
CTCF OE reads
CTCF OE Peaks
J
ENCFF098DGZ
CTCF OE Peaks
K
ENCFF9810JS
CTCF OE Peaks
L
ENCFF183AAP
CTCF OE Peaks
-1.0
1.0Kb

Figure S12

A

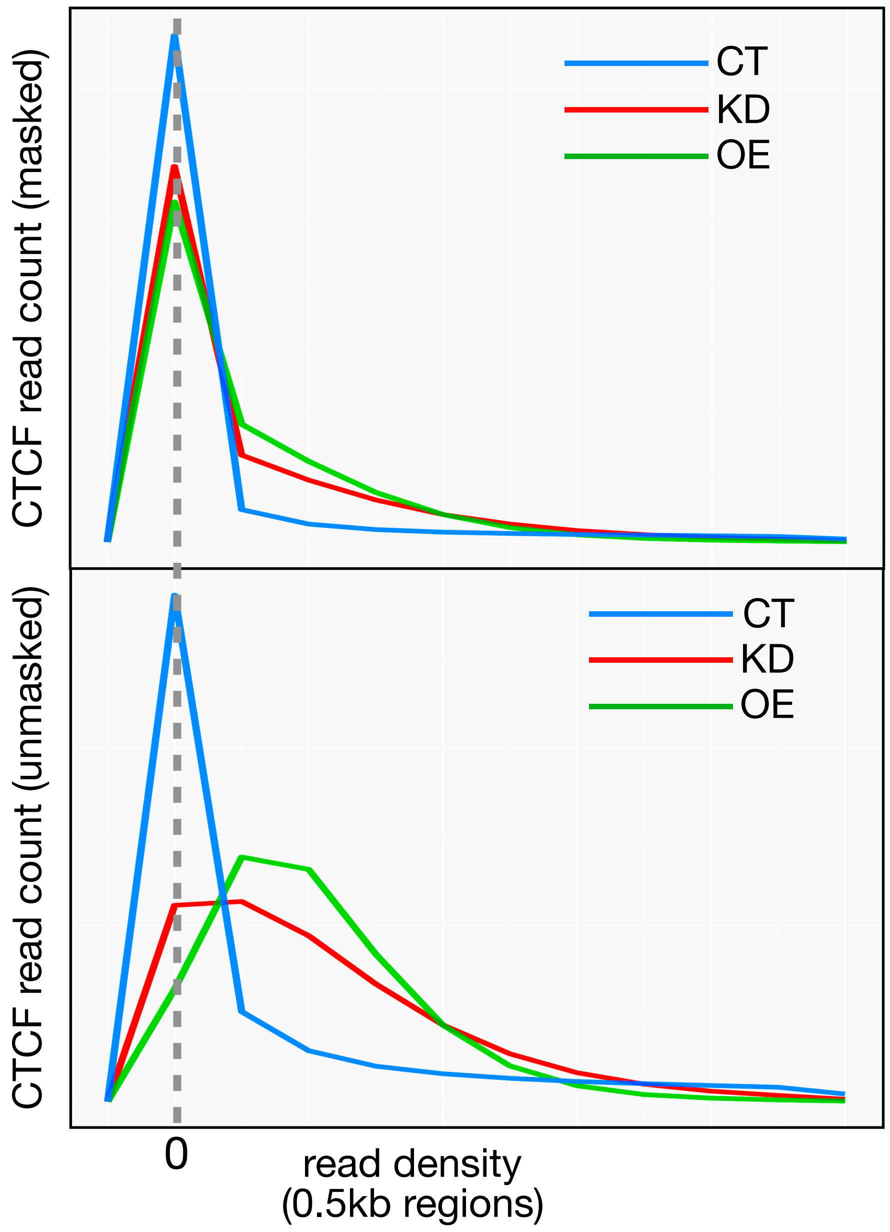

B

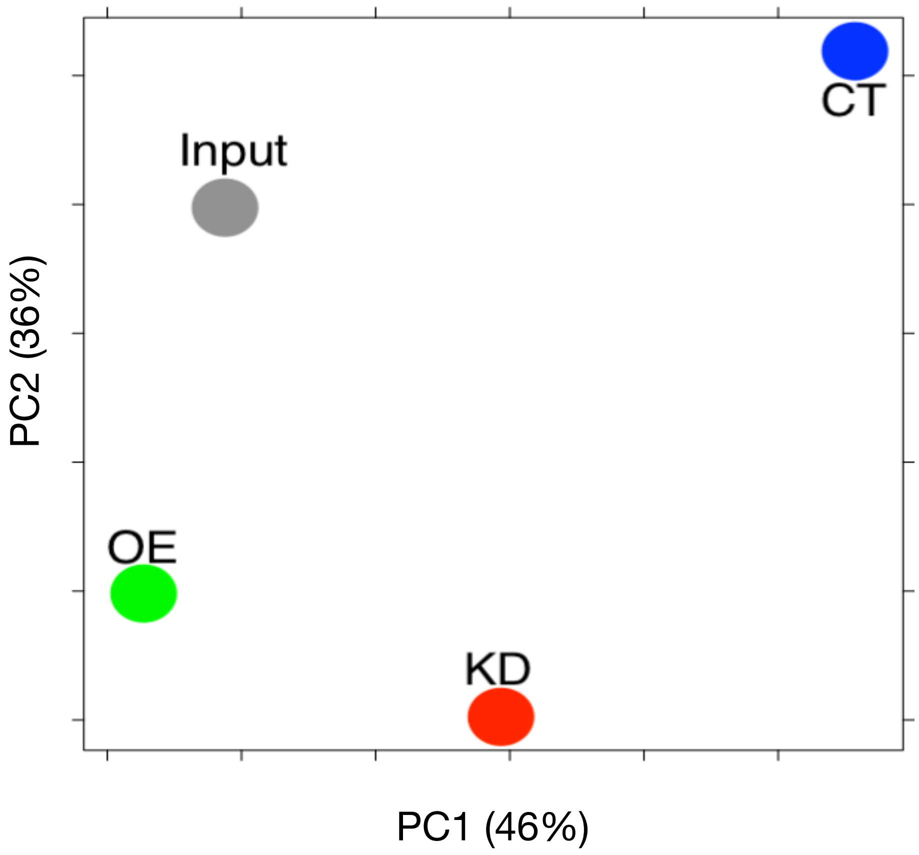

Figure S13

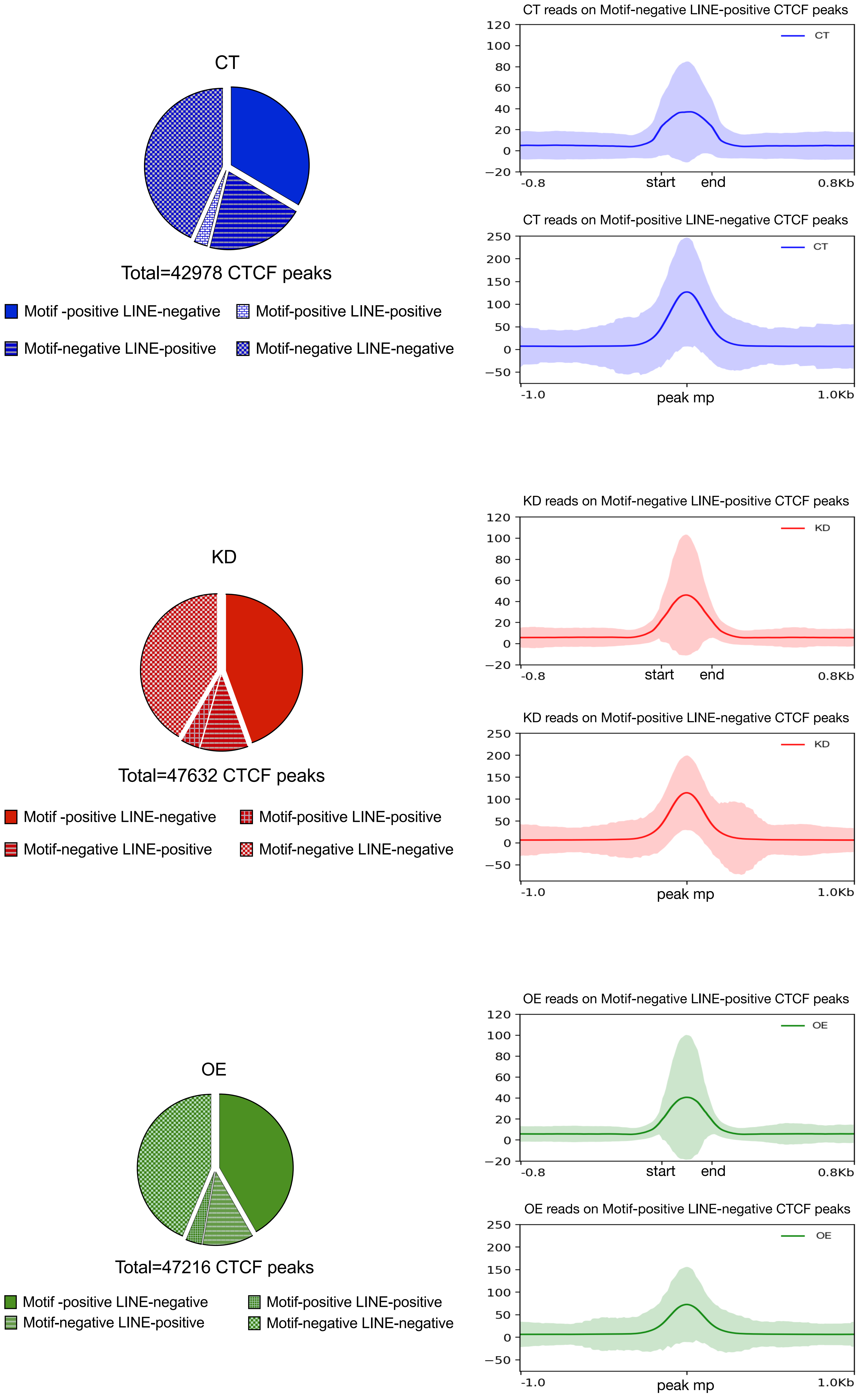
CT
Total=42978 CTCF peaks
Motif -positive LINE-negative
Motif-positive LINE-positive
Motif-negative LINE-positive
Motif-negative LINE-negative
CT reads on Motif-negative LINE-positive CTCF peaks
CT
start
end
-0.8
0.8Kb
CT reads on Motif-positive LINE-negative CTCF peaks
CT
-1.0
peak mp
1.0Kb
KD
Total=47632 CTCF peaks
Motif -positive LINE-negative
Motif-positive LINE-positive
Motif-negative LINE-positive
Motif-negative LINE-negative
KD reads on Motif-negative LINE-positive CTCF peaks
KD
start
end
-0.8
0.8Kb
KD reads on Motif-positive LINE-negative CTCF peaks
KD
-1.0
peak mp
1.0Kb
OE
Total=47216 CTCF peaks
Motif -positive LINE-negative
Motif-positive LINE-positive
Motif-negative LINE-positive
Motif-negative LINE-negative
OE reads on Motif-negative LINE-positive CTCF peaks
OE
start
end
-0.8
0.8Kb
OE reads on Motif-positive LINE-negative CTCF peaks
OE
-1.0
peak mp
1.0Kb

Figure S14

A

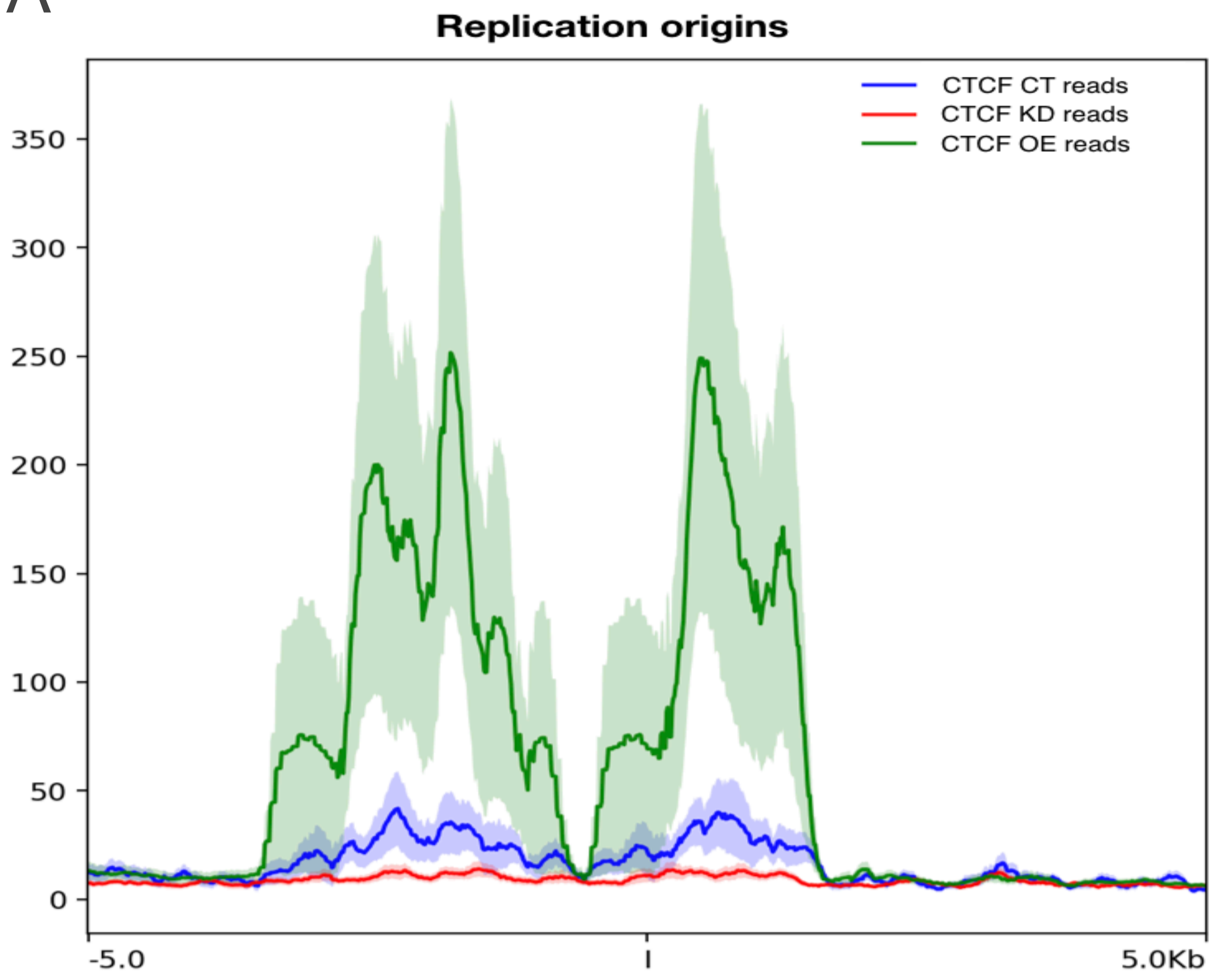

B

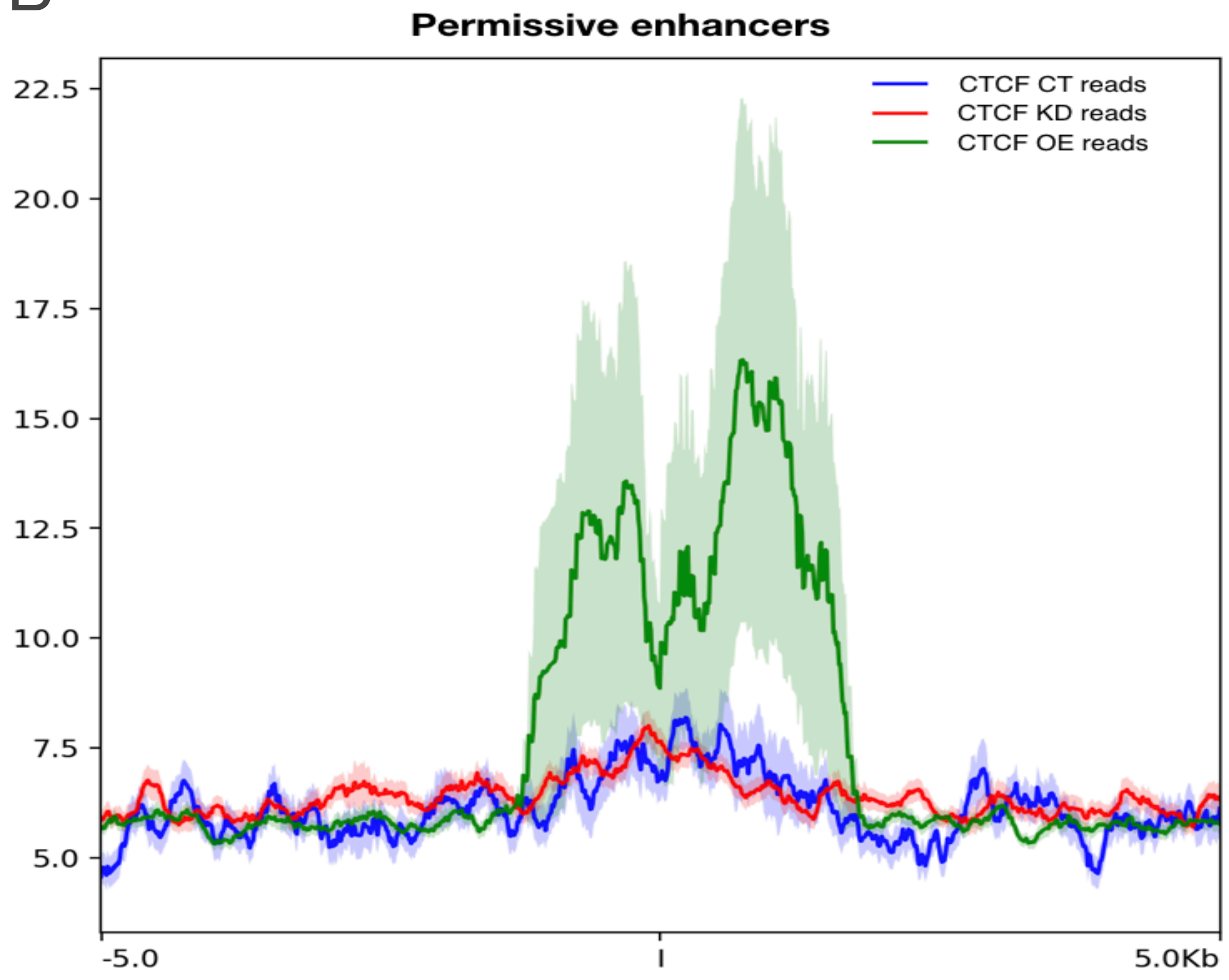

Figure S15

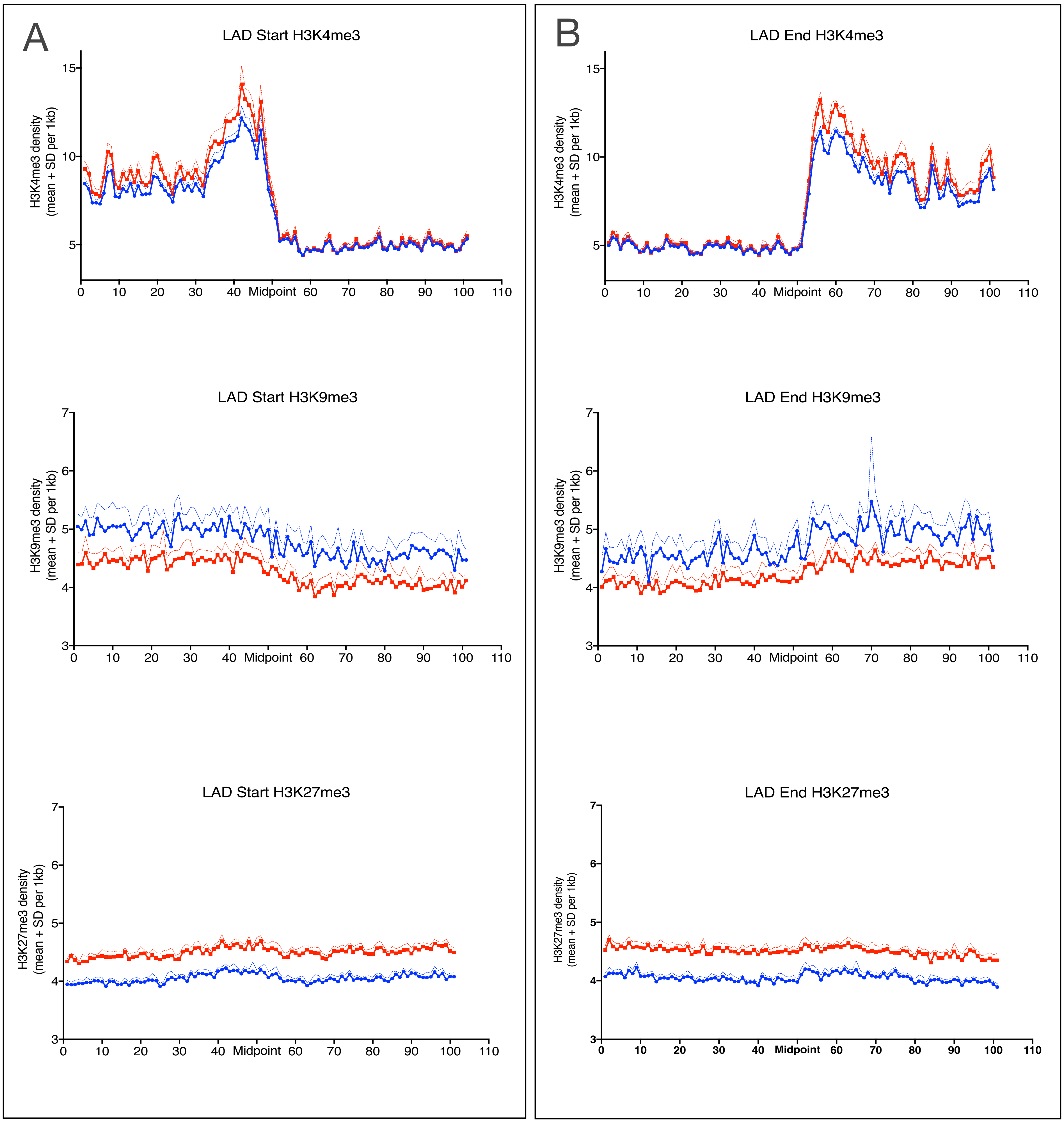
A
LAD Start H3K4me3
H3K4me3 density (mean + SD per 1kb)
LAD Start H3K9me3
H3K9me3 density (mean + SD per 1kb)
LAD Start H3K27me3
H3K27me3 density (mean + SD per 1kb)
B
LAD End H3K4me3
H3K4me3 density (mean + SD per 1kb)
LAD End H3K9me3
H3K9me3 density (mean + SD per 1kb)
LAD End H3K27me3
H3K27me3 density (mean + SD per 1kb)
0 10 20 30 40 Midpoint 60 70 80 90 100 110

Figure S16

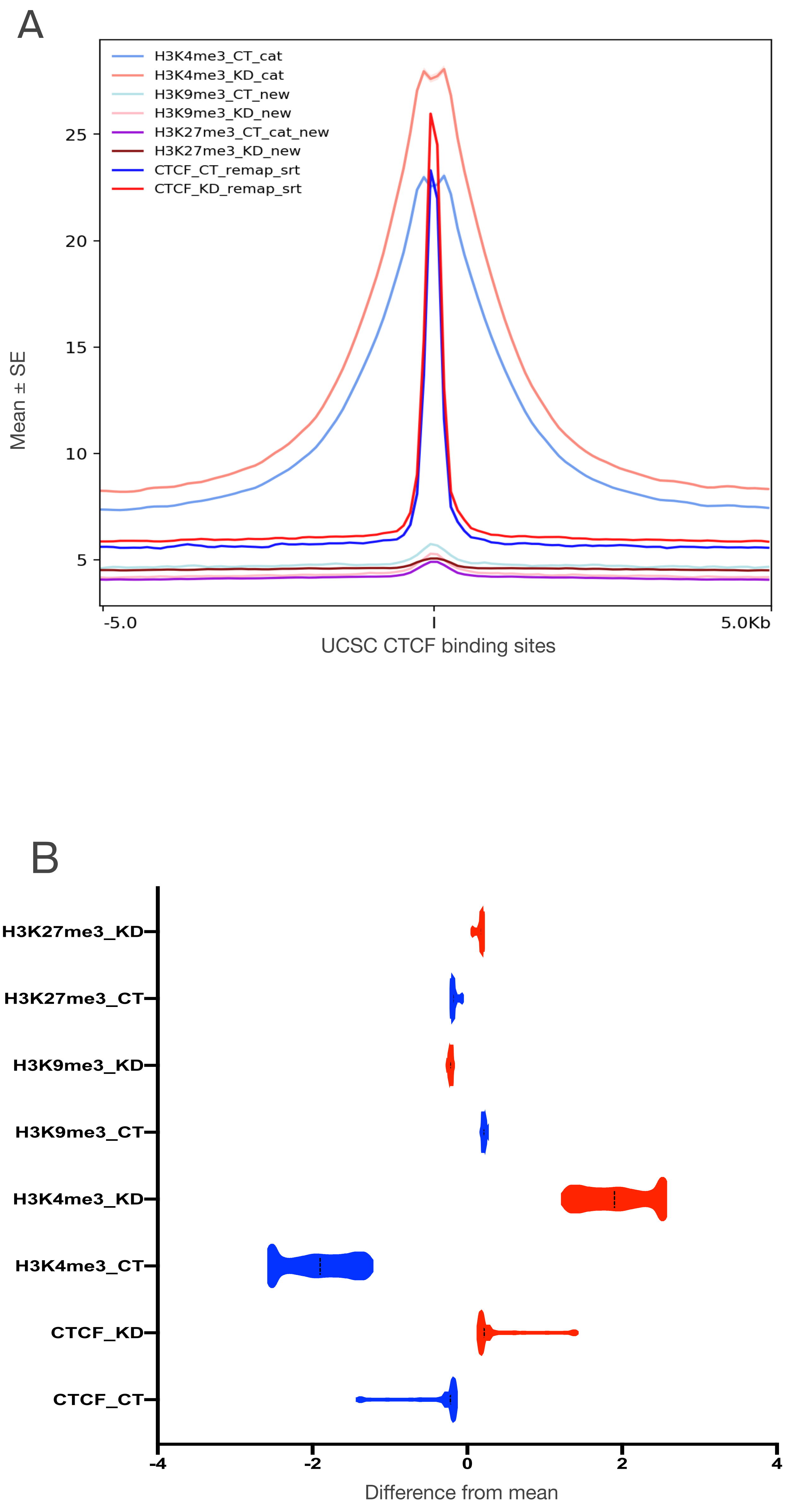

A
H3K4me3_CT_cat
H3K4me3_KD_cat
H3K9me3_CT_new
H3K9me3_KD_new
H3K27me3_CT_cat_new
H3K27me3_KD_new
CTCF_CT_remap_srt
CTCF_KD_remap_srt
Mean ± SE
25
20
15
10
5
-5.0
5.0Kb
UCSC CTCF binding sites
B
H3K27me3_KD
H3K27me3_CT
H3K9me3_KD
H3K9me3_CT
H3K4me3_KD
H3K4me3_CT
CTCF_KD
CTCF_CT
-4
-2
0
2
4
Difference from mean

Figure S17

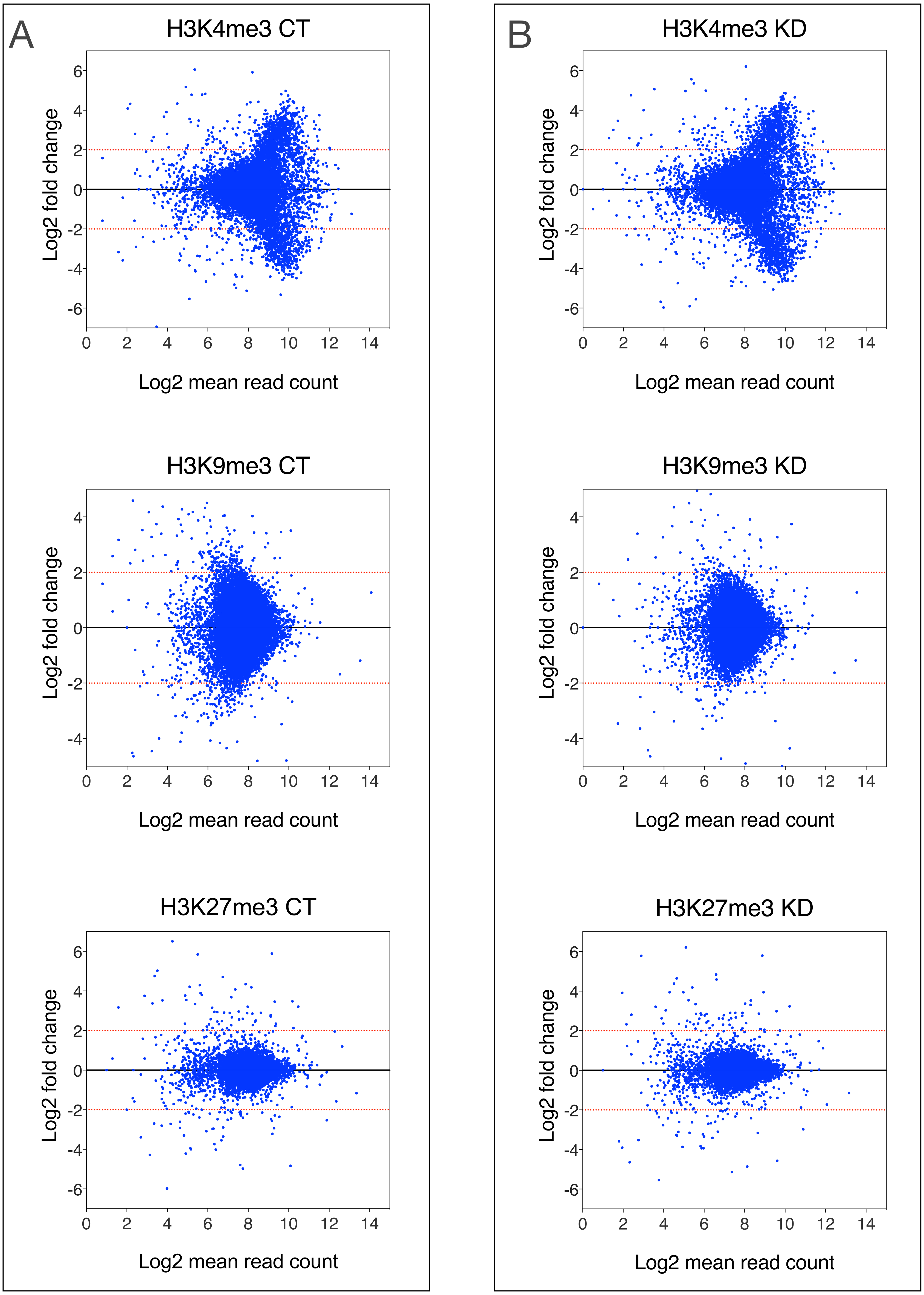

A
H3K4me3 CT
H3K9me3 CT
H3K27me3 CT
B
H3K4me3 KD
H3K9me3 KD
H3K27me3 KD
Log2 fold change
Log2 mean read count

Figure S18

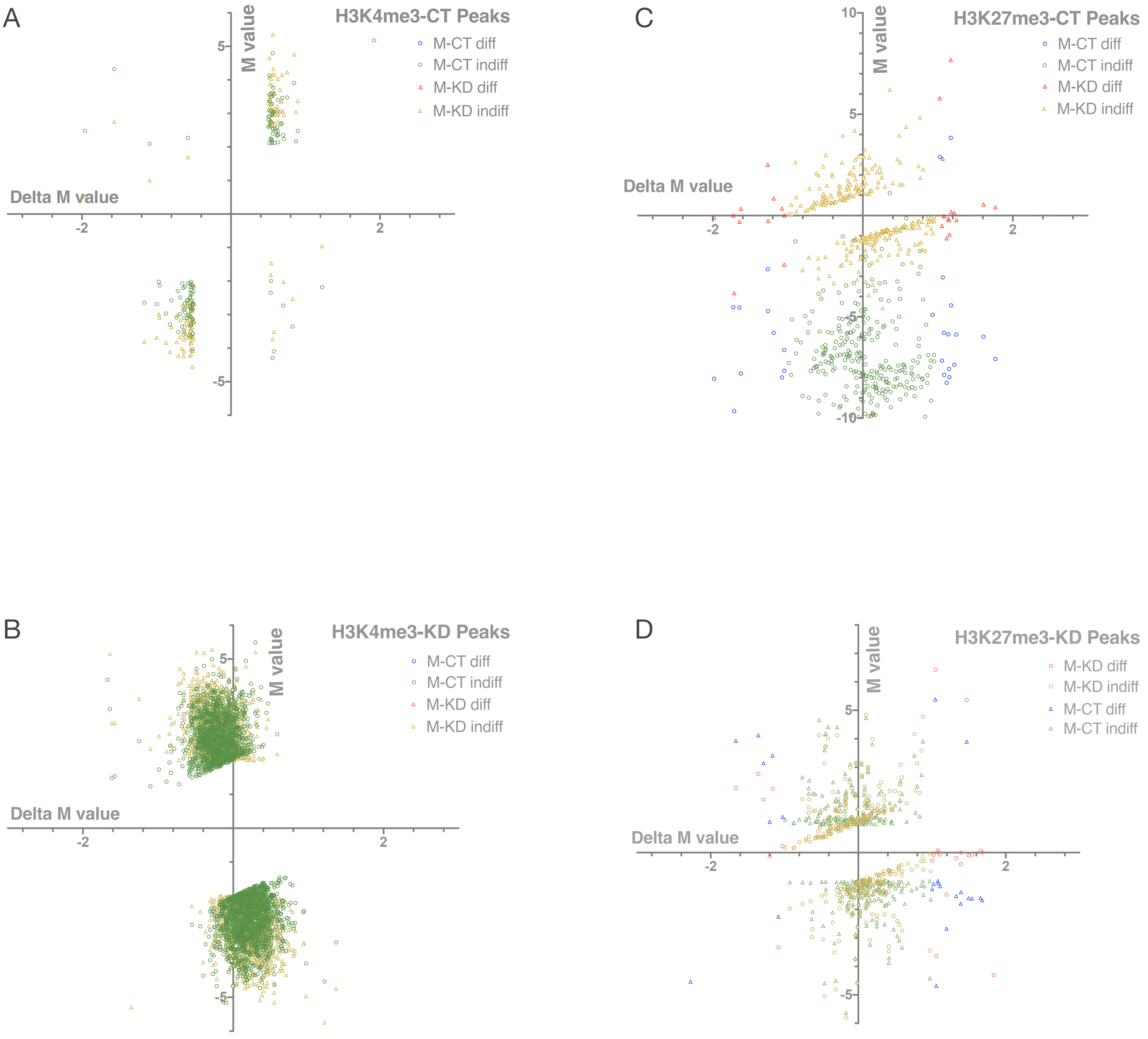
A
H3K4me3-CT Peaks
M-CT diff
M-CT indiff
M-KD diff
M-KD indiff
M value
Delta M value
5
-5
-2
2
B
H3K4me3-KD Peaks
M-CT diff
M-CT indiff
M-KD diff
M-KD indiff
M value
Delta M value
5
-5
-2
2
C
H3K27me3-CT Peaks
M-CT diff
M-CT indiff
M-KD diff
M-KD indiff
M value
Delta M value
10
5
-5
-10
-2
2
D
H3K27me3-KD Peaks
M-KD diff
M-KD indiff
M-CT diff
M-CT indiff
M value
Delta M value
5
-5
-2
2

Figure S19

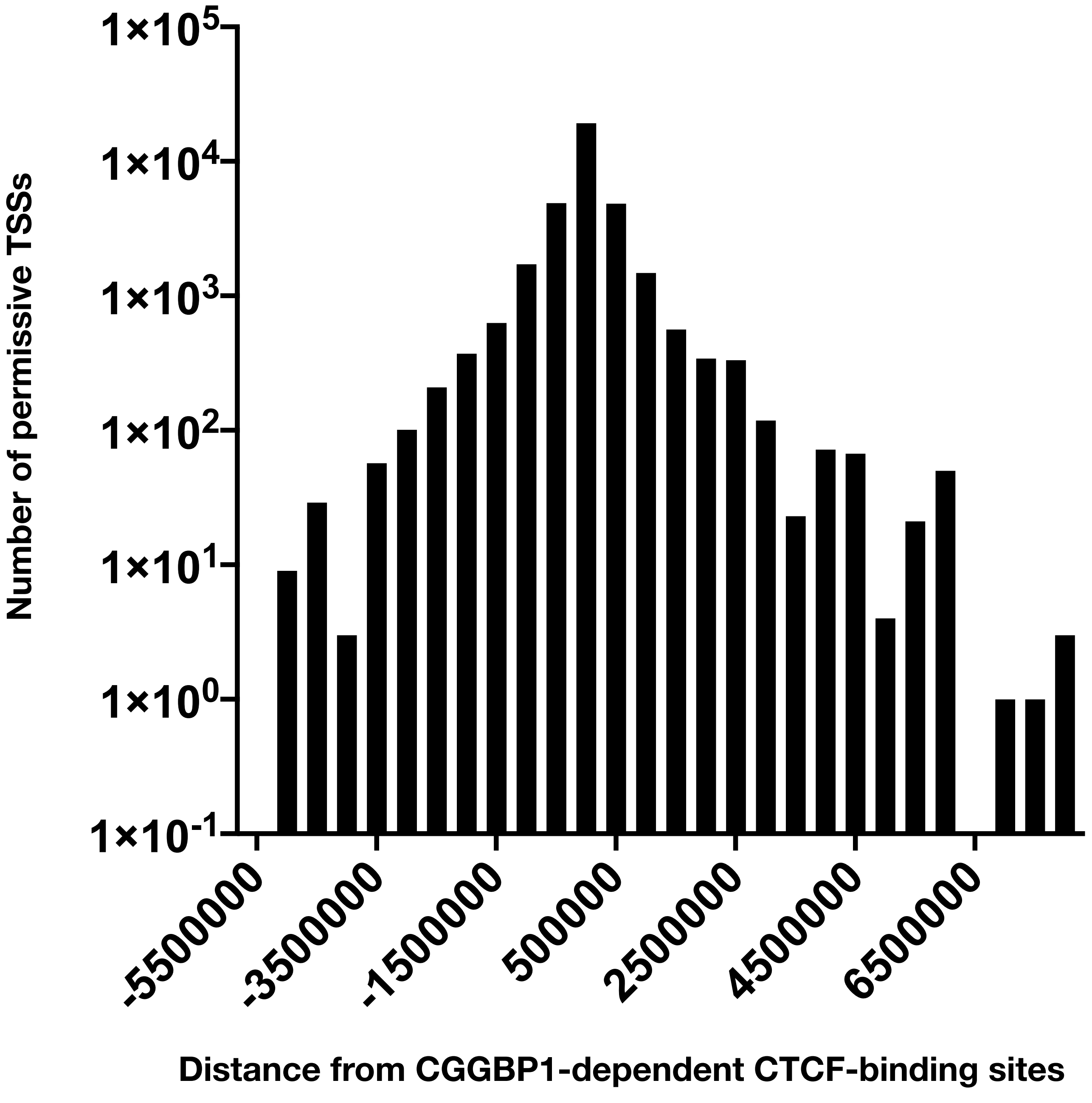

Number of permissive TSSs
1×10^5
1×10^4
1×10^3
1×10^2
1×10^1
1×10^0
1×10^-1
-5500000
-3500000
-1500000
500000
2500000
4500000
6500000
Distance from CGGBP1-dependent CTCF-binding sites
