# Supplementary material for: CGGBP1 regulates CTCF occupancy at repeats
Source: Epigenetics Chromatin. 2019 Sep 23;12:57. doi: 10.1186/s13072-019-0305-6 (PMC6757366; doi:10.1186/s13072-019-0305-6)
Supplement: Supplementary file 3 — Additional file 3: Appendices I to VI in the Additional file 3 show the raw data and supporting information for the various experiments and analysis steps. [file 13072_2019_305_MOESM3_ESM.pdf]

APPENDIX I

Blots as presented in Figure 2

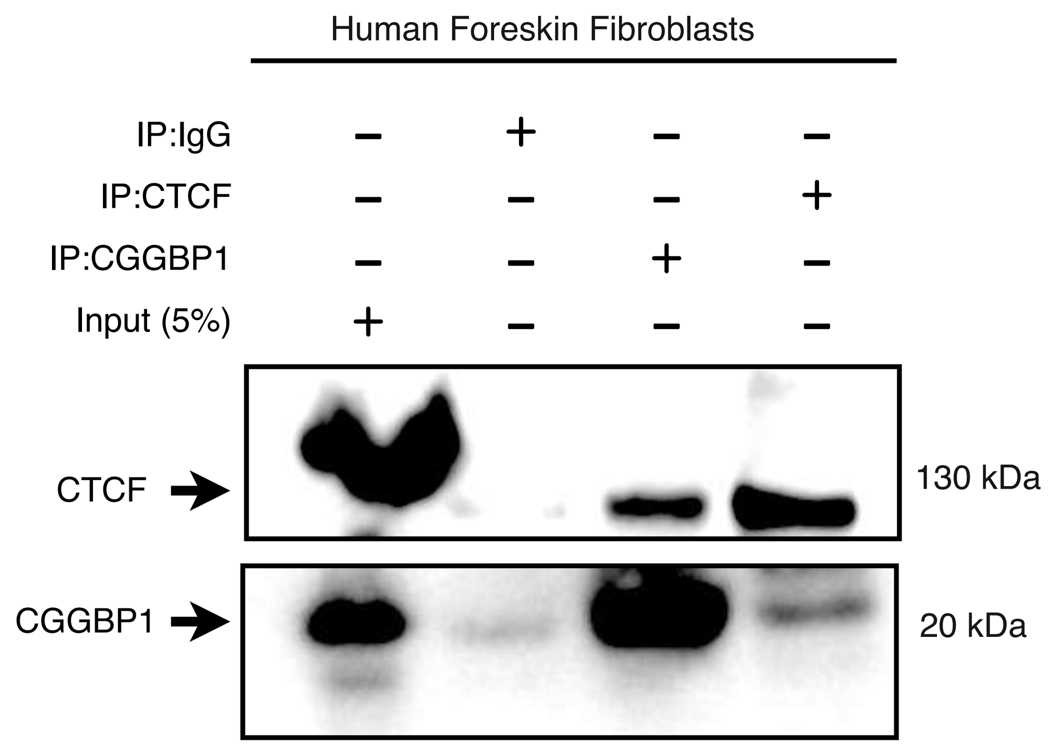

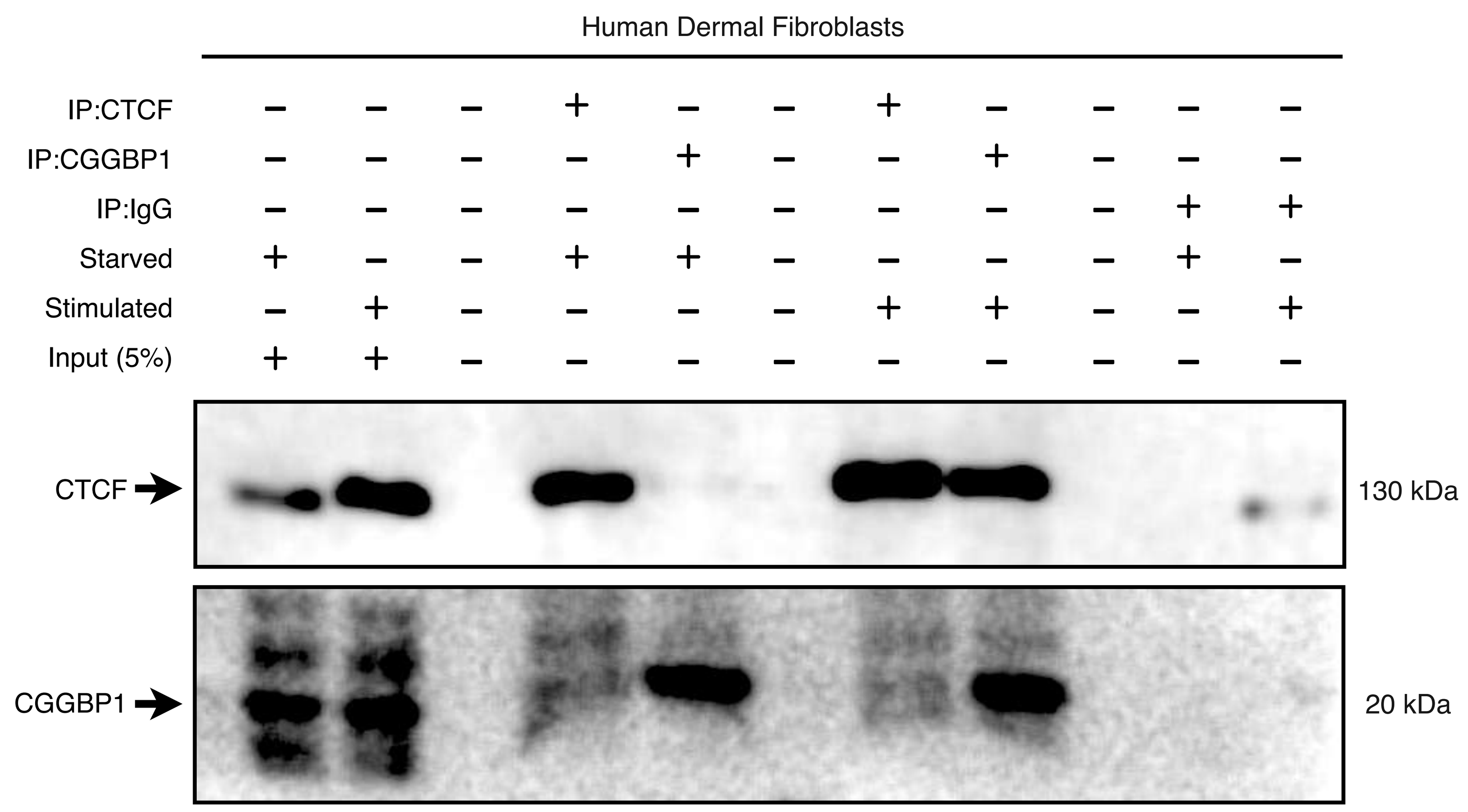

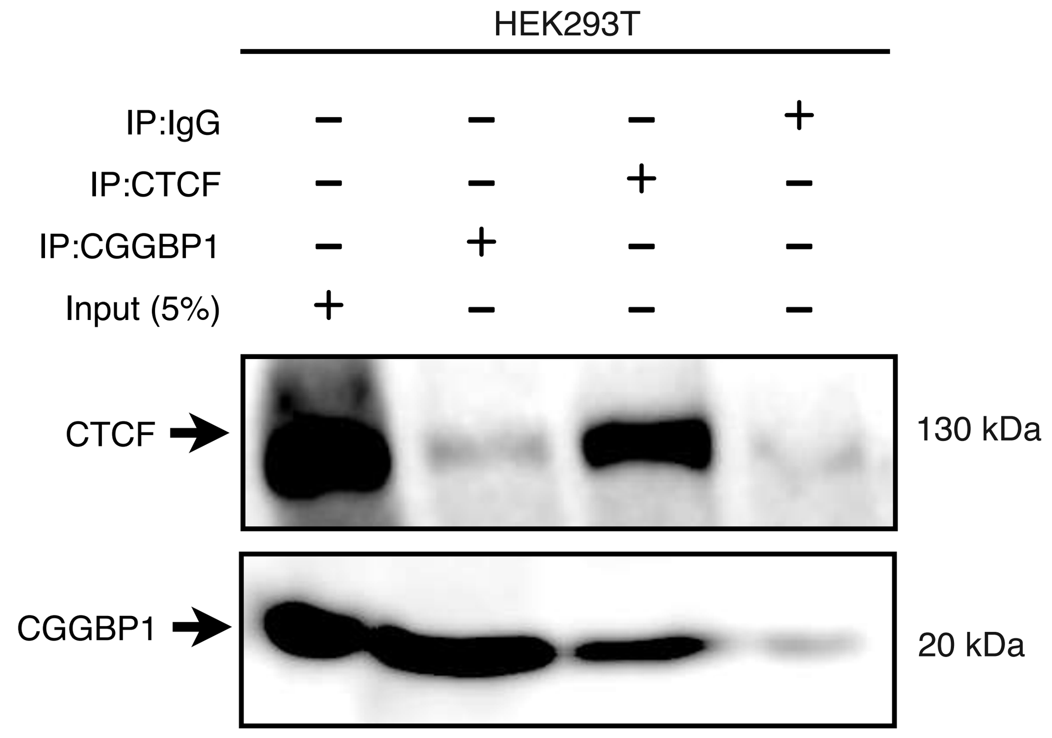

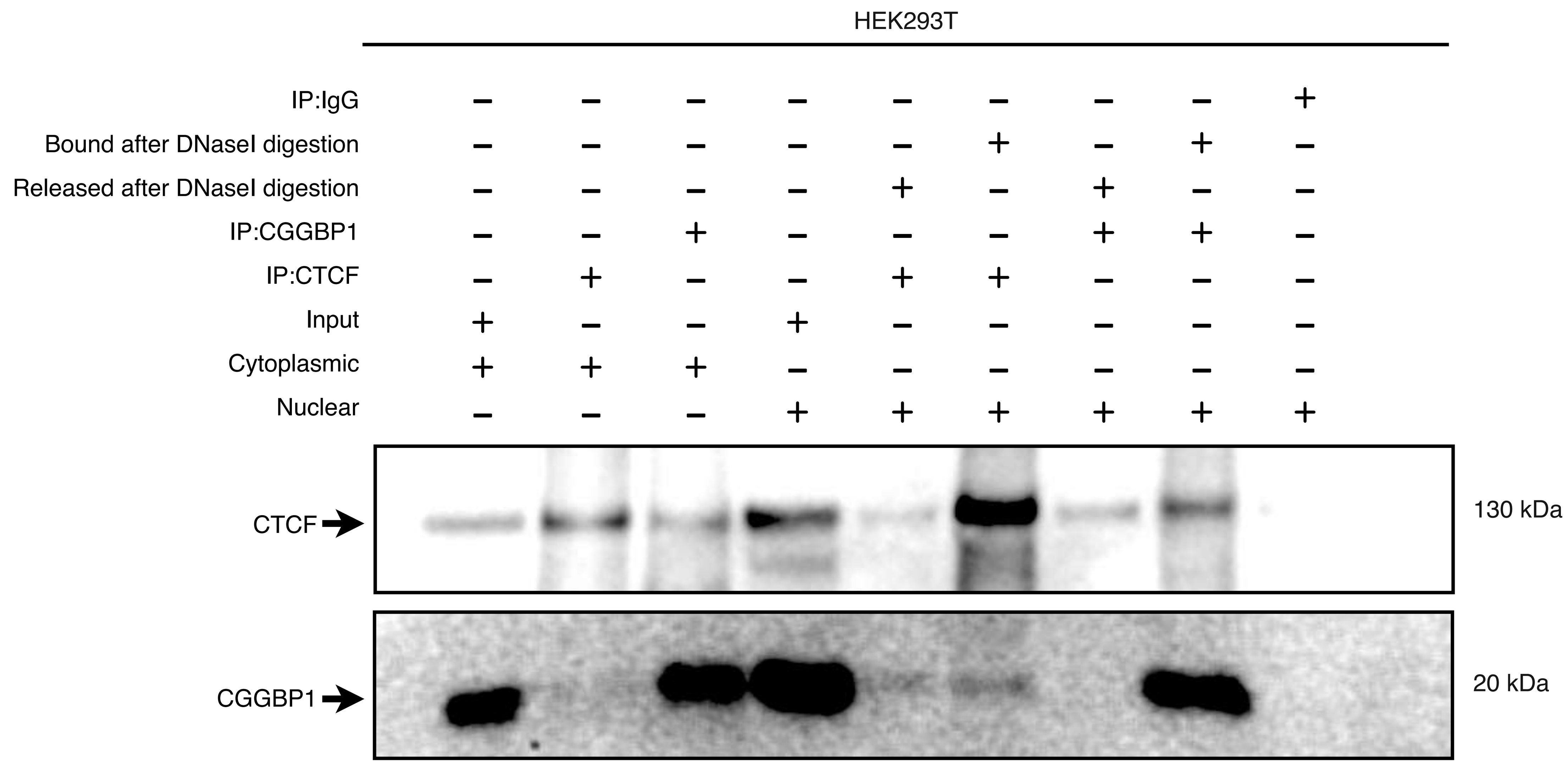

Figure 2A (CTCF)

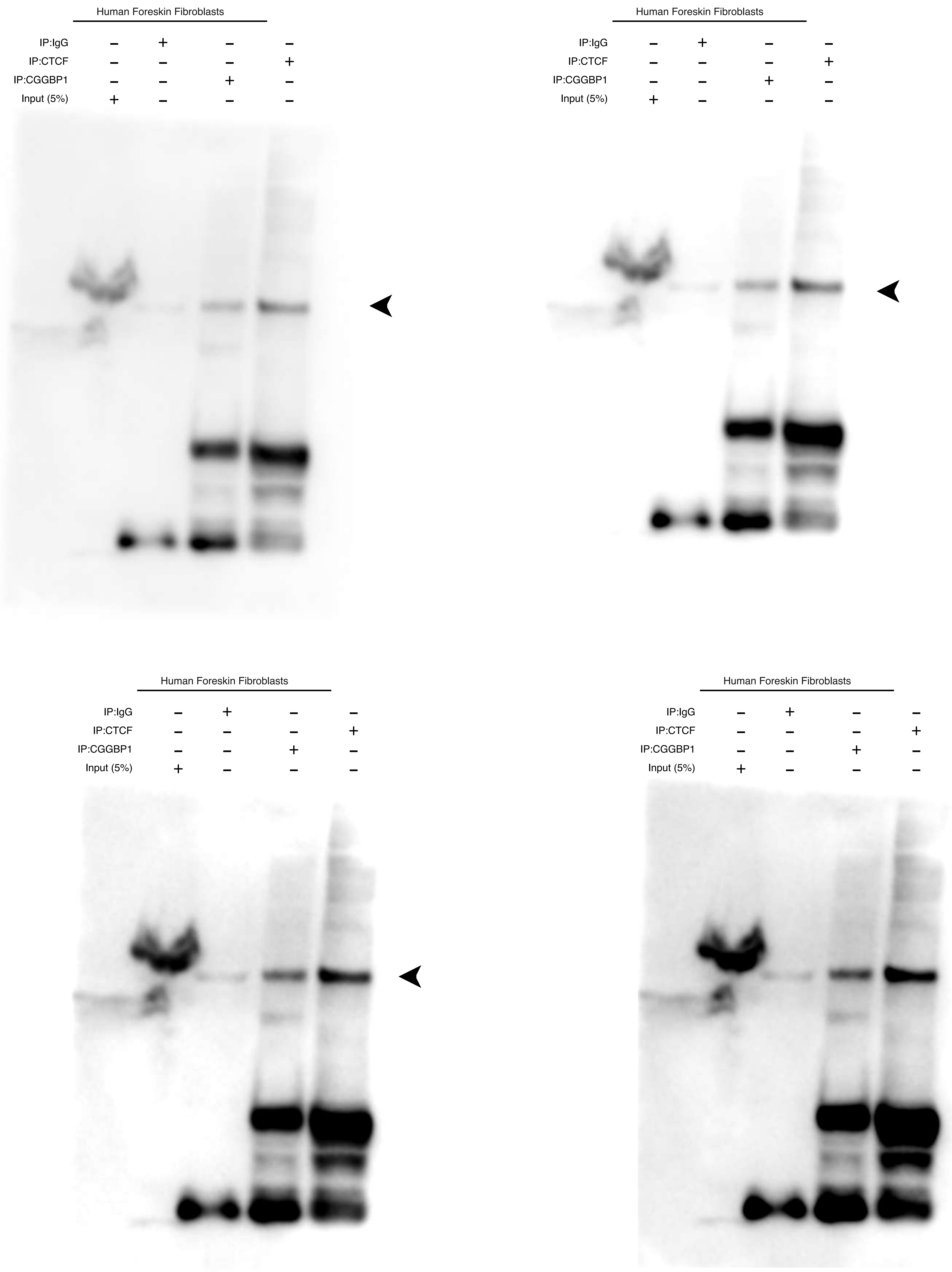

Figure 2A (CGGBP1)

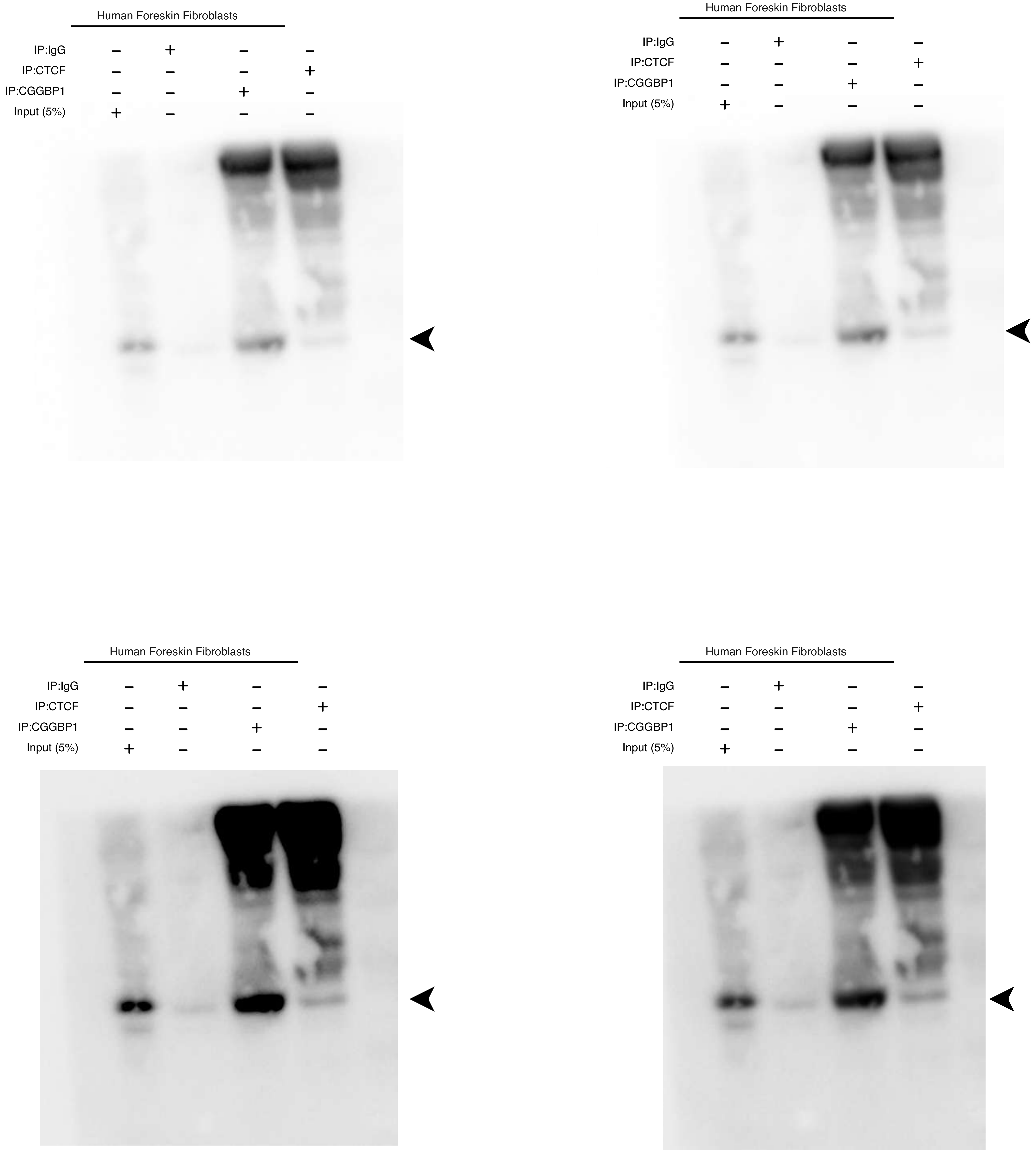

Figure 2B (CTCF)

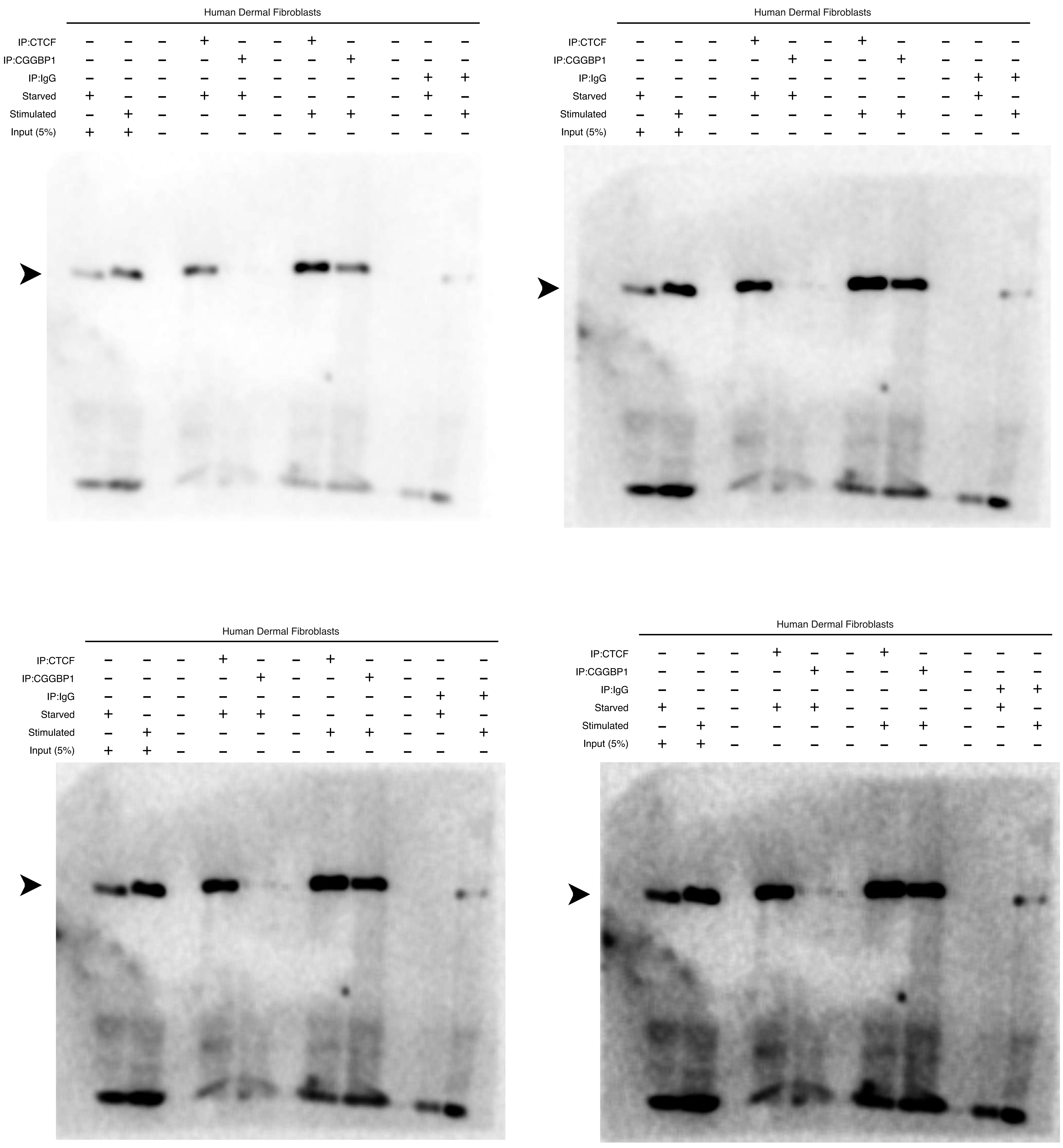

Figure 2B (CGGBP1)

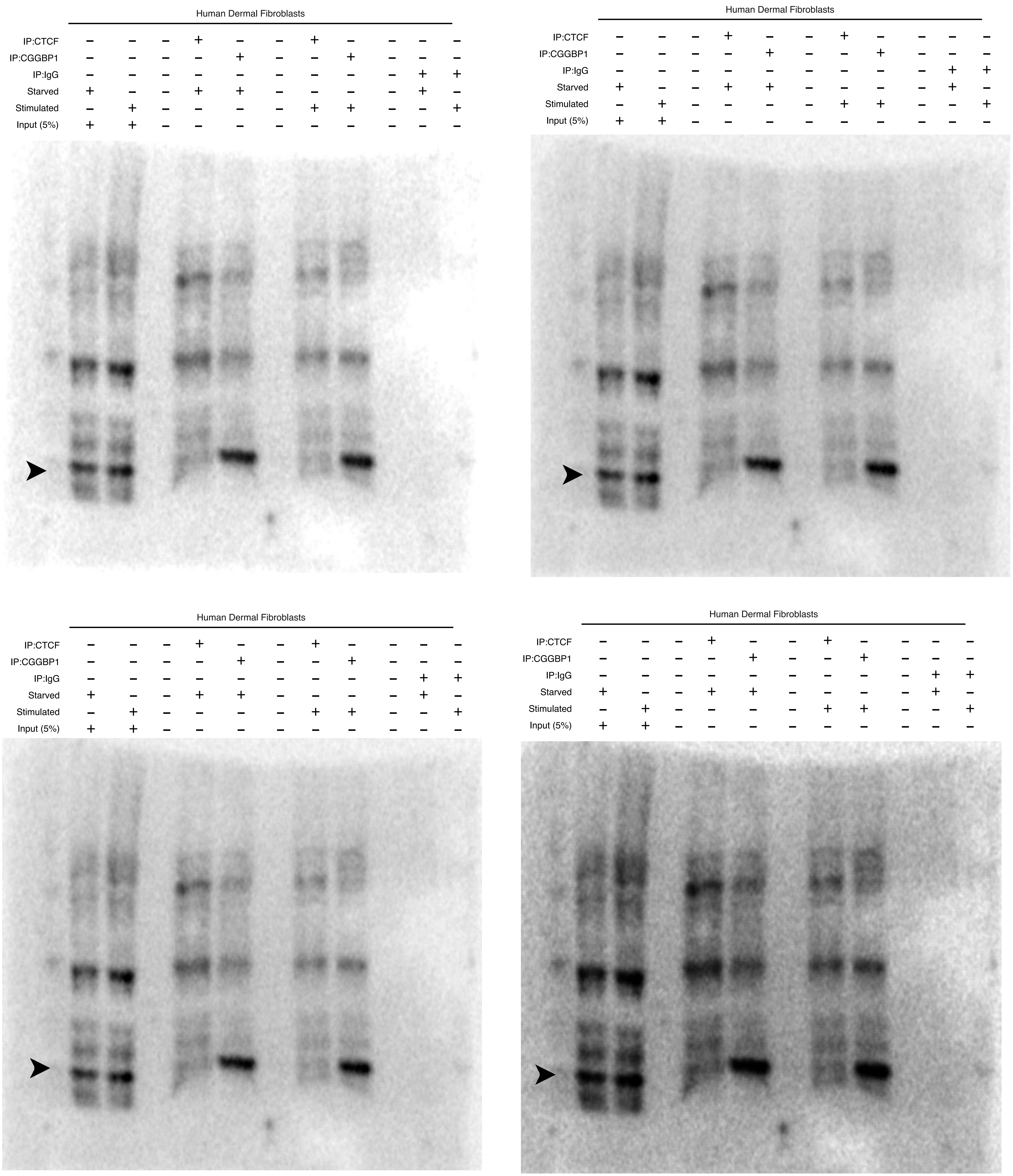

Figure 2C (CTCF)

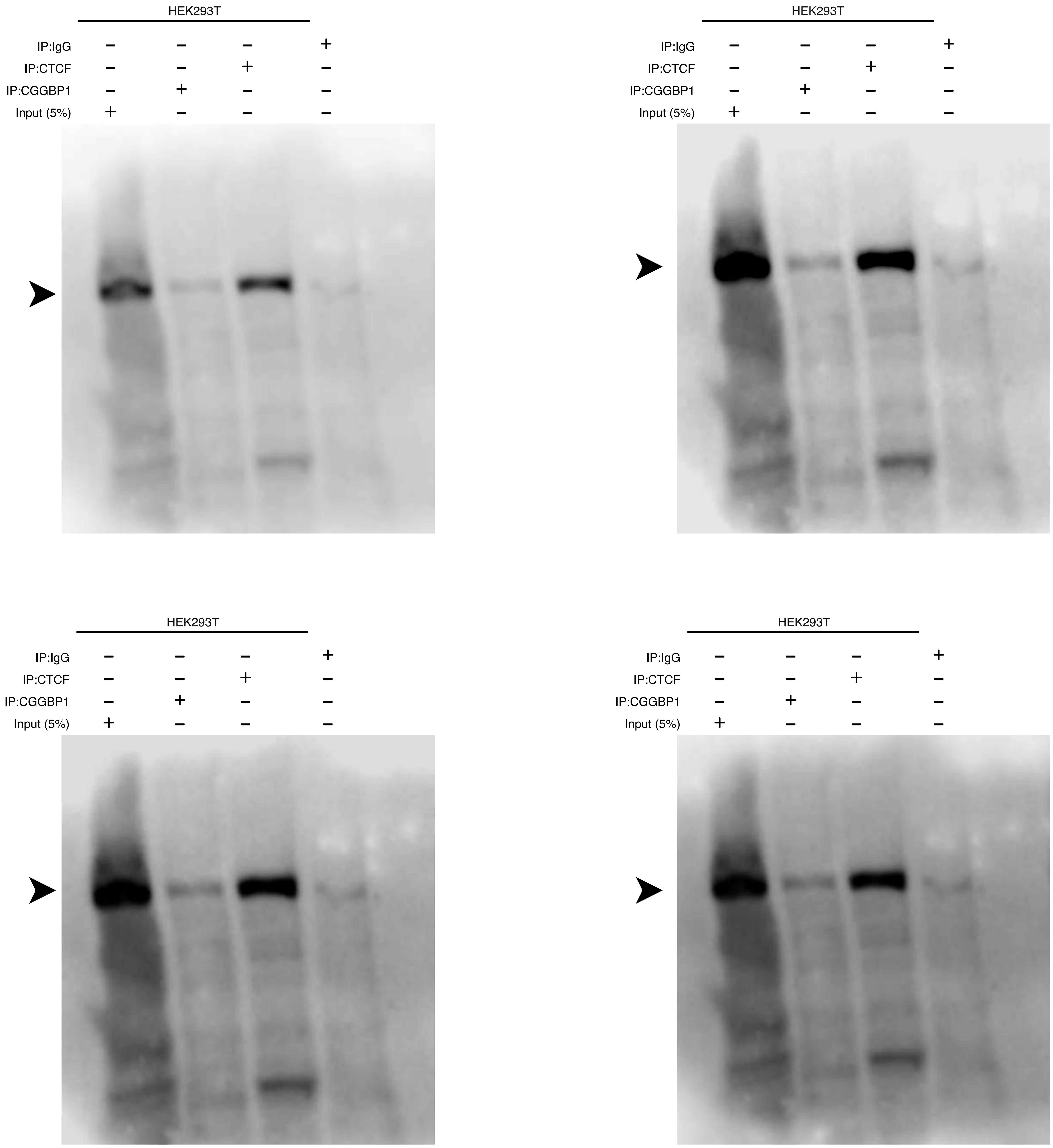

Figure 2C (CGGBP1)

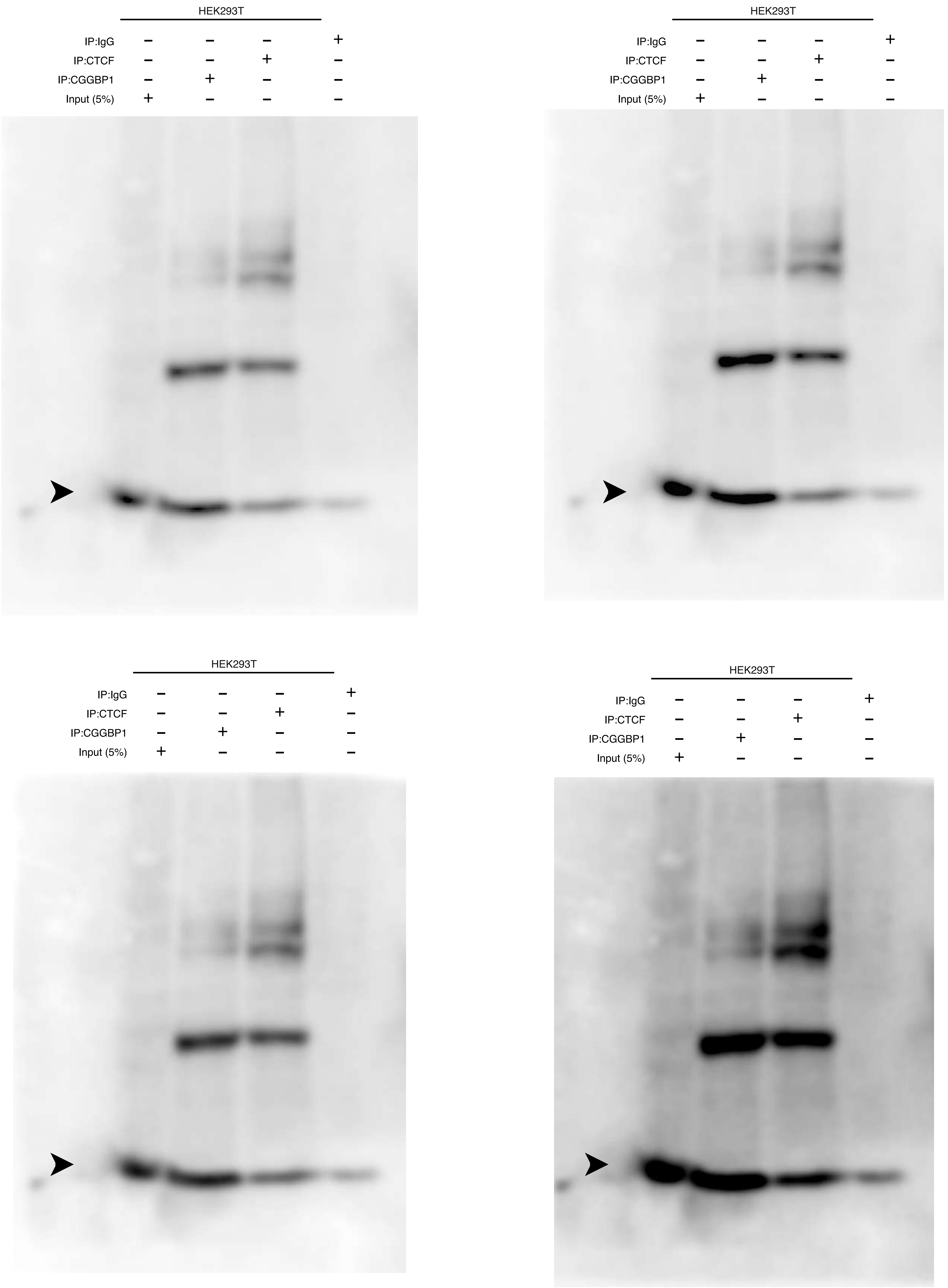

Figure 2D (CTCF)

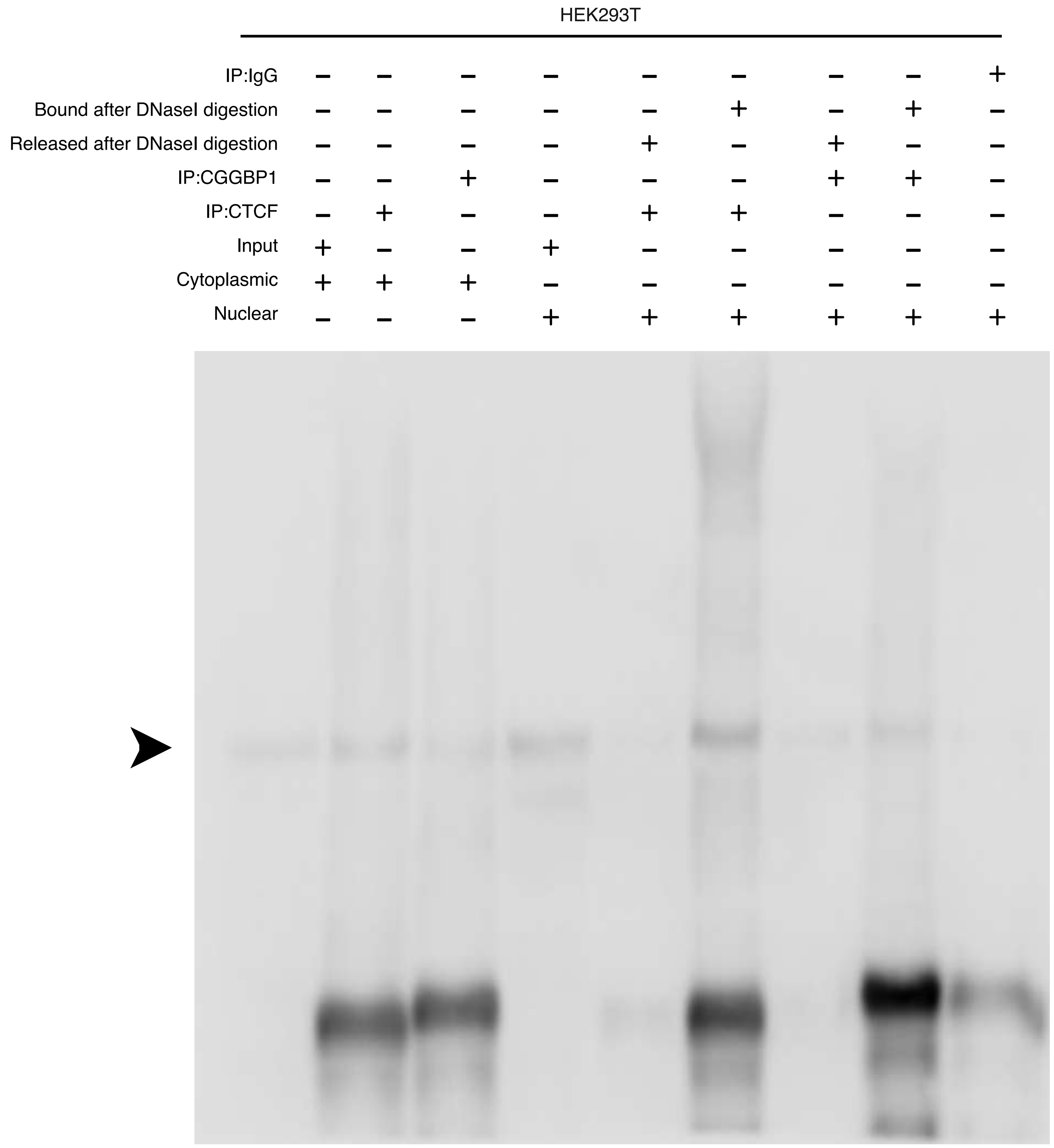

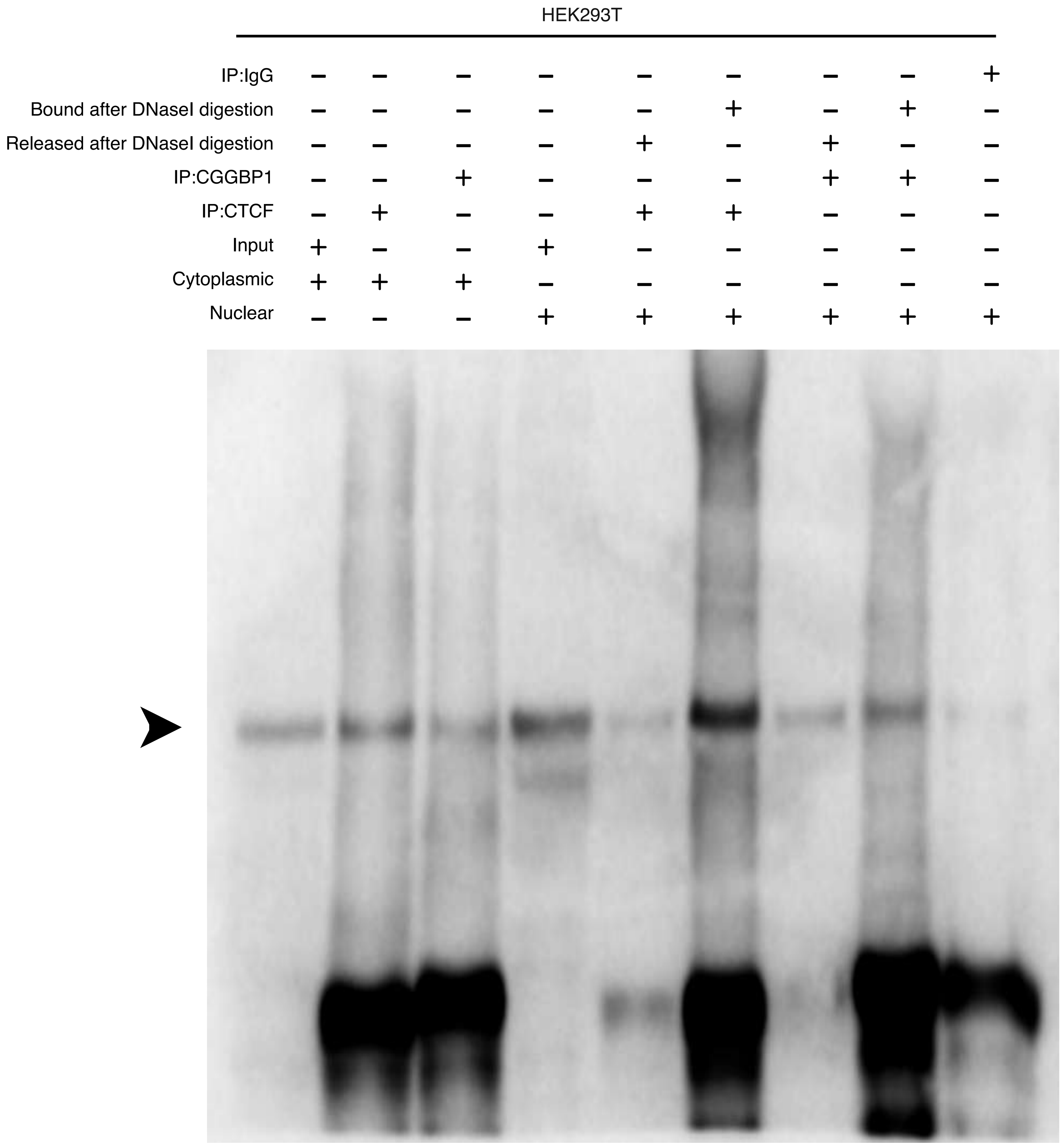

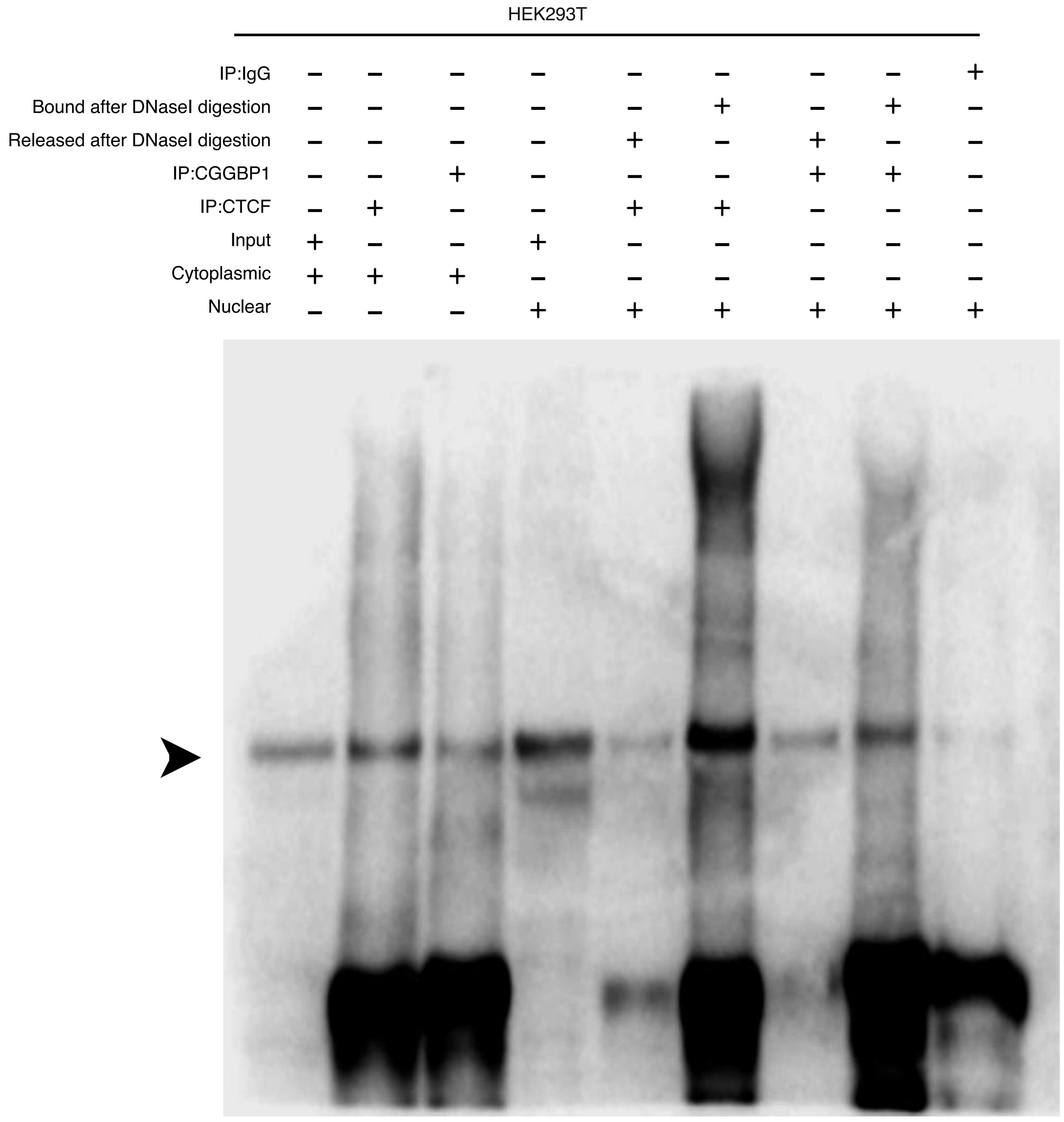

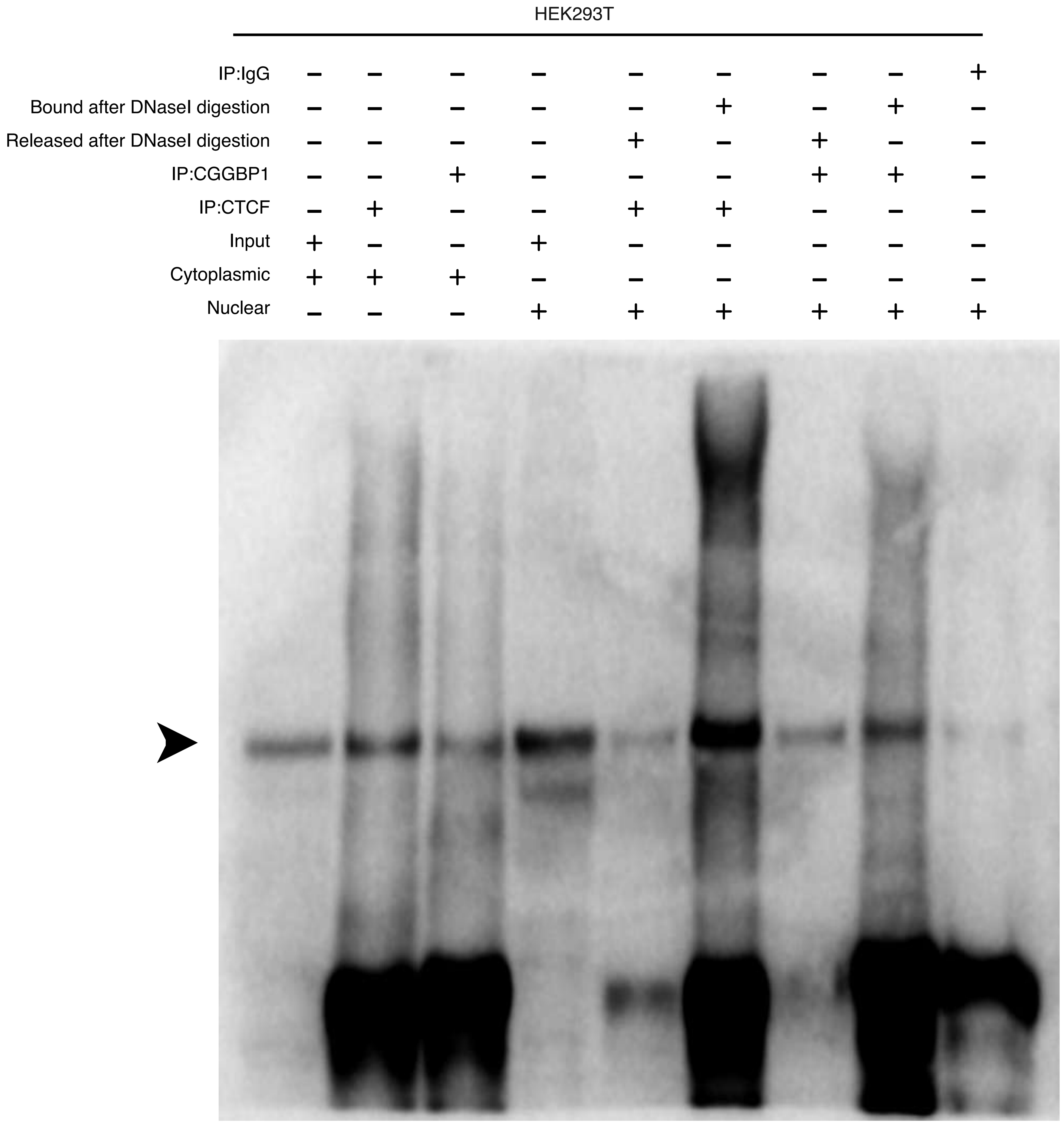

Figure 2D (CGGBP1)

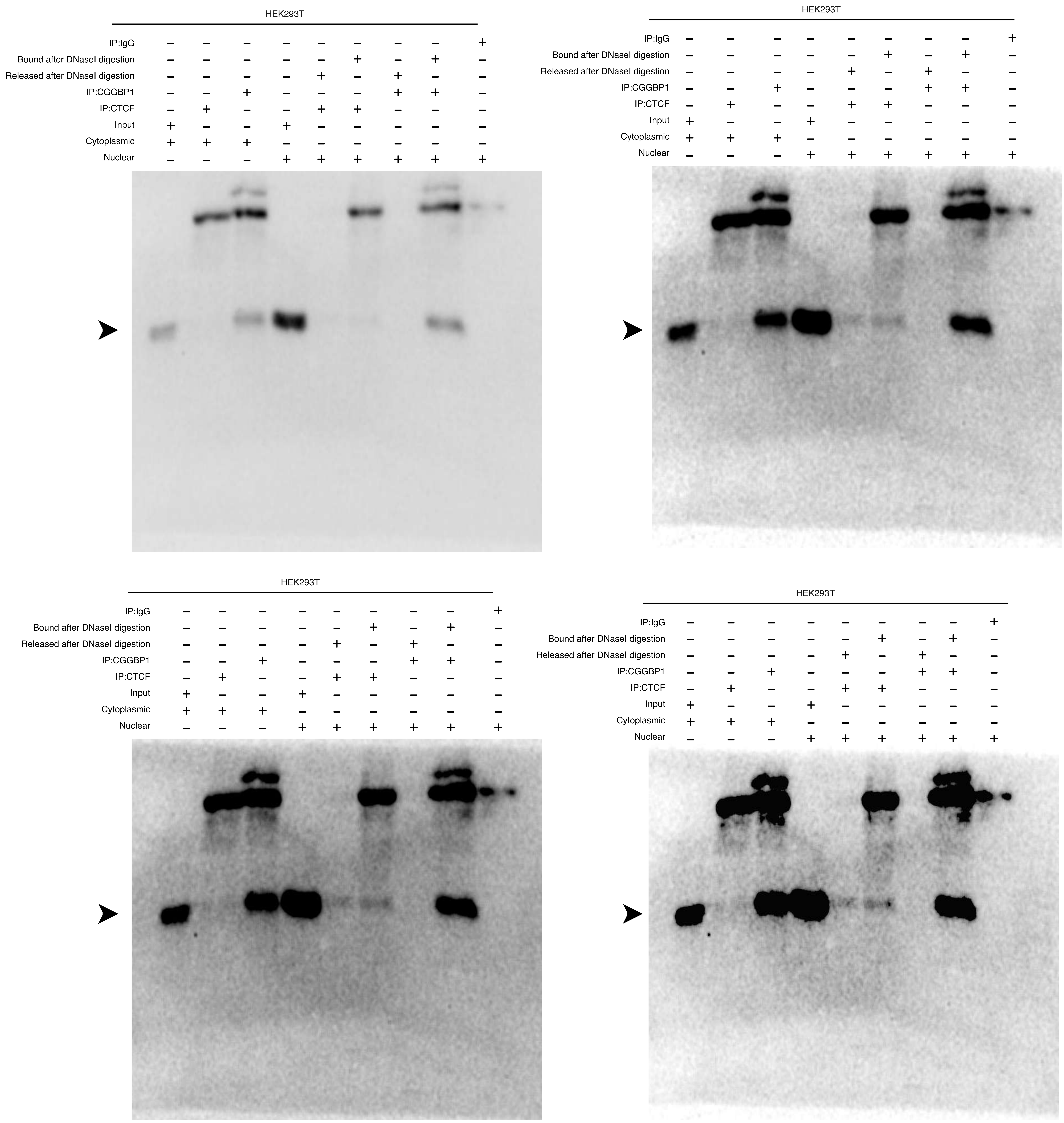

APPENDIX II

Genome-browser views of CTCF ChIP-seq reads along with the published CTCF ChIP-seq datasets

A

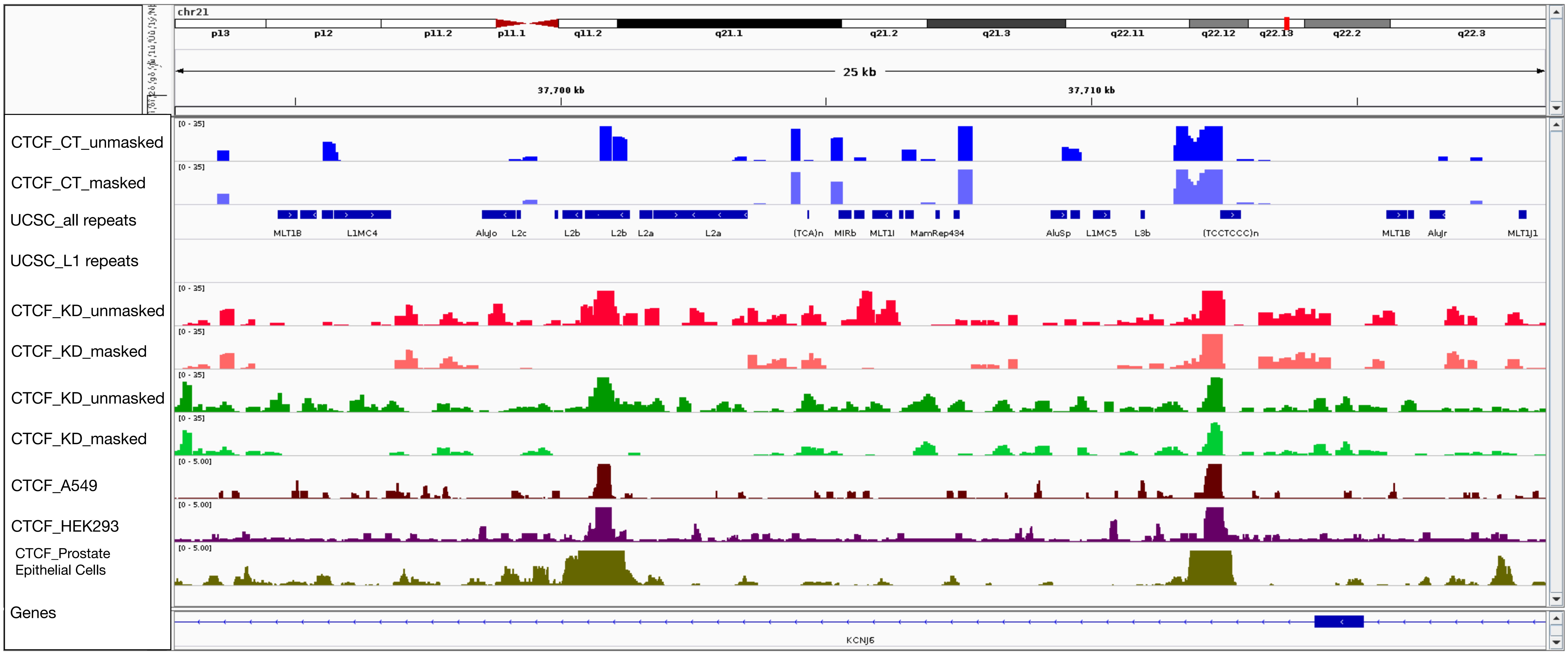

B

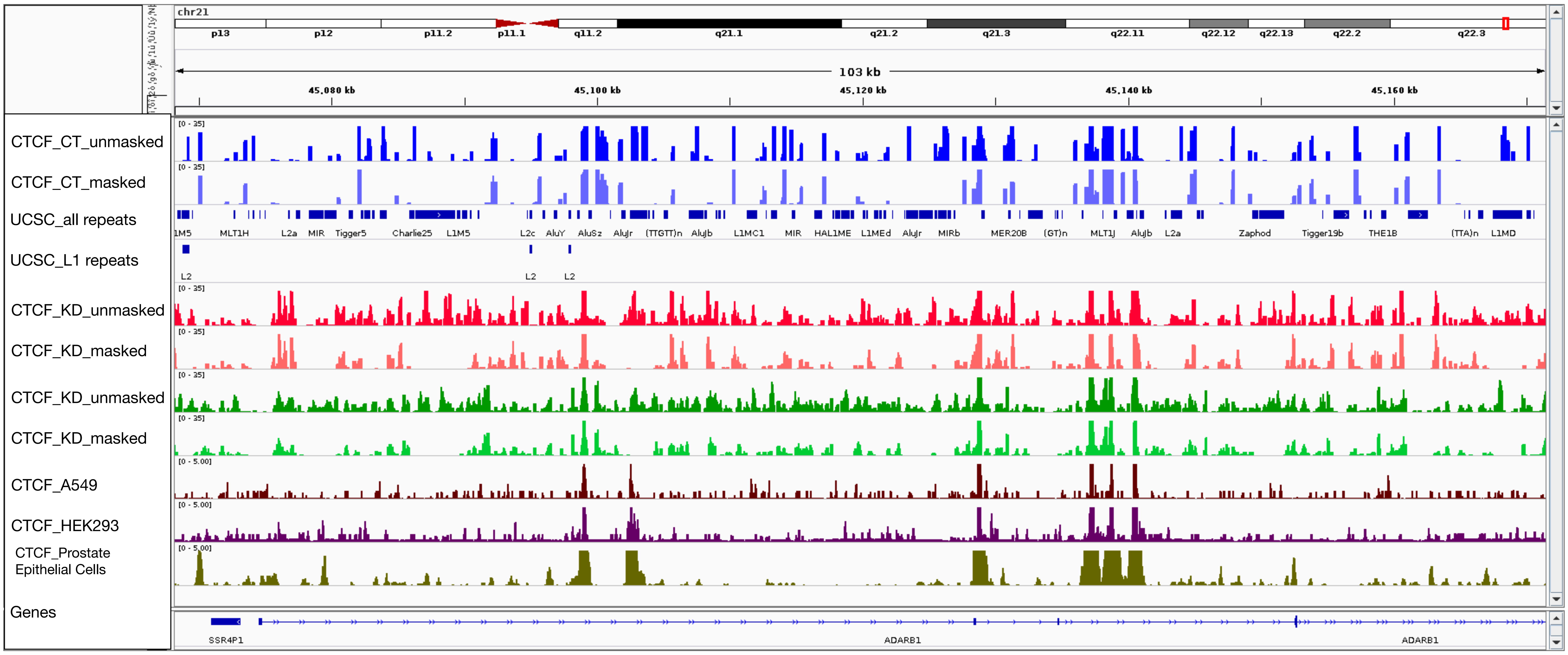

Genome-browser views of CTCF ChIP-seq reads along with the published CTCF ChIP-seq datasets

C

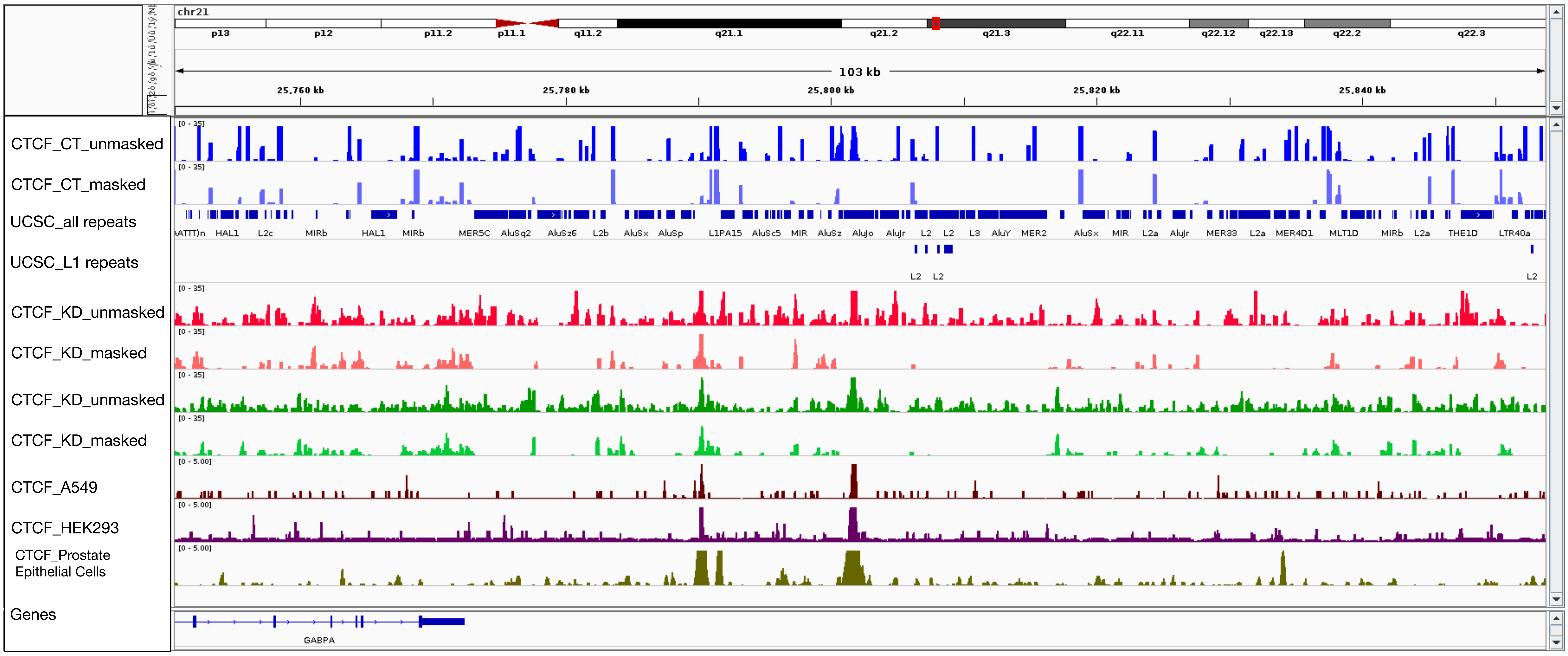

D

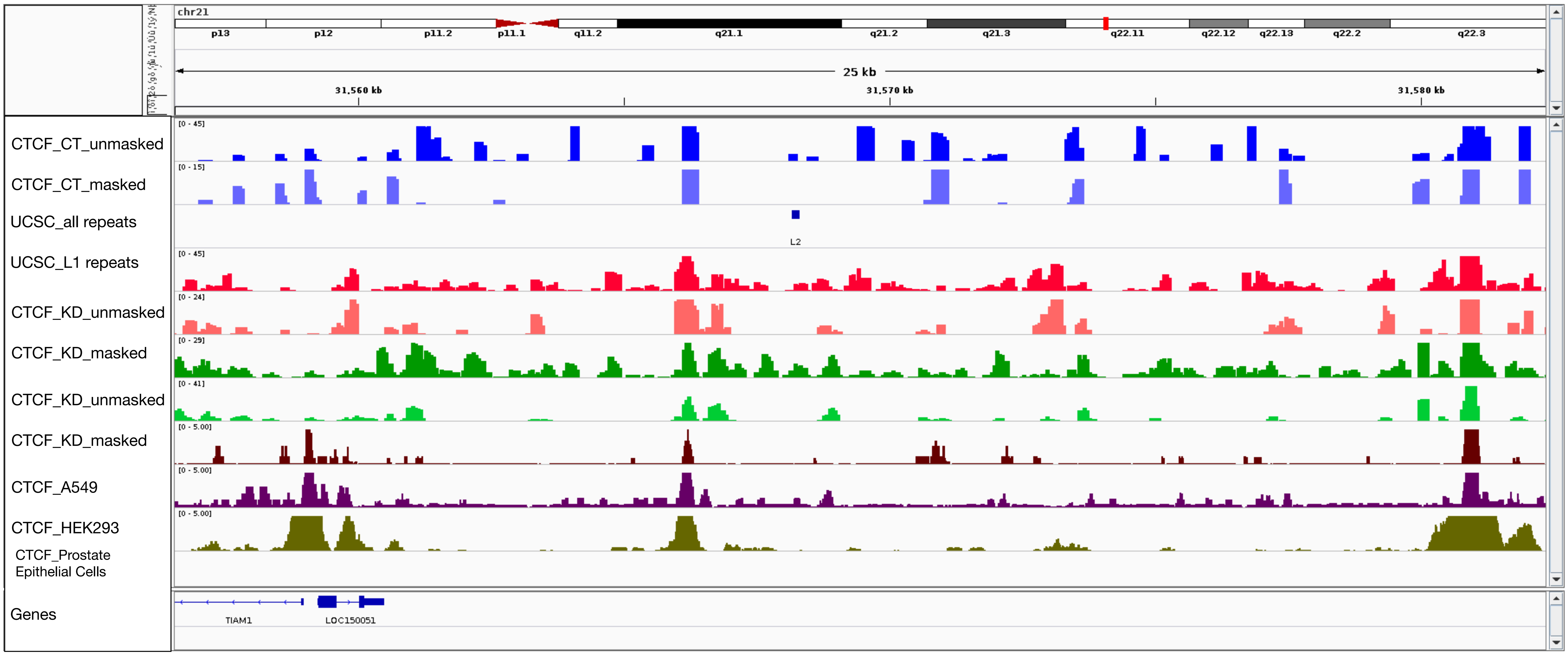

APPENDIX III

Genome-browser views displaying H3K9me3 read distribution patterns

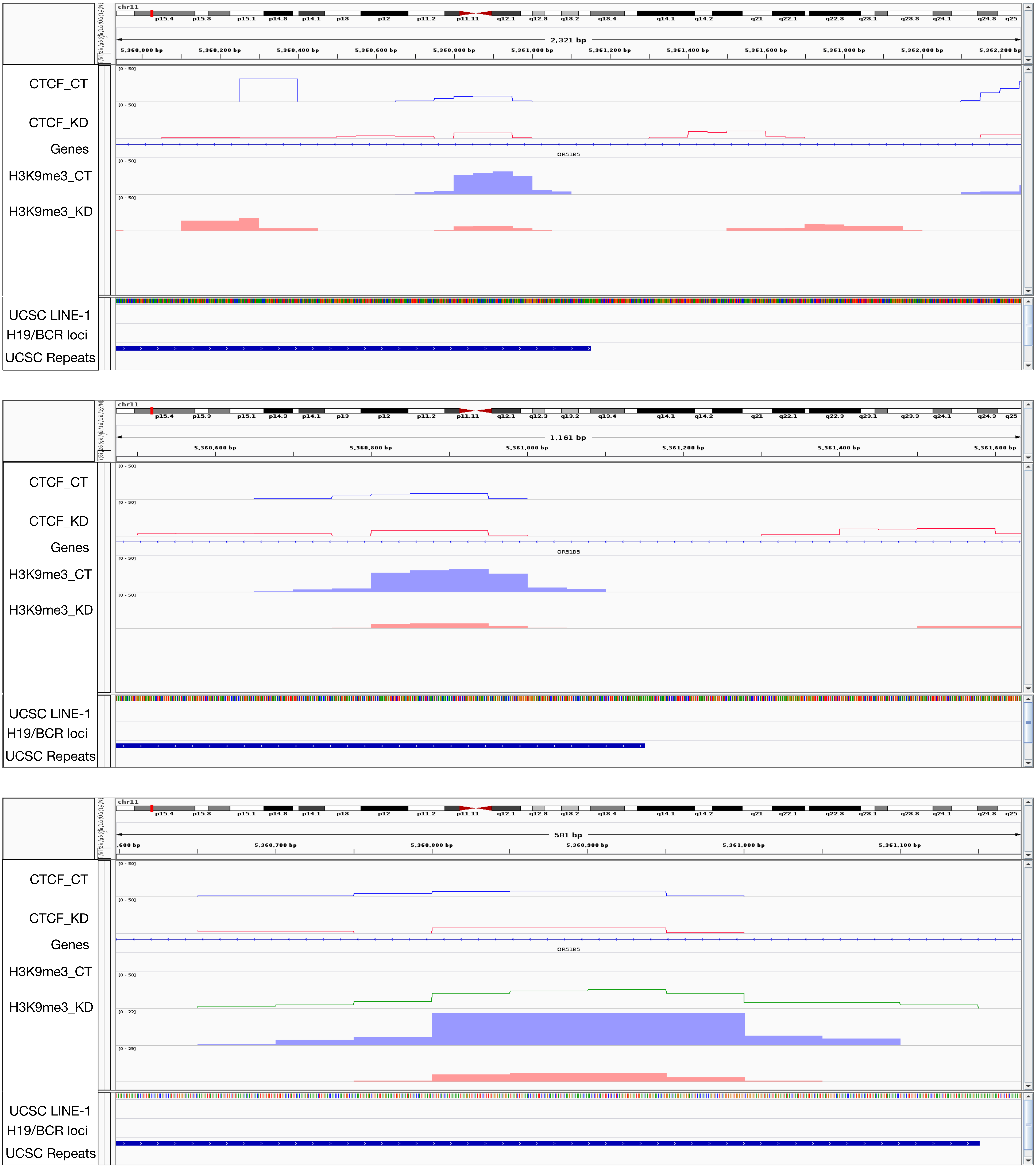

Genome-browser views displaying H3K9me3 read distribution patterns

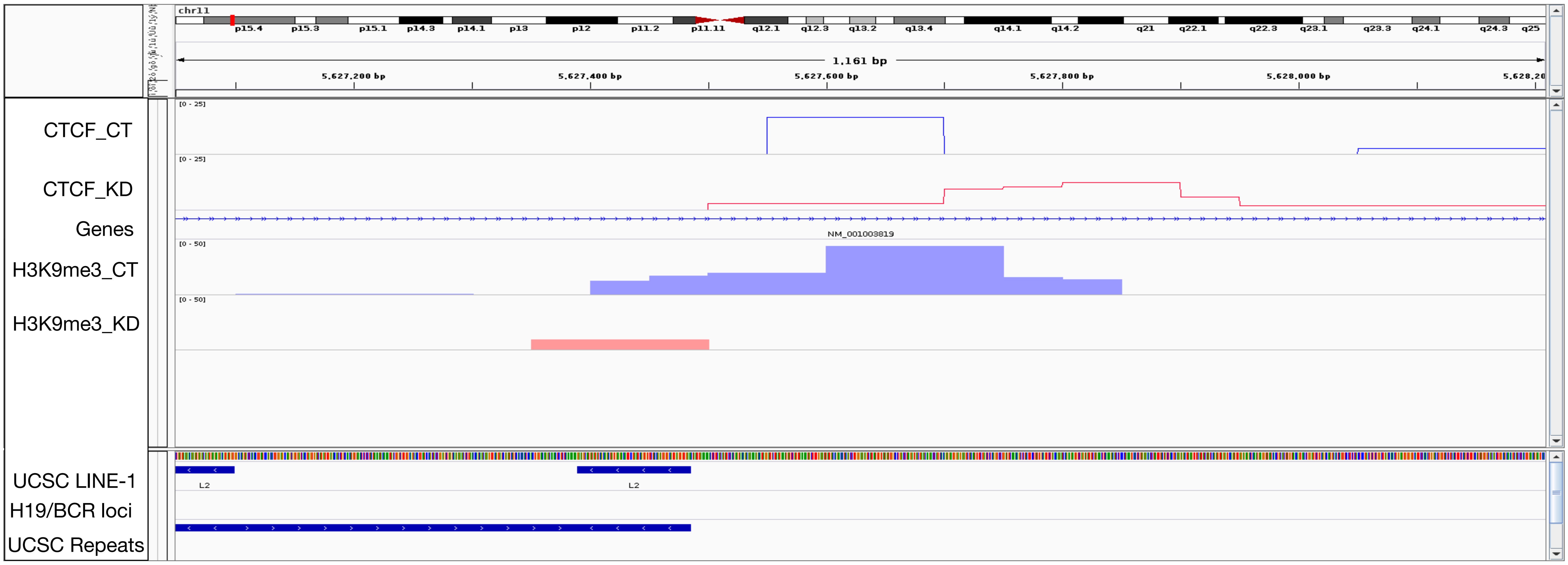

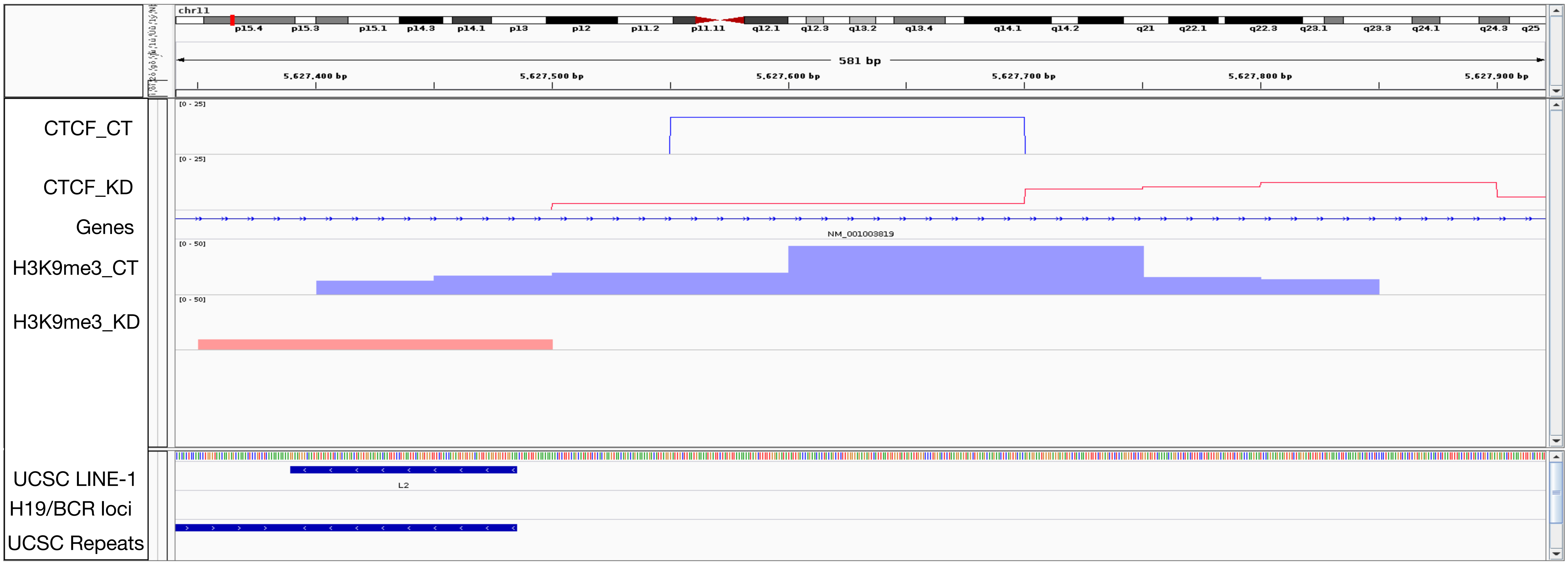

Genome-browser views displaying H3K9me3 read distribution patterns

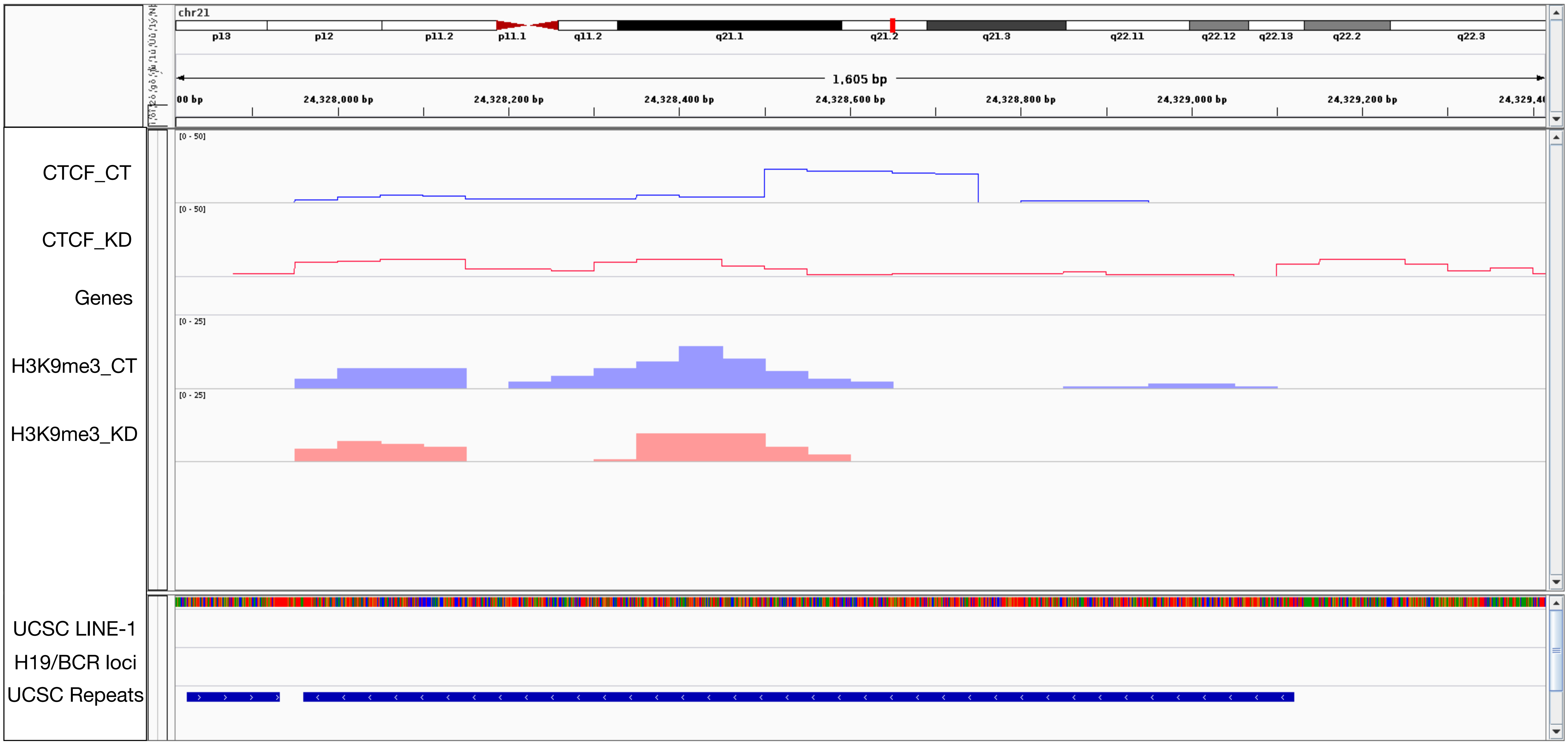

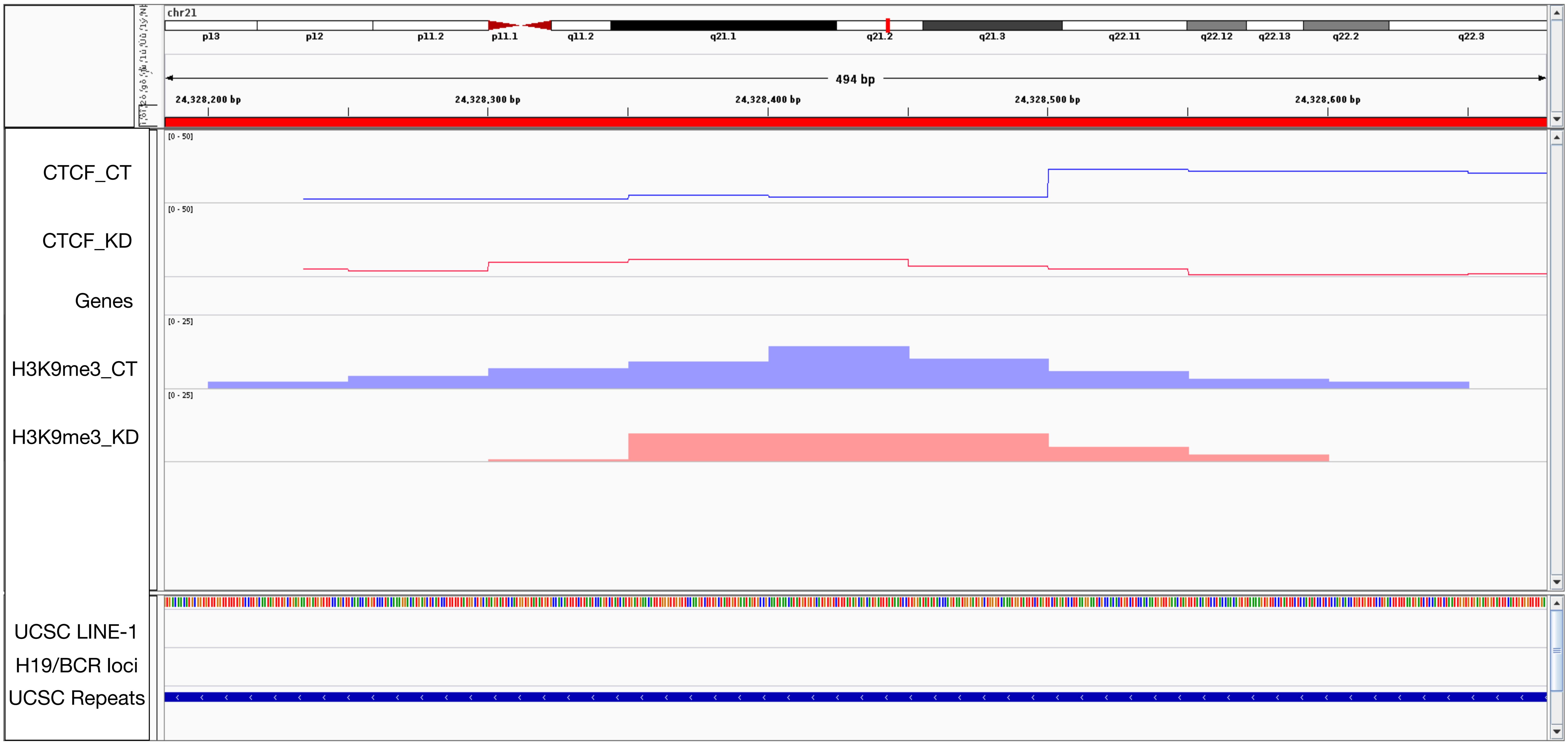

Genome-browser views displaying H3K9me3 read distribution patterns

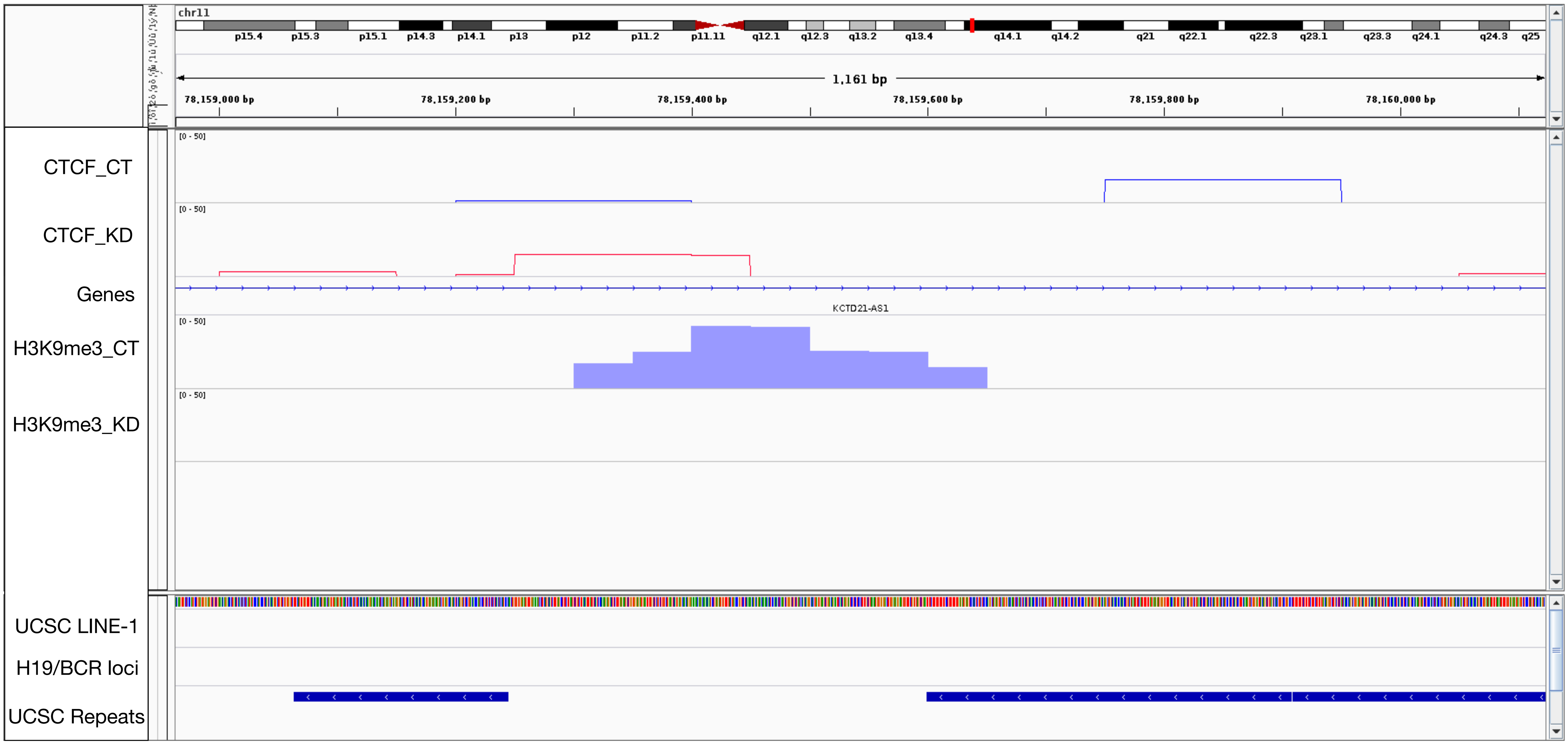

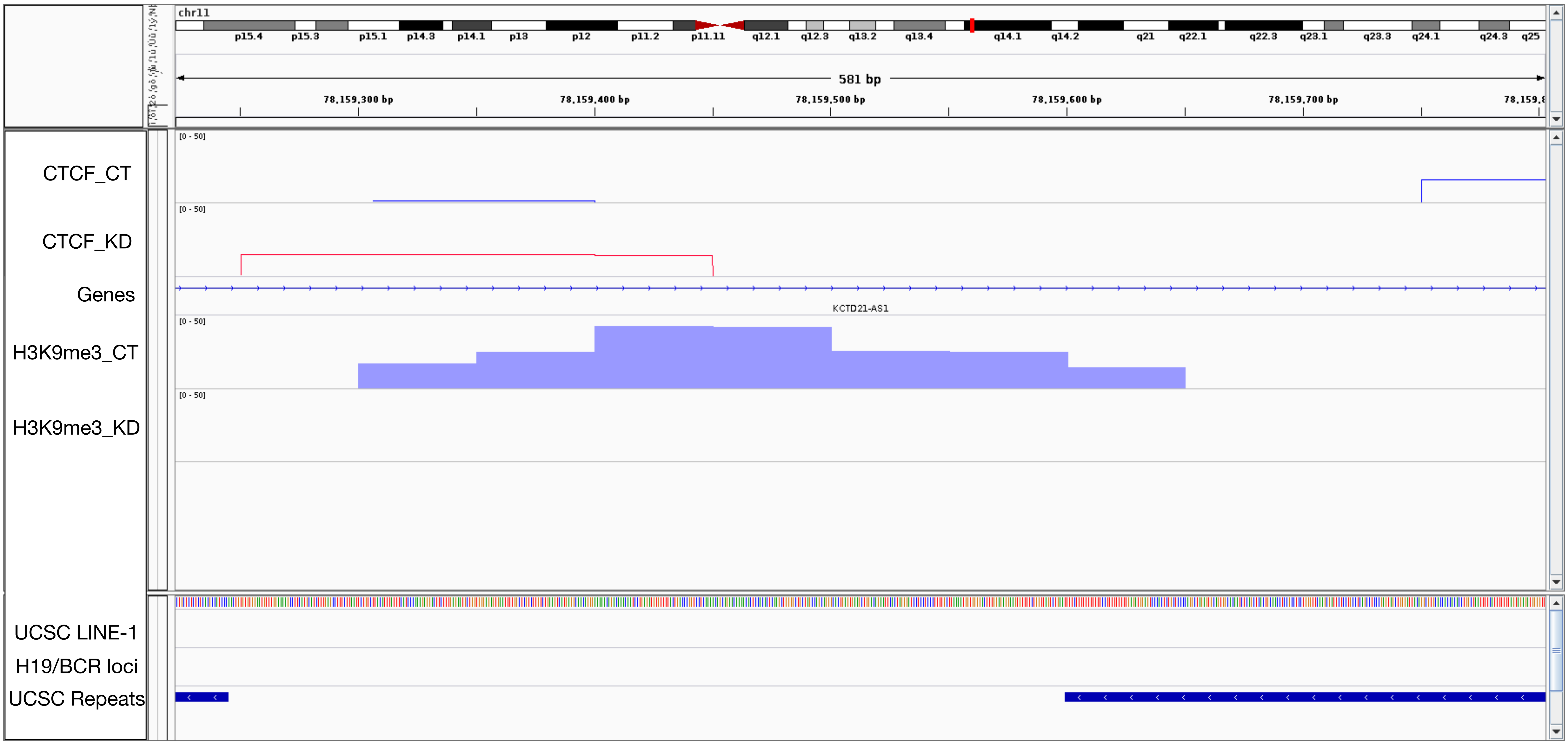

APPENDIX IV

Genome-browser views displaying CTCF read distribution patterns in various regions of Chr21.

The Y-axis range is varying (Autoscale function of IGV). The peaks shown here are all from repeat-masked alignment of reads to hg38.

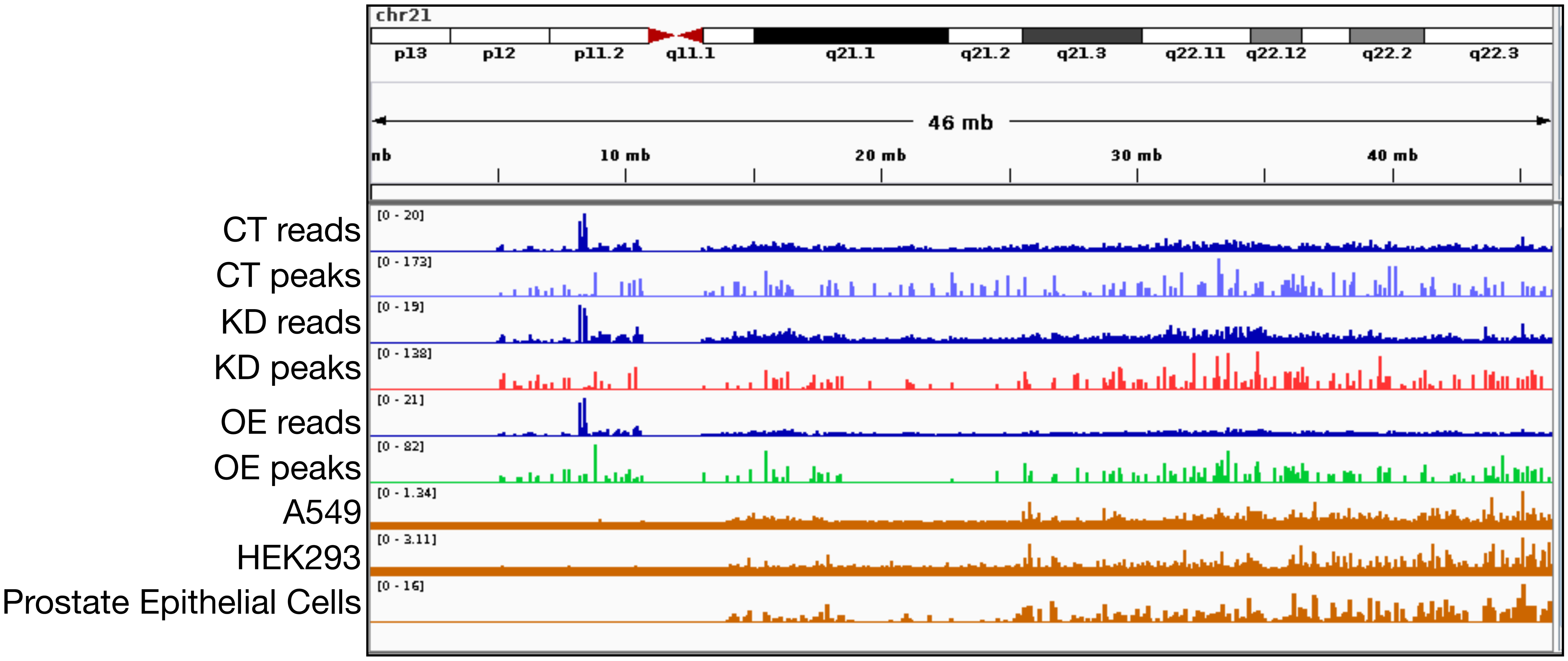

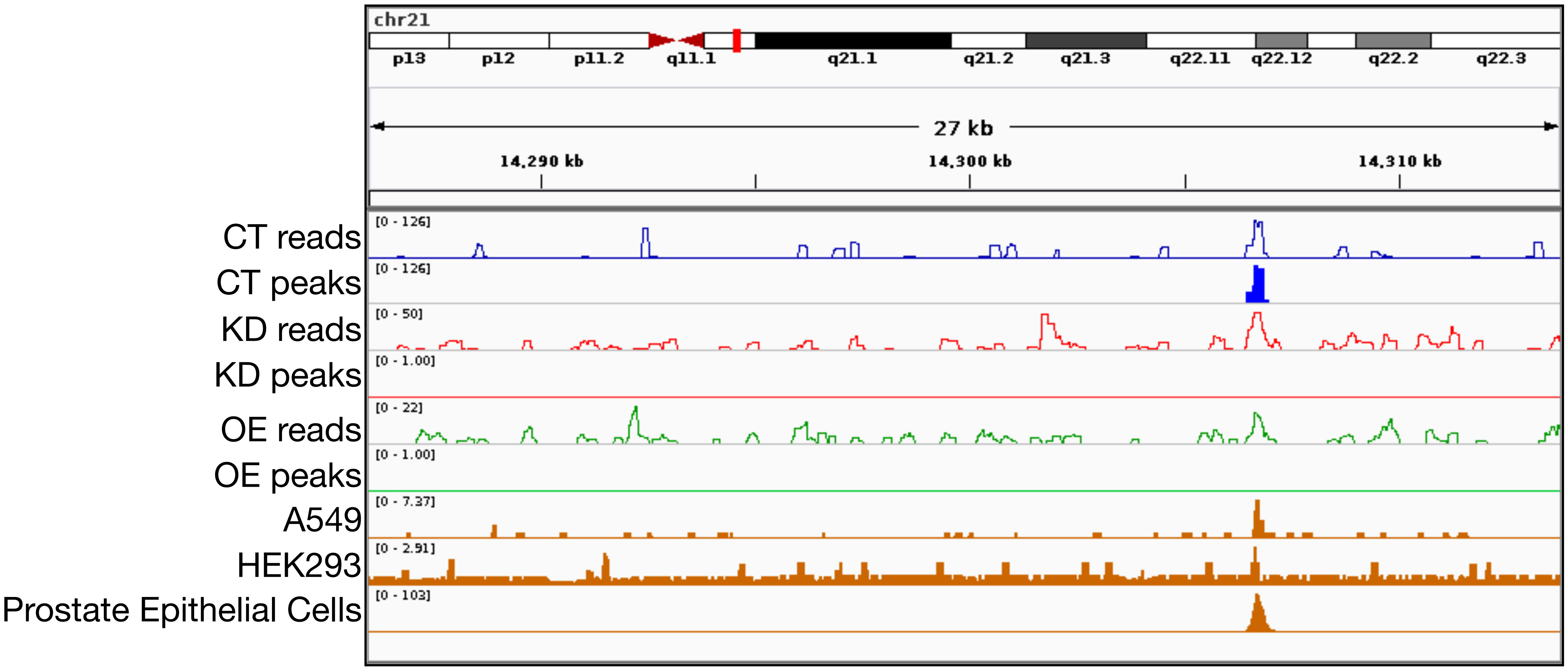

Genome-browser views displaying CTCF read distribution patterns in various regions of Chr21.

The Y-axis range is varying (Autoscale function of IGV). The peaks shown here are all from repeat-masked alignment of reads to hg38.

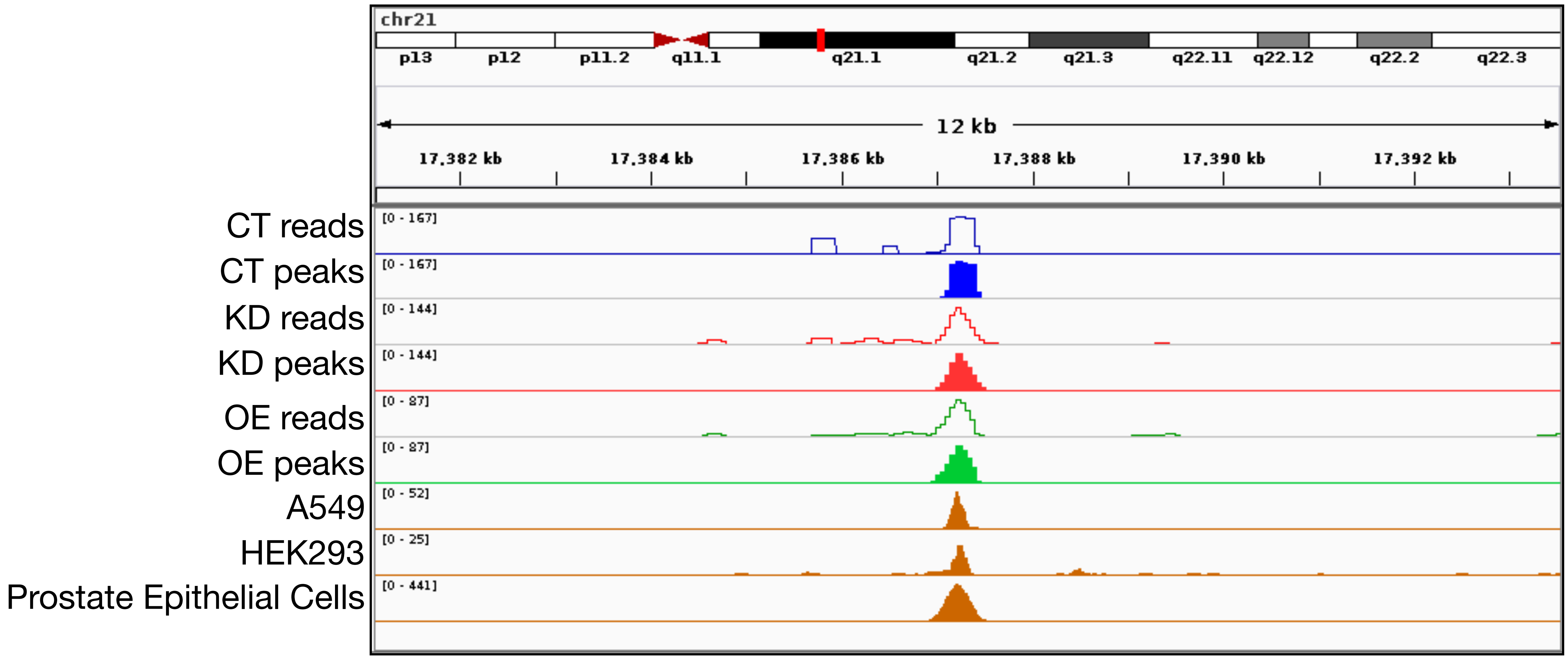

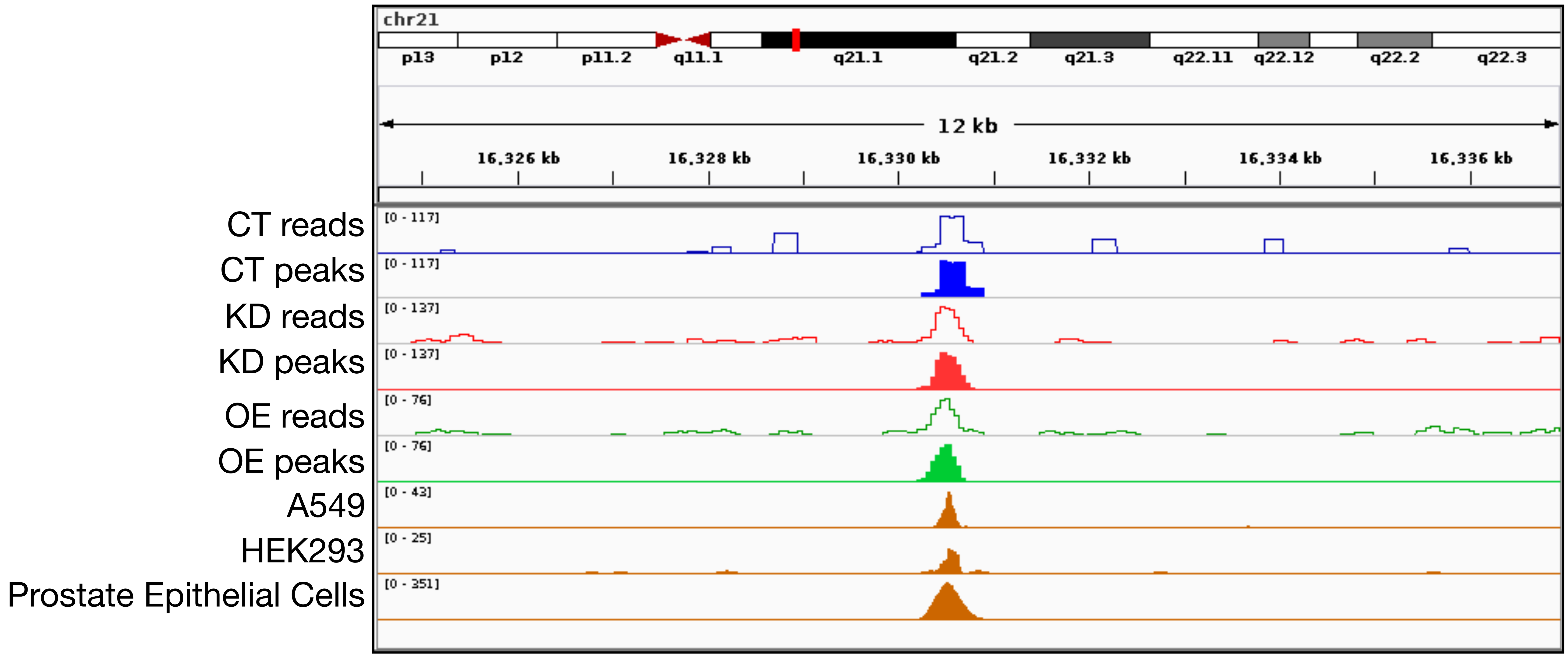

Genome-browser views displaying CTCF read distribution patterns in various regions of Chr21.

The Y-axis range is varying (Autoscale function of IGV). The peaks shown here are all from repeat-masked alignment of reads to hg38.

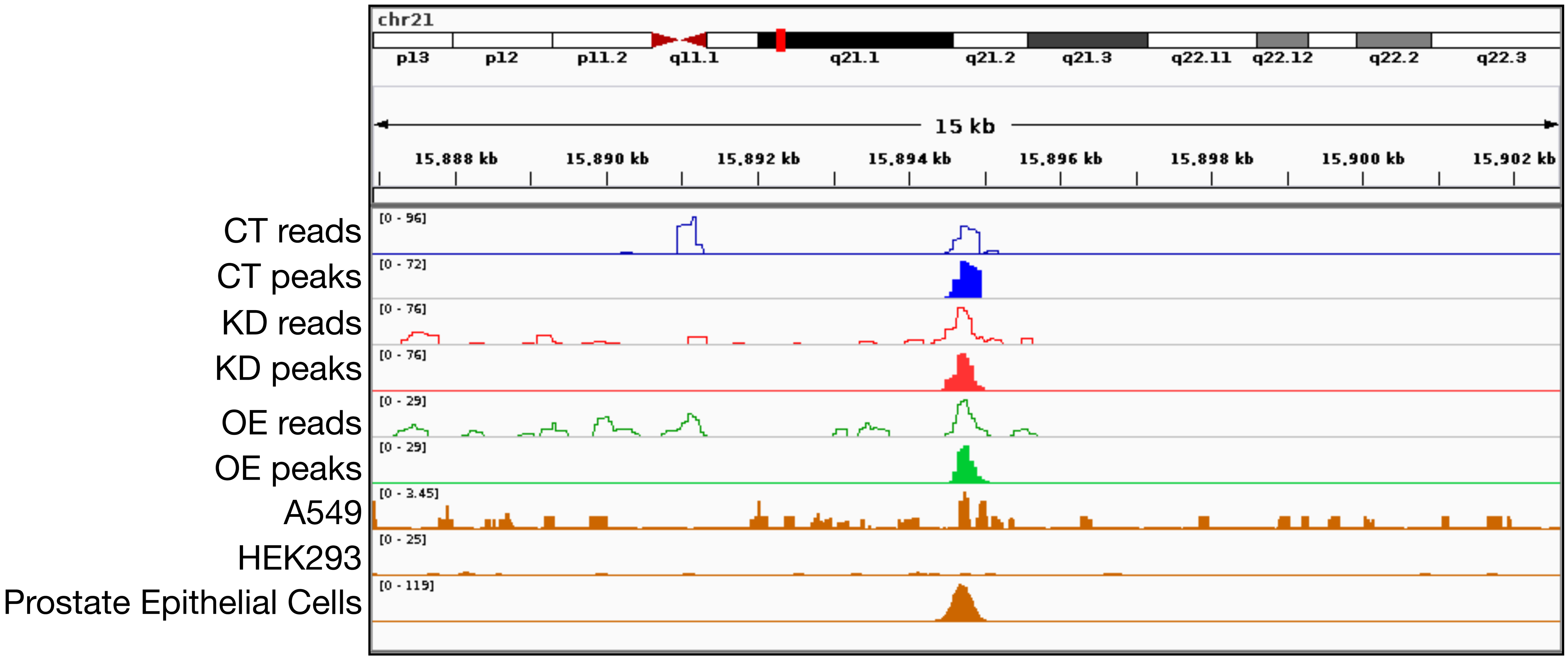

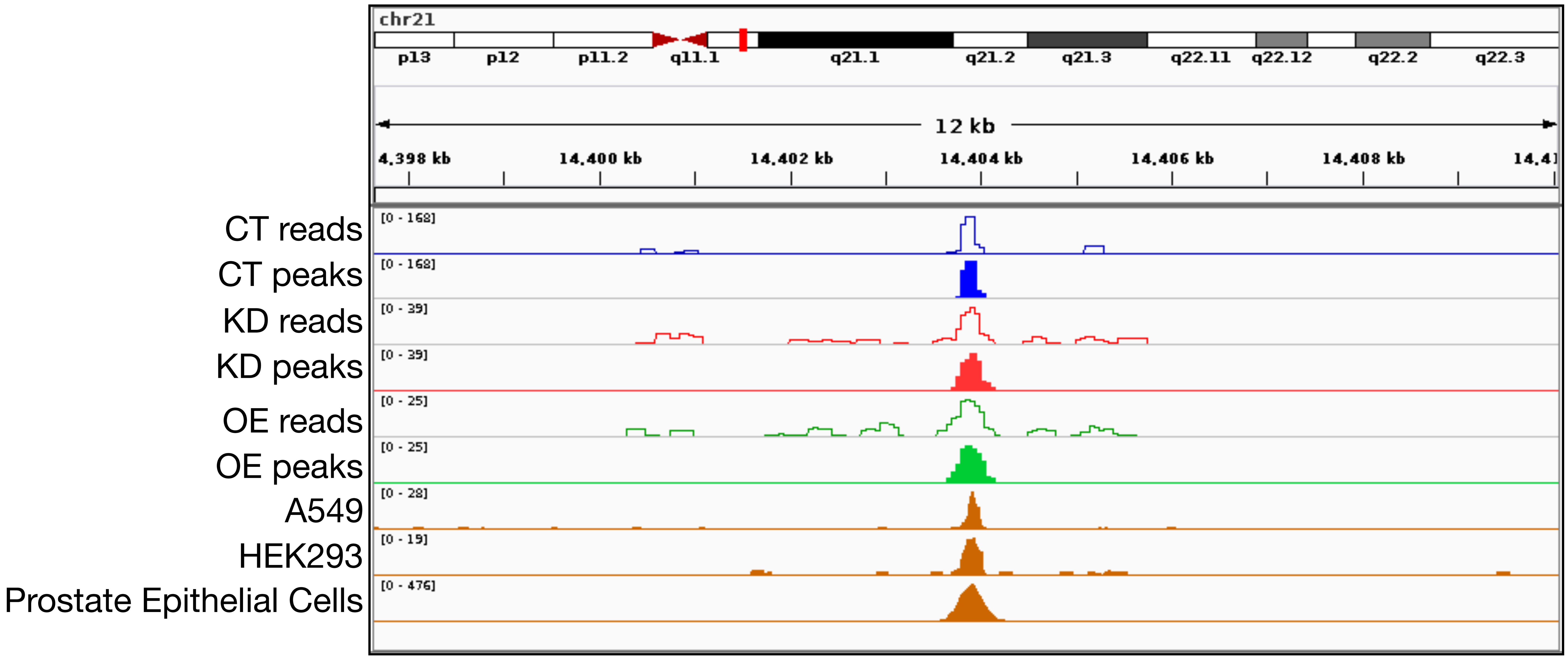

APPENDIX V

CT<br>No. of peaks = 8765		KD<br>No. of peaks = 4775		OE<br>No. of peaks = 5250	
**Motif**	**Count**	**Motif**	**Count**	**Motif**	**Count**
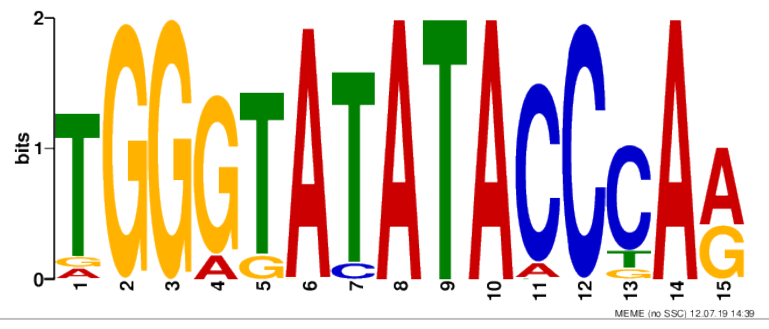	1078	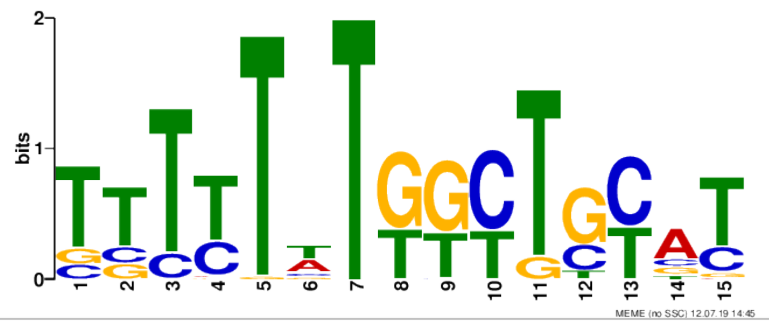	1396	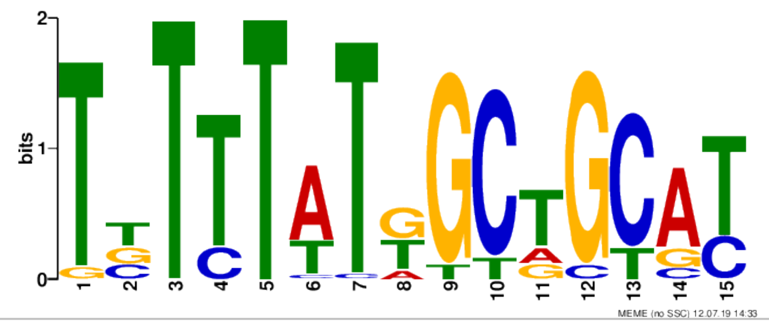	676
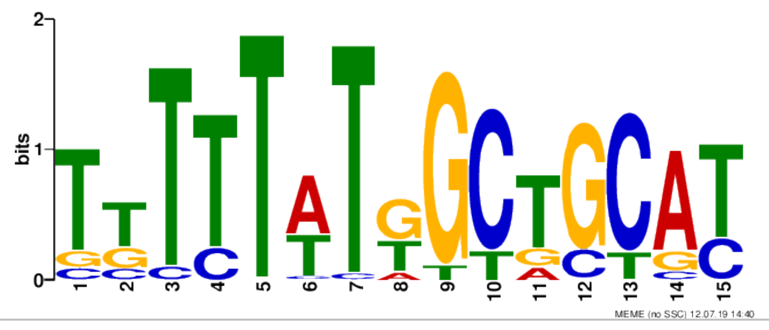	2197	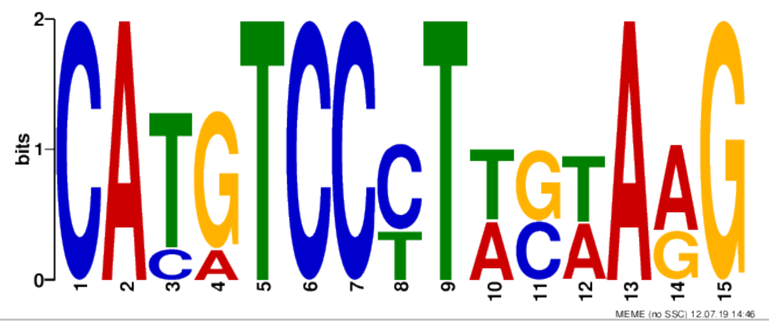	320	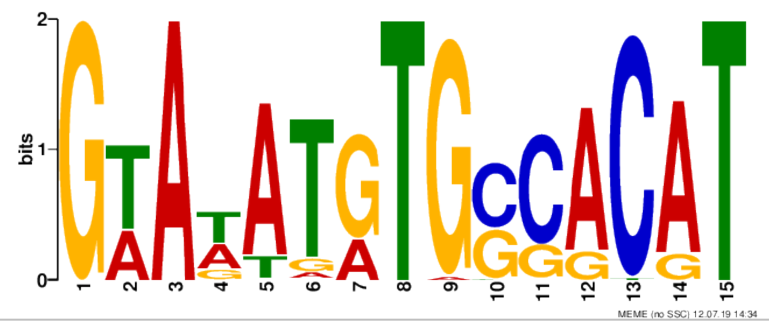	401
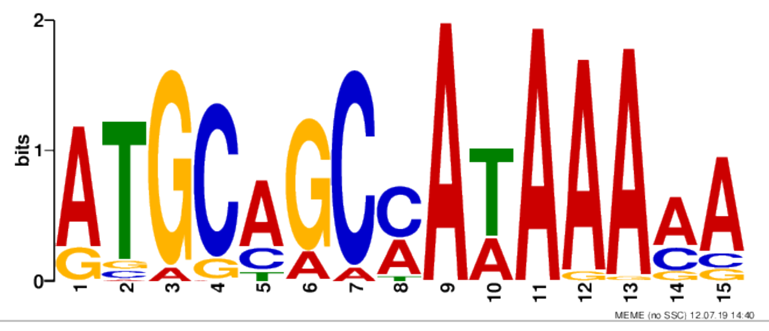	1692	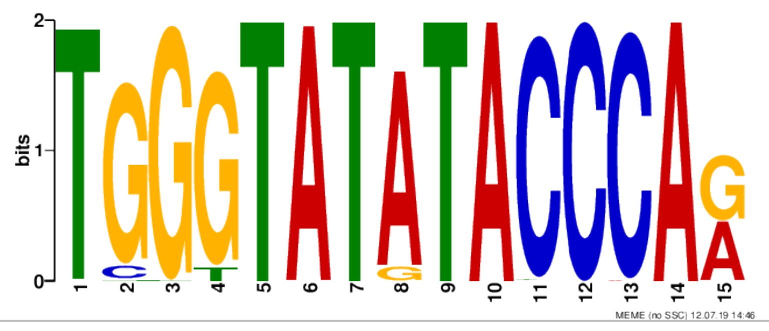	299	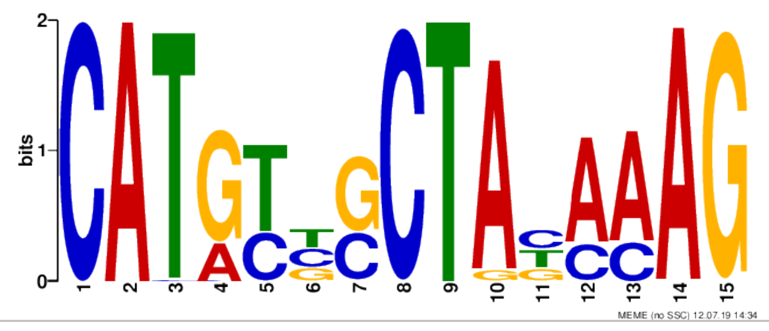	358
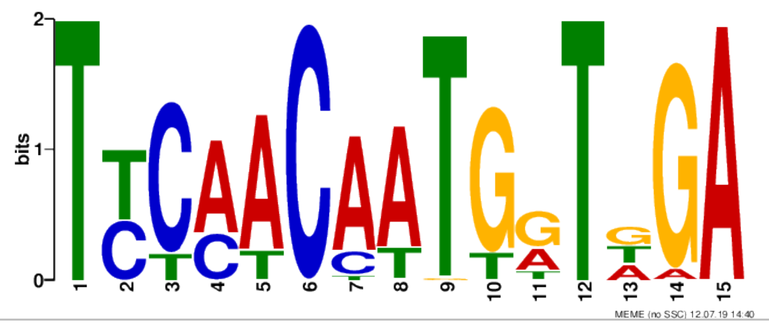	1446	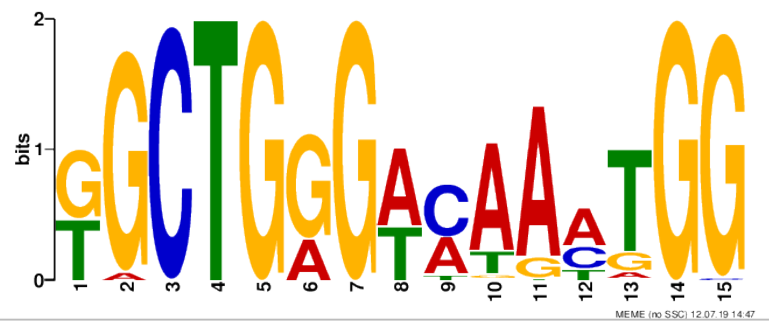	362	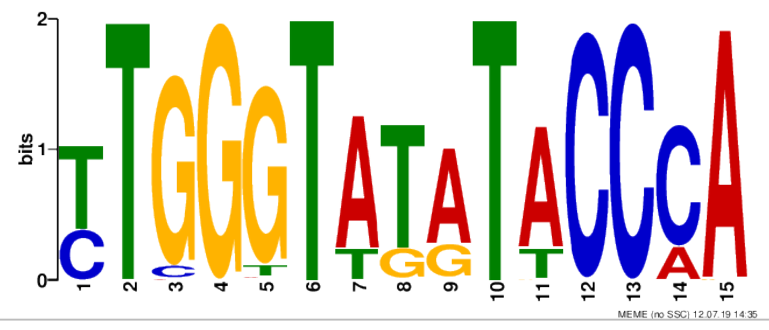	387
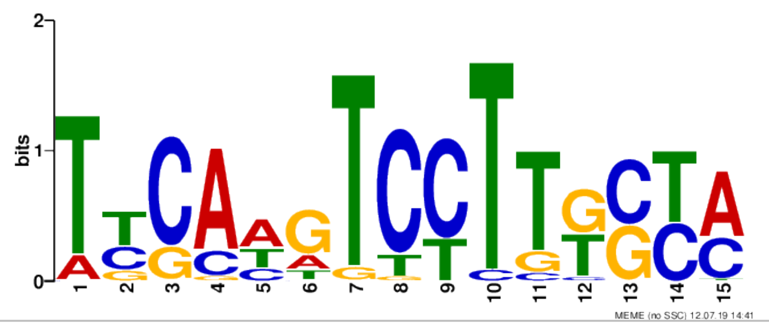	2939	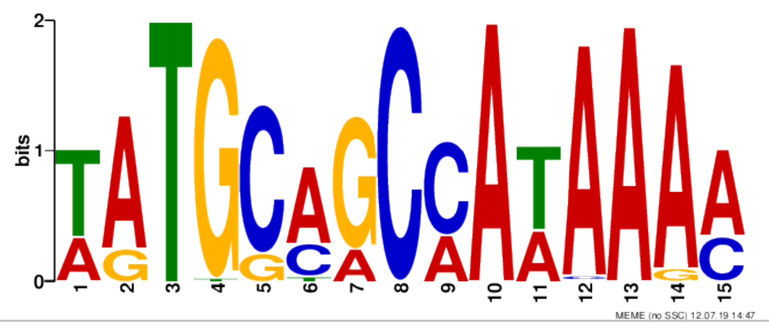	477	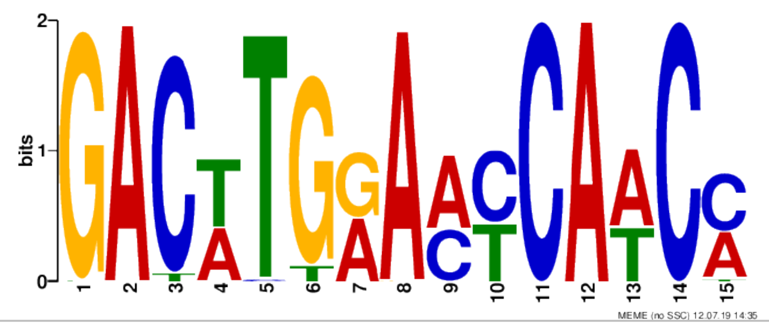	329
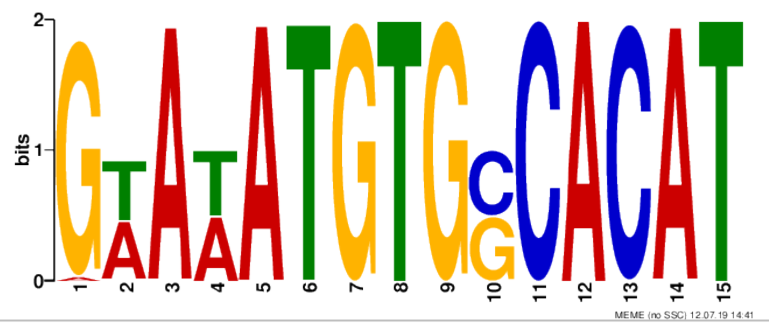	910	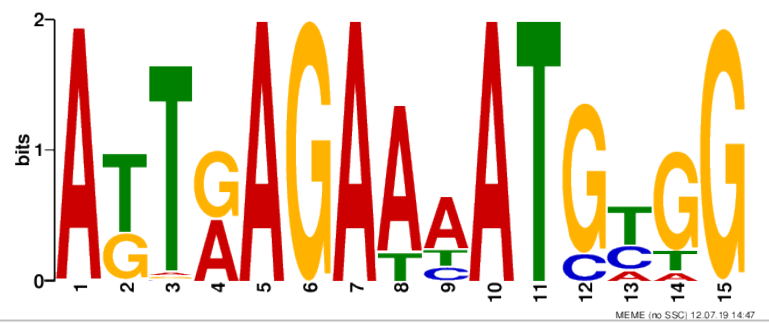	301	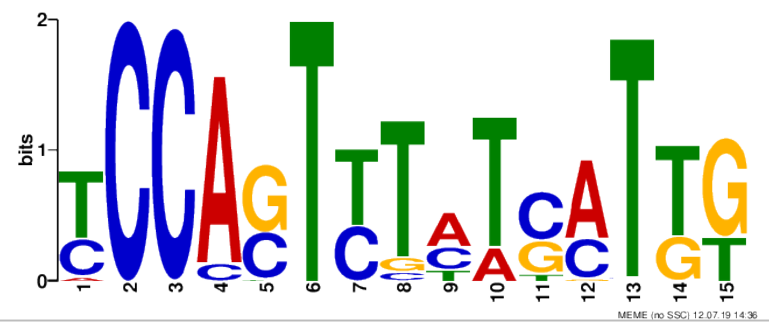	471
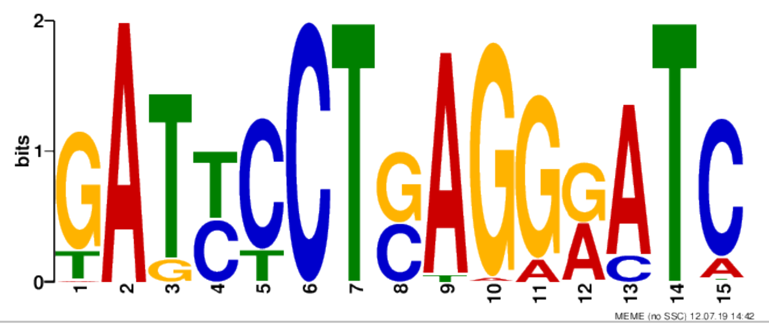	1209	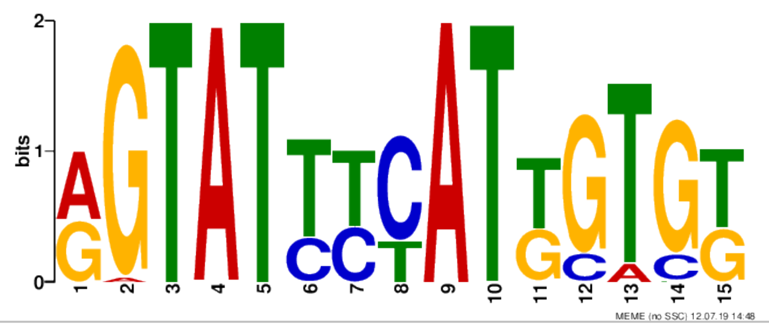	387	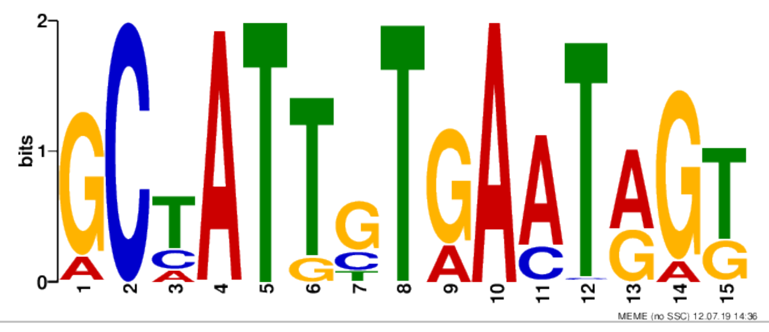	389
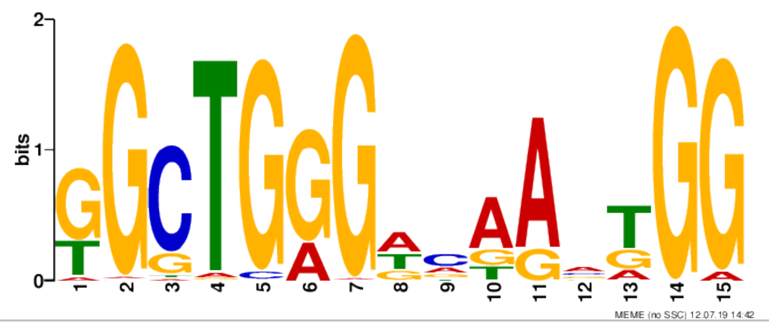	1520	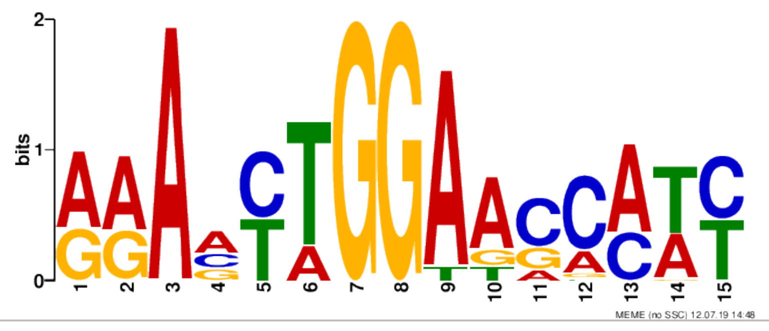	633	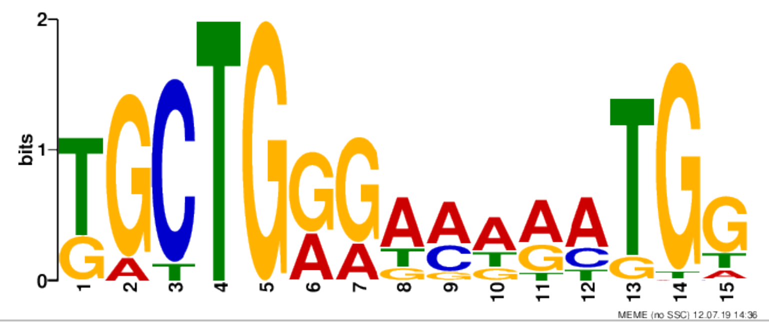	679
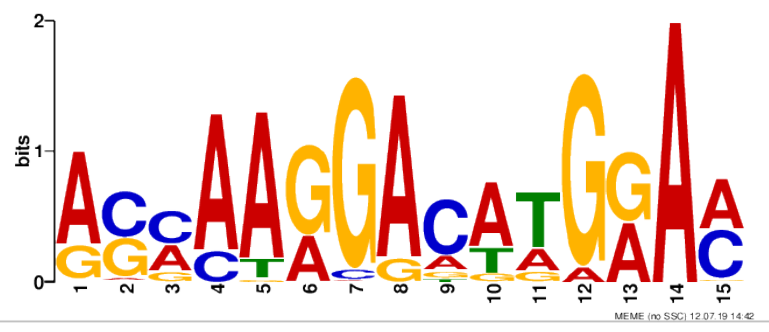	2129	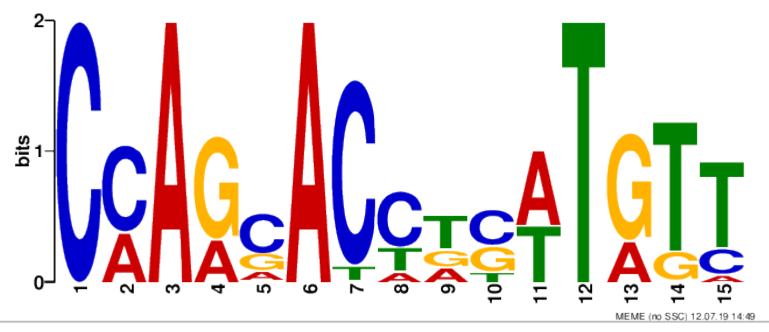	571	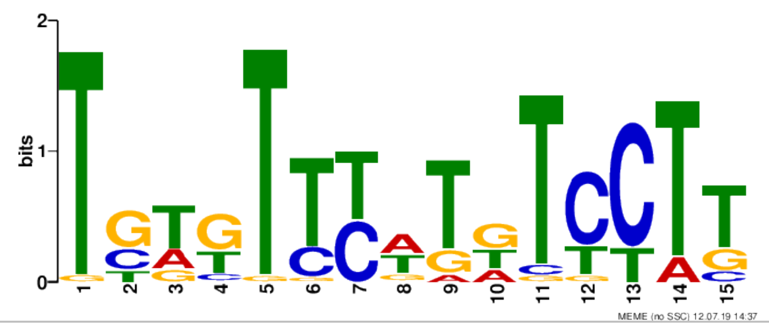	1311
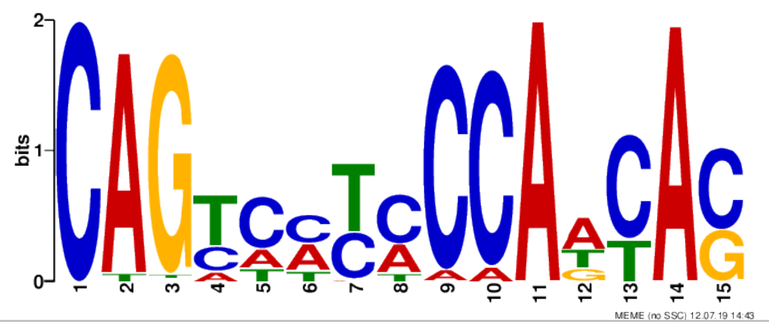	1394	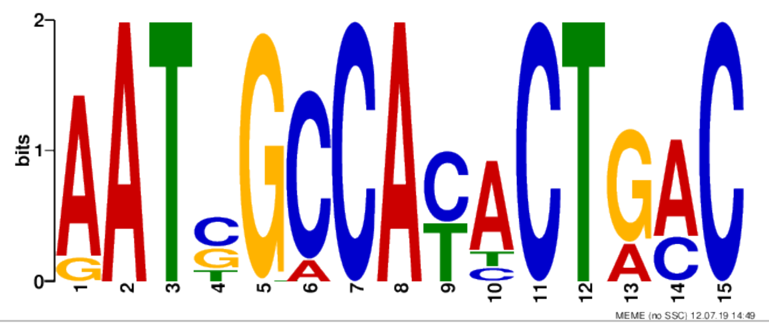	271	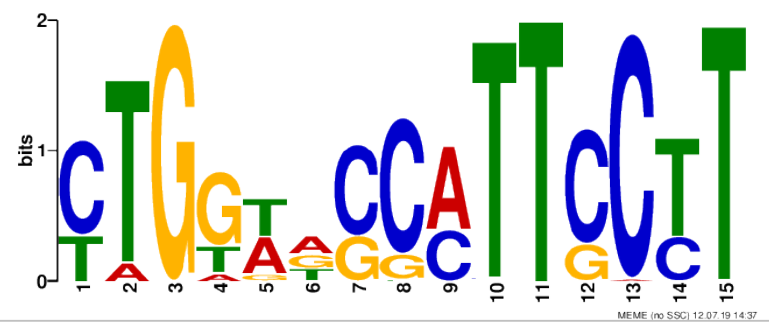	380

Motifs discovered in LINE-positive Motif-negative peaks for CT, KD and OE samples.

APPENDIX VI

"Table Analyzed" "Fig 3I"

"P value and statistical significance"
" Test" "Fisher's exact test"
" P value"   <0.0001
" P value summary"   ****
" One- or two-sided"   Two-sided
" Statistically significant (P < 0.05)?" Yes

"Data analyzed" "deltaAUC (KD-CT) without motifs" "deltaAUC (KD-CT) with motifs"   Total
" CT-KD peaks" 43386   24052   67438
" KD-CT peaks" 22801   17739   40540
" Total"   66187   41791   107978

"Percentage of row total" "deltaAUC (KD-CT) without motifs" "deltaAUC (KD-CT) with motifs"
" CT-KD peaks" 64.33% 35.67%
" KD-CT peaks" 56.24% 43.76%

"Percentage of column total"   "deltaAUC (KD-CT) without motifs" "deltaAUC (KD-CT) with motifs"
" CT-KD peaks" 65.55% 57.55%
" KD-CT peaks" 34.45% 42.45%

"Percentage of grand total"   "deltaAUC (KD-CT) without motifs" "deltaAUC (KD-CT) with motifs"
" CT-KD peaks" 40.18% 22.27%
" KD-CT peaks" 21.12% 16.43%

"Table Analyzed" "Fig 3J"

"P value and statistical significance"

" Test"	"Fisher's exact test"
" P value"	<0.0001
" P value summary"	****
" One- or two-sided"	Two-sided
" Statistically significant (P < 0.05)?"	Yes

"Data analyzed"	"deltaAUC (OE-CT) without motifs"	"deltaAUC (OE-CT) with motifs"	Total
" CT-OE peaks"	45072	29337	74409
" OE-CT peaks"	12407	37	12444
" Total"	57479	29374	86853

"Percentage of row total"	"deltaAUC (OE-CT) without motifs"	"deltaAUC (OE-CT) with motifs"
" CT-OE peaks"	60.57%	39.43%
" OE-CT peaks"	99.70%	0.30%

"Percentage of column total"	"deltaAUC (OE-CT) without motifs"	"deltaAUC (OE-CT) with motifs"
" CT-OE peaks"	78.41%	99.87%
" OE-CT peaks"	21.59%	0.13%

"Percentage of grand total"	"deltaAUC (OE-CT) without motifs"	"deltaAUC (OE-CT) with motifs"
" CT-OE peaks"	51.89%	33.78%
" OE-CT peaks"	14.29%	0.04%

"Table Analyzed" "Fig 3K"

"P value and statistical significance"
" Test" "Fisher's exact test"
" P value" <0.0001
" P value summary" ****
" One- or two-sided" Two-sided
" Statistically significant (P < 0.05)?" Yes

"Data analyzed"	"deltaAUC (KD-OE) without motifs"	"deltaAUC (KD-OE) with motifs"	Total
" KD-OE peaks"	25113	15337	40450
" OE-KD peaks"	19554	1390	20944
" Total"	44667	16727	61394

"Percentage of row total"	"deltaAUC (KD-OE) without motifs"	"deltaAUC (KD-OE) with motifs"
" KD-OE peaks"	62.08%	37.92%
" OE-KD peaks"	93.36%	6.64%

"Percentage of column total"	"deltaAUC (KD-OE) without motifs"	"deltaAUC (KD-OE) with motifs"
" KD-OE peaks"	56.22%	91.69%
" OE-KD peaks"	43.78%	8.31%

"Percentage of grand total"	"deltaAUC (KD-OE) without motifs"	"deltaAUC (KD-OE) with motifs"
" KD-OE peaks"	40.90%	24.98%
" OE-KD peaks"	31.85%	2.26%
